# Design Aspects of Doped CeO_2_ for Low-Temperature Catalytic CO Oxidation: Transient Kinetics and DFT Approach

**DOI:** 10.1021/acsami.1c02934

**Published:** 2021-04-09

**Authors:** Kyriaki Polychronopoulou, Ayesha A. AlKhoori, Angelos M. Efstathiou, Maguy Abi Jaoude, C. M. Damaskinos, Mark A. Baker, Alia Almutawa, Dalaver H. Anjum, Michalis A. Vasiliades, Abderrezak Belabbes, Lourdes F. Vega, Abdallah Fathy Zedan, Steven J. Hinder

**Affiliations:** † Department of Mechanical Engineering, Khalifa University of Science and Technology, Main Campus, Abu Dhabi 127788, UAE; ‡ Center for Catalysis and Separations, Khalifa University of Science and Technology, Main Campus, Abu Dhabi 127788, UAE; § Department of Chemistry, Heterogeneous Catalysis Lab, University of Cyprus, 1 University Avenue, University Campus, 2109 Nicosia, Cyprus; ∥ Department of Chemistry, Khalifa University of Science and Technology, Main Campus, Abu Dhabi 127788, UAE; ⊥ The Surface Analysis Laboratory, Faculty of Engineering and Physical Sciences, University of Surrey, Guildford GU2 4DL, U.K.; # Department of Physics, Khalifa University of Science and Technology, Main Campus, Abu Dhabi 127788, UAE; ¶ Research and Innovation Center on CO_2_ and H_2_ (RICH), and Chemical Engineering Department, Khalifa University, Abu Dhabi 127788, UAE; ∇ National Institute of Laser Enhanced Science, Cairo University, Giza 12613, Egypt

**Keywords:** binary metal oxides, ^18^O isotopic labeling, SSITKA-DRIFTS, DFT, oxygen mobility, ceria, transition metal, microwave, CO oxidation

## Abstract

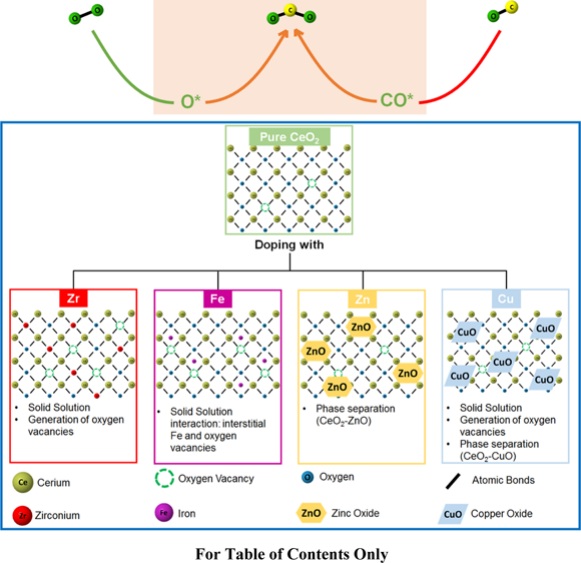

CO elimination through oxidation over highly active and cost-effective catalysts is a way forward for many processes of industrial and environmental importance. In this study, doped CeO_2_ with transition metals (TM = Cu, Co, Mn, Fe, Ni, Zr, and Zn) at a level of 20 at. % was tested for CO oxidation. The oxides were prepared using microwave-assisted sol–gel synthesis to improve catalyst’s performance for the reaction of interest. The effect of heteroatoms on the physicochemical properties (structure, morphology, porosity, and reducibility) of the binary oxides M–Ce–O was meticulously investigated and correlated to their CO oxidation activity. It was found that the catalytic activity (per gram basis or TOF, s^–1^) follows the order Cu–Ce–O > Ce–Co–O > Ni–Ce–O > Mn–Ce–O > Fe–Ce–O > Ce–Zn–O > CeO_2_. Participation of mobile lattice oxygen species in the CO/O_2_ reaction does occur, the extent of which is heteroatom-dependent. For that, state-of-the-art transient isotopic ^18^O-labeled experiments involving ^16^O/^18^O exchange followed by step-gas CO/Ar or CO/O_2_/Ar switches were used to quantify the contribution of lattice oxygen to the reaction. SSITKA-DRIFTS studies probed the formation of carbonates while validating the Mars–van Krevelen (MvK) mechanism. Scanning transmission electron microscopy-high-angle annular dark field imaging coupled with energy-dispersive spectroscopy proved that the elemental composition of dopants in the individual nanoparticle of ceria is less than their composition at a larger scale, allowing the assessment of the doping efficacy. Despite the similar structural features of the catalysts, a clear difference in the O_lattice_ mobility was also found as well as its participation (as expressed with the α descriptor) in the reaction, following the order α_Cu_ > α_Co_> α_Mn_ > α_Zn_. Kinetic studies showed that it is rather the pre-exponential (entropic) factor and not the lowering of activation energy that justifies the order of activity of the solids. DFT calculations showed that the adsorption of CO on the Cu-doped CeO_2_ surface is more favorable (−16.63 eV), followed by Co, Mn, Zn (−14.46, −4.90, and −4.24 eV, respectively), and pure CeO_2_ (−0.63 eV). Also, copper compensates almost three times more charge (0.37*e*^−^) compared to Co and Mn, ca. 0.13*e*^−^ and 0.10*e*^−^, respectively, corroborating for its tendency to be reduced. Surface analysis (X-ray photoelectron spectroscopy), apart from the oxidation state of the elements, revealed a heteroatom–ceria surface interaction (O_a_ species) of different extents and of different populations of O_a_ species.

## 1 Introduction

CO oxidation (CO-OX) catalytic processes are of crucial industrial and environmental importance. In the presence of hydrogen in the feed, the CO preferential oxidation (CO-PROX) process is used for the H_2_ fuel cleanup to appropriate levels for fuel cell applications. In contrast, without H_2_ in the feed, the CO-OX process is equally important as it targets the elimination of CO which is hazardous, reaching lethal levels at only 650–700 ppm.^1^ CO oxidation catalysts are typically designed to fulfill a number of desired physicochemical characteristics, including (i) a wide operating temperature range (25–400 °C), (ii) unnecessary activation prior to use, (iii) prolonged lifetime, and (iv) capability for regeneration.^1^ An extensive number of CO oxidation catalysts have been reported, most of which are based on precious metals or transition metal (TM) oxides (e.g., Au, Pt, Rh, Ru, and Pd or Cu and Co).^1^ The metal oxide used as a carrier of noble metal plays also an important role in the activity of supported metal catalysts.

Due to the high cost of noble metals and their vulnerability to poisoning,^1^ research has been focused on finding alternative active and cost-effective CO oxidation (COOX) catalysts. TM oxides, such as cobalt oxide, have been tested for CO oxidation and exhibited notable activity, very close to that of noble metals.^1^ Furthermore, copper, nickel, manganese, and iron TM oxides have been studied for CO oxidation but demonstrated lack of stability and high susceptibility to deactivation in the presence of CO_2_ and H_2_O in the feed gas stream.^1^ Mixed metal oxides gained greater attention due to their enhanced mechanical stability, selectivity, and reducibility.^2^

Ceria has gained great interest as a CO cleanup catalyst due to its ability to store and release oxygen rapidly. The latter is due to the energetically favorable redox cycle Ce^3+^ ↔ Ce^4+^^3^ and its ability to prevent sintering (or agglomeration) of the supported metal or metal oxide phase by developing strong surface bonding interactions. Due to the low thermal stability of pure ceria, enhancement of its activity and thermomechanical properties occurs through the introduction in its crystal structure (doping effect) of low-valence, high-valence, or same-valent dopant, named lower valence dopants (LVD), HVD, or SVDS, respectively.^3^ Notable improvements in catalytic performance due to significant enhancement in the concentration of oxygen vacant sites and oxygen mobility (or ionic conductivity) were observed with doped-ceria systems, regardless of the experimental conditions.

The introduction of a heteroatom in the ceria matrix induces a variety of synergy phenomena, such as solid solution formation and contact between segregated oxides and metal deposition over the surface.^3^ These cause an enhanced activity of doped ceria by several orders of magnitude. The method of synthesis has been proven to play a significant role in the final properties of doped ceria. Among the ceria-based synthetic methods available, microwave synthesis has been applied to typically reduce the energy penalty and processing time, while providing a controlled reaction and enhancing the quality of the doped-ceria materials produced.^4^ Some additional advantages of the microwave method include overall simplicity, easily available medium, high yield, better control over material morphology, optimum crystallization due to localized hotspots, and homogeneous temperature distribution (no gradients). The homogeneous heating is the drive toward uniform nanoparticles, whereas the fast kinetics in the microwave synthesis plays a major role in the formation of ultrafine particles. It has also been suggested that while in solution, the nanoparticles tend to absorb more microwave radiation on their surface, compared to their core, thus leading to local overheating and resulting in high particle surface energy and reactivity.^4^

Deep insights into the oxygen mobility and oxygen vacant site formation, as well as the in situ monitoring of the O_L_ (lattice oxygen) participation in the CO oxidation, can be obtained by performing advanced transient isotopic studies. This has been reported so far for certain ceria-based oxides,^5^ CeO_2_ nanocrystals,^6^ and supported metal catalysts (e.g., Au/CeO_2_).^7^ Penkala et al.^5^ investigated the oxygen uptake/release in operando CO oxidation (dynamic redox conditions) after using isotope labeling pulse temperature-programmed oxidation reaction on ^18^O-pretreated doped ceria. The authors also examined the dynamic changes in the ceria oxygen sublattice under the ^16^O/^18^O step-gas switch by Raman spectroscopy. The rate of ^18^O/^16^O exchange during oxidation of carbon monoxide in the absence and in the presence of oxygen in the feed gas stream was investigated by Gamboa-Rosales et al.^8^ on ceria-supported Au–CuO and Au–Co_3_O_4_ catalysts. The oxygen exchange followed the order CoCe ≈ AuCoCe < CuCe ≈ AuCuCe < Ce ≈ AuCe. It was demonstrated that lattice oxygen contributes either in the CO oxidation or in the exchange reaction between the ^16^O in CO or CO_2_ products with ^18^O depending on the catalyst’s composition and structure.

As opposed to experimental studies, theoretical studies on doped ceria regarding the influence of the metal heteroatom on the oxygen vacancy formation and oxygen mobility are still very scarce, particularly on the non-lanthanide, TMs. The majority of these studies refer to a single dopant in the ceria matrix,^9^ whereas studies on a series of dopants are most frequent for the lanthanide f-elements.^10^ It was shown that oxygen vacancies are highly favorable in the presence of dopants, and their formation is accompanied by lattice expansion due to the Ce^4+^ → Ce^3+^ reduction of two neighboring Ce atoms in the Ce–O_v_–Ce chain (O_v_ represents an oxygen vacancy). The formation of vacancies stabilizes the doped-ceria structure, whereas a dopant–vacancy complex formation has been reported for Cu and Gd dopants,^11^ leading to the formation of small dopant oxide clusters that escape XRD detection.

Density functional theory (DFT) calculations can be used in order to shed light on the impact of the different heteroatoms on the structure and on the CO oxidation performance. However, the application of the generalized gradient approximation DFT methods has raised some concerns for the metal oxide systems, due to the underestimation of important parameters, such as the chemical reactions’ barriers, band gaps of materials, dissociation energies of molecular ions, and so forth. In addition, DFT methods can lead to the overestimation of binding energies of charge transfer complexes. All these limitations have a common route.^12^ The origin of the problem is that the standard DFT methodology lacks the correct description of electron localization essentially due to the self-interaction error which basically includes a nonhomogenous charge density due to the electron–electron interactions. The latter comes with a repulsion that is detrimental in the correct description of point defects (e.g., oxygen vacancies in ceria) using the standard DFT method. A rather popular way to tackle this DFT deficiency is the use of the Hubbard *U* parameter as it corrects the position of some orbitals/characteristics of the electronic structure. The idiosyncrasy of the *U* parameter originates from its empirical nature, and it always has to be converged to a property that has been tested experimentally or theoretically (for instance, with fully ab initio calculations).

In particular, in the case of ceria, a *U* potential is considered only for the Ce f-states, whereas in the case of many metal oxides, *U* corrections are applied on the heteroatom d-states for the accurate prediction of material properties. In addition, a *U* value for the p-states of oxygen can improve the estimation of band gaps and energy of reduction for ceria.^12^ Depending on the atom geometry (e.g., close to the surface vs subsurface layers and near heteroatom vs far from heteroatom), a different *U* potential can be selected by introducing an additional difficulty to the calculations. As the *U* parameter selection is not uniform, meaningful comparisons among different DFT calculations of M-doped CeO_2_ reactivity can be challenging. The importance of the *U* correction factor in the DFT + *U* calculations was demonstrated by Krcha et al.^13^ not only for the f-states of Ce but also for the d-states of metal. Particularly, for d-metal dopants, Krcha et al.^14^ used DFT + *U* calculations to study the electronic effect of d-metal dopants on ceria reducibility, where a Sabatier volcano-type behavior for the oxidation activity of metals versus the d-metal was established. The authors reported that the metal dopant may have two distinct roles to play, modify the redox properties of ceria or reduce itself. The latter mechanism explains the electronic interactions between the metal dopant and ceria. Among the M-doped CeO_2_ (M = Mn, Pr, Sn, and Zr) systems, it was found that all dopants tend to lower the energy for oxygen vacancy formation. In particular, Mn-doped CeO_2_ has the lowest O-vacancy formation energy, ca. −0.434 eV for the 1NN configuration, which corroborates for a facile formation of an O vacancy in Mn-doped CeO_2_, and this can be linked with the small size of Mn^4+^ (0.52 Å). Along the same lines, Kang et al.^15^ reported that Mn^2+^ is the oxidation state of the dopant (Mn-doped ceria) due to its size compatibility with Ce^4+^. Therefore, the low oxygen vacancy formation energy is due to structural and electronic effects in agreement with the study by Andersson et al.,^16^ who concluded that there is a strong correlation between the dopant ionic radius and the vacancy formation energy. The distortions in the bulk structure of CeO_2_ were studied by Gupta et al.^17^ for different TM dopants (TM: Mn, Fe, Co, Ni, and Cu) at the 10 at. % dopant level. It was reported that as the M–O bond length increases, the M–Ce–O mixed oxide oxygen storage capacity, OSC (higher reducibility), becomes higher. Several experimental^17^ and theoretical studies^13^ have examined how the presence of one or the co-presence of two TMs, as dopants/co-dopants, affects the catalytic activity of the doped-ceria surface.

The present work aims to unveil material design aspects of the catalytic CO oxidation performance of ceria when alio-valent doping, at a rather high level (20 at. %), takes place. A suite of d-cations were used as oxidation activity descriptors, namely, Co^2+^, Cu^2+^, Ni^2+^, Zn^2+^, Fe^3+^, Mn^4+^, and Zr^4+^. The novelty of this work lies on the attempted systematic correlation of the intrinsic properties of the structurally isomorphic metal oxides, such as oxygen mobility and redox properties with their catalytic CO oxidation performance. The latter was taken as a probe reaction under both aerobic and anaerobic conditions, and TOF (s^–1^) rates were estimated based on initial rates estimated from transient isotopic kinetic experiments and the concentration of surface oxygen species, for the first time to the best of our knowledge. Particular mechanistic aspects of the CO oxidation reaction, such as the chemical nature of surface active intermediates (e.g., carbonates and lattice oxygen), are probed through state-of-the-art SSITKA-DRIFTS, ^18^O-TIIE, and transient CO reactivity studies with the lattice oxygen of doped-ceria materials. The experimental results are supported by ab initio calculations. To the best of our knowledge, these aspects of the CO oxidation reaction are highlighted for the first time, advancing the state of the art of the design of ad hoc catalysts for this purpose.

## 2 Materials and Methods

### 2.1 Doped-CeO_2_ Catalytic Material Preparation

The microwave-accelerated reaction system (MARS-6) for material synthesis was used in this study and has been described elsewhere.^18^ The doped-ceria materials were prepared by dissolving the precursor salts Ce(NO_3_)_3_·6H_2_O and M(NO_3_)_3_·*x*H_2_O (M = metal heteroatom) in distilled water with the respective molar ratios for obtaining the final composition of M–Ce–O, where M: Fe, Mn, Ni, Co, Zn, Cu, and Zr and nominal M/Ce ratio: 0.25. Citric acid was used as a complexing agent. Calcination in static air, ca. 500 °C for 6 h under atmospheric pressure, was performed in all samples. More details on the applied synthesis and calcination procedures applied are provided in the Supporting Information.

### 2.2 Doped-CeO_2_ Catalytic Material Characterization

Powder X-ray diffraction (XRD), scanning electron microscopy, transmission electron microscopy (TEM), scanning TEM (STEM) with high-angle annular dark field imaging (HAADF), H_2_-temperature-programmed reduction (H_2_-TPR), CO_2_-temperature-programmed desorption (CO_2_-TPD), X-ray photoelectron spectroscopy (XPS), and N_2_ adsorption–desorption at 77 K were used to study the textural, structural, and surface physicochemical properties of the M–Ce–O solid catalysts. Prior to any characterization measurement, the samples were calcined at 500 °C for 6 h. The instrumentation details and experimental procedures on the application of these characterization techniques are provided in the Supporting Information.

### 2.3 Steady-State Catalytic CO Oxidation Performance Studies

The catalytic oxidation of carbon monoxide over the various doped-ceria (mixed metal oxides) solid materials was carried out in an apparatus described elsewhere.^18^ Details of the apparatus and the experimental conditions applied are provided in the Supporting Information. The CO conversion under steady-state reaction conditions was estimated based on material balance after using eq 1
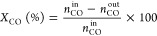
1where *X*_CO_ (%) is the percentage conversion of CO and *n*_CO_^in^ and *n*_CO_^out^ are the CO molar flow rate (mol/s) in the inlet and outlet feed gas streams, respectively. The absence of external and internal mass transport resistances was checked by applying the testing procedures described elsewhere.^19^

### 2.4 Catalytic CO Oxidation—Kinetic Studies

The power law kinetic rate expression given by eq 2 was used to estimate the apparent activation energy (*E*_a_, kJ mol^–1^), the pre-exponential factor (*A*) of the apparent rate constant (*k*_a_), and the reaction orders *x* and *y* with respect to CO and O_2_, respectively, for the catalytic CO oxidation over the CeO_2_, Zn–Ce–O, Mn–Ce–O, and Cu–Ce–O solids.
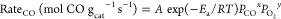
2

The feed gas composition 4% CO/20% O_2_/He and the temperature range of 120–270 °C were used to estimate the *E*_a_ and *A* kinetic parameters. The mass of the catalyst sample was varied in the 1–50 mg range, and the total volume flow rate of the feed gas stream was varied in the 50–200 cm^3^ min^–1^ range in order to keep the CO conversion below 15% in all kinetic experiments. At the same time, under the applied experimental conditions, the criteria for the absence of external (within the catalyst bed) and internal (within the catalyst pore volume) mass transport resistances were satisfied according to the literature.^19^ Thus, intrinsic kinetic rate data were gathered and correctly interpreted.

For the determination of the reaction order x with respect to CO, the partial pressure of the latter was varied in the 0.01–0.06 bar range, while keeping the O_2_ partial pressure constant at 0.2 bar (total pressure 1 bar). For the determination of the reaction order y with respect to O_2_, the partial pressure of the latter was varied in the 0.15–0.2 bar range, while keeping the CO partial pressure constant at 0.04 bar (4 vol % CO). A temperature of 150 °C was used for the Cu–Ce–O catalyst, while that of 210 °C was used for the CeO_2_, Zn–Ce–O, and Mn–Ce–O catalysts. The very high activity of Cu–Ce–O and the relatively much lower activity of the other three solids could not allow us to experimentally determine the *x* and *y* kinetic parameters at the same reaction temperature of interest.

### 2.5 Transient ^18^O/^16^O Isothermal Isotopic Exchange for Probing Participation of Lattice Oxygen in the CO Oxidation Reaction

^18^O/^16^O transient isothermal isotopic exchange (^18^O_2_-TIIE) experiments were performed to study the oxygen mobility/diffusion on the surface and in the bulk of the doped-ceria mixed metal oxides under dynamic conditions. The amount of each sample used was 50 mg, and the total volume flow rate of the feed gas stream (2 vol % ^18^O_2_/2 vol % Kr/Ar) was 50 N mL min^–1^. The samples were pretreated in 20 vol % ^16^O_2_/He at 500 °C for 3 h, followed by 10 min Ar purge. The ^18^O_2_-TIIE involves the following sequence of step-gas switches: 20 vol % ^16^O_2_/He → Ar → 2 vol % ^18^O_2_/Ar (500 °C, 15 min). During the latter step-gas switch, the transient response curves of the three oxygen isotopologues and that of Kr tracer (inert gas) were continuously monitored using an online mass spectrometer (MS, Balzers, Omnistar, 1–300 amu) using the mass numbers, *m*/*z*: 32, 34, 36, and 84 for ^16^O_2_, ^16^O^18^O, ^18^O_2_, and Kr, respectively. During the isotopic ^18^O_2_/Ar step-gas switch, ^16^O/^18^O exchange reactions take place between ^18^O_2_ and ^16^O-s (surface lattice oxygen) and diffusion of ^18^O-s on the surface/bulk of the doped-CeO_2_ metal oxide. For the latter step, ^16^O_b_ (lattice oxygen in the bulk) diffuses from the bulk of the solid metal oxide toward the surface.

The transient rate (μmol g^–1^ s^–1^) of ^18^O_2_(g) consumption (due to the exchange with ^16^O-s) is given by eq 3 after applying the appropriate material balance for the CSTR microreactor used. It should be noted that the accumulation term (last term in the right-hand side of eq 3) was found to be negligible compared to the other terms of eq 3. The amount of oxygen exchanged (mol ^18^O g^–1^) was estimated after integration of R^18^O_2_(*t*) given by eq 3 with time.
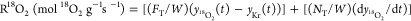
3in eq 3, *F*_T_ is the total molar flow rate (mol s^–1^) of the feed gas stream, *y*_^18^O_2__ and *y*_Kr_ are the mole fractions of ^18^O_2_ and Kr at the outlet of the CSTR microreactor, respectively, *N*_T_ is the total number of mols in the CSTR reactor, and *W* is the amount of sample used (ca. 50 mg).

A 4 vol % CO/Ar (anaerobic), 4 vol % CO/15 vol % O_2_/Ar (aerobic), or 0.75 vol % CO_2_/Ar gas treatment followed the partial exchange of support’s lattice ^16^O with ^18^O. This allowed us to investigate the participation and the extent of contribution of support’s lattice oxygen in the catalytic CO oxidation reaction. During the CO/Ar gas treatment, the formation of C^16^O^18^O(g), C^16^O_2_(g), and C^18^O_2_(g) illustrates that ^18^O-L lattice oxygen of doped ceria is able to react with CO-s or CO(g) toward CO_2_ formation, according to the following eqs 4–7
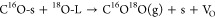
4
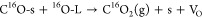
5
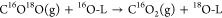
6
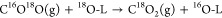
7

During the CO_2_/Ar gas treatment, eq 8 is applied
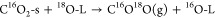
8where s is a catalytic site on the doped-ceria surface, ^18^O-L is a lattice ^18^O, and V_O_ is a surface oxygen vacancy in the doped-ceria material.

The ^16^O/^18^O oxygen exchange conducted at 500 °C was followed by cooling to 250 °C in the flow of the ^18^O_2_ isotopic gas mixture and a switch to Ar gas for 15 min. Subsequently, the gas flow was switched to 4 vol % CO/2 vol % Kr/Ar (250 °C, 20 min), where the *m*/*z* values of 28 (C^16^O), 44 (C^16^O_2_), 46 (C^16^O^18^O), and 48 (C^18^O_2_) were continuously monitored with online MS. Calibration of the C^16^O_2_ signal (conversion to concentration, mol %) was made using a certified calibration gas mixture (2.55 vol % CO_2_/Ar), which was also used for the calibration of C^16^O^18^O (*m*/*z* = 46) and C^18^O_2_ (*m*/*z* = 48) MS signals. The difference in sensitivities of C^16^O_2_ (*m*/*z* = 44) and C^18^O_2_ (*m*/*z* = 48) gases is within 10%. It should be noted that all transient response curves were reproducible in shape and position in time within better than 2%.

### 2.6 Steady-State Isotopic Transient Kinetic Analysis with DRIFTS (SSITKA-DRIFTS)

DRIFT spectra were recorded before and after the SSITKA step-gas switch 4 vol % ^12^CO/20 vol % O_2_/Ar (250 °C, 50 cm^3^/min, 30 min) → 4 vol % ^13^CO/20 vol % O_2_/Ar (250 °C, 50 cm^3^/min, 5 min), where a PerkinElmer Frontier FT-IR spectrometer (256 scans per spectrum, resolution of 4 cm^–1^, scan speed of 2 cm/s) equipped with a high-temperature/high-pressure temperature-controllable DRIFTS cell (Harrick, Praying Mantis) was used. The catalyst sample (∼80–100 mg) in a very fine powder form was placed firmly into the ceramic cup of the DRIFTS cell and pretreated in 20 vol % O_2_/He at 500 °C for 2 h. The catalyst sample was then cooled in Ar gas flow to 250 °C, which was then switched to Ar, and the spectrum of the solid was recorded at 250 °C. The latter spectrum was subtracted from the spectrum of the solid recorded under the isotopic or nonisotopic gas mixture at 250 °C. Details of the SSITKA and SSITKA-DRIFTS techniques can be found elsewhere.^20,21^ The power of this SSITKA-DRIFTS technique is that the chemical nature of active reaction intermediates and that of inactive species during a gas–solid heterogeneous catalytic reaction can be probed based on the appearance or not, respectively, of the red isotopic shift of relevant vibrational modes of the active or inactive adsorbed species.

### 2.7 DFT Calculations

The structural and electronic properties of chemisorbed CO on the doped-CeO_2_ surface with TMs, TM: Mn, Co, Cu, and Zn, were investigated by performing spin-polarized DFT calculations, as implemented in the Quantum-ESPRESSO package.^22^ Details of the DFT calculations can be found in the Supporting Information.

## 3 Results and Discussion

### 3.1 Structural and Textural Studies of Doped-CeO_2_ Catalytic Materials

**Figure 1:**
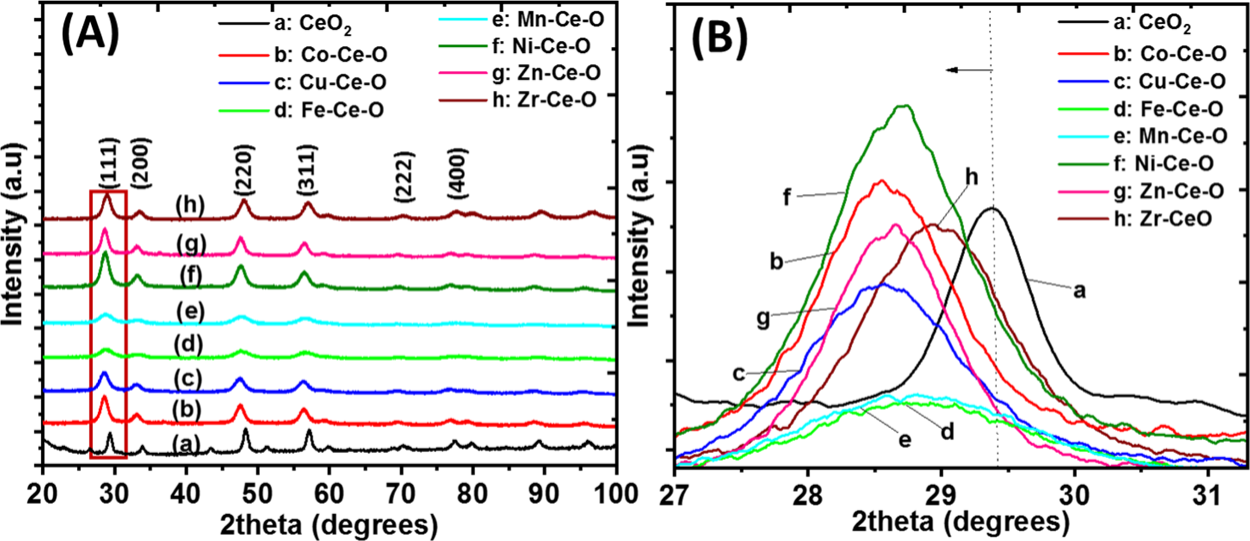
(A) Powder XRD patterns of ceria and doped-ceria systems (a–h). (B) Powder XRD patterns of ceria and doped-ceria systems (a–h) in the 27–31 2θ range.

Powder XRD patterns recorded for all samples (Figure 1A) show six diffraction peaks which correspond to the (111), (200), (220), (222), (311), and (400) facets of a typical fluorite cubic structure of CeO_2_. The diffraction peaks for pure ceria (2θ = 29.7, 33.8, 48.4, 57.1, 59.8, and 70.5°) are slightly shifted toward lower Bragg angles upon doping of ceria with different metal cations, as shown in Figure 1B. In the case of Co-doped ceria, a shift in the ∼28.6–29° range is observed, which at a first glance indicates the incorporation of the heteroatom cation into the ceria lattice, thus forming a solid solution. The solid solution formation appears to be the case for all the TM heteroatoms used except for the case of ZnO (Figures S1A,S2,S3) since no characteristic diffraction peaks of the TM oxide were identified (Figure 1A). Conversely, solid nanoparticles smaller than 4 nm in size and of low concentration (wt %) cannot be resolved from the recorded diffractograms (Rietveld analysis is required). The XRD results shown in Figure 1A,B, however, give the first view of the structure. A thorough multiscale structural analysis involving other techniques, such as EXAFS,^23^ is necessary. Therefore, this leaves some space to speculate the presence of a highly dispersed dopant TM oxide, which escapes the powder XRD detection.

High dispersion of heteroatoms into the ceria lattice has been reported by us at much higher metal loadings (>40 at. %) compared to the moderate heteroatom level used in the present work (ca. 20 at. %).^24^ The latter can be ascribed partially to the beneficial role of microwave synthesis, which allows the thorough mixing of the constituents’ elements. The mean crystallite size of the solids was calculated using the Scherer equation, and this was found to be in the 8.8–16.4 nm range (see Table 1).

**Table 1:** Structural and Textural Characteristics of the Various TM-Doped Ceria Solids

composition M–Ce–O	heteroatom size (Å)	crystallite size (nm)	lattice strain^c^	lattice parameter (Å)	BET area (m^2^/g)	cumulative pore volume (cm^3^/g)	F_2g_ band/O_v_/F_2g_ ratio
CeO_2_	Ce^3+^: 1.15	25.2^a^	0.00197	5.82	14.8		464/0
	Ce^4+^: 0.97	(15.8)^b^					
Co–Ce–O	Co^2+^: 0.79	16.35^a^	0.00184	5.42	11.9	0.05	442.6/0.026
		(7.40)^b^					
Cu–Ce–O	Cu^2+^: 0.87	11.07^a^	0.00494	5.31	16.2	0.085	446.6/0.050
		(8.01)^b^					
Fe–Ce–O	Fe^3+^: 0.78	8.81^a^	0.00865	5.35	26.0	0.083	444.1/0.025
		(6.80)^b^					
Mn–Ce–O	Mn^4+^: 0.53	9.87^a^	0.01149	5.42	63.0	0.13	442.3/0.032
	Mn^3+^: 0.65	(7.40)^b^					
Ni–Ce–O	Ni^3+^: 0.72	12.43^a^	0.00447	5.43	13.3	0.051	442.9/0.054
		(9.14)^b^					
Zn–Ce–O	Zn^2+^: 0.88	13.86^a^	0.00236	5.37	11.1	0.064	462.5/0.012
		(8.63)^b^					
Zr–Ce–O	Zr^4+^: 0.80	10.20^a^	0.00202	5.69	61.2	0.090	472.3/0.036
		(6.66)^b^					

a Calculated based on the Scherrer formula.

b Calculated based on the Williamson–Hall method.

c Calculated based on the Williamson–Hall strain plot.

As mentioned above, the powder XRD pattern of Zn–Ce–O (Figure S1A) revealed weak diffraction peaks which might correspond to the hexagonal ZnO structure, namely, the (100) at 32° and (101) at 36° facets. Previous studies on 10 mol % Zn–ceria prepared via a pseudo sol–gel method showed a powder XRD pattern with reflections belonging to the ZnO phase.

The broad XRD pattern obtained for Fe–Ce–O and Mn–Ce–O demonstrates their weak crystalline nature compared to the rest binary TM-doped ceria systems, as also indicated by the small crystallite size obtained (Table 1). The different crystal structures adopted by the M–Ce–O mixed metal oxides (M = Fe and Mn) show poor solubility of Fe^3+^ and Mn^4+^ in the ceria fluorite cubic lattice of Fe–Ce–O and Mn–Ce–O binary oxides, which eventually appears as peak broadening. A decrease in their lattice parameter was also found due to the guest’s (Mn^4+^ and Fe^3+^) smaller ionic radius, which causes unit cell shrinkage (see Table 1). Limited solubility of Fe in the Ce–Fe–O system has been reported in a previous work.^25^ Many studies investigated the maximum Fe loading in the Ce–Fe–O system that would allow the formation of solid solution. For example, 10 at. % Fe was reported as the highest content for Ce–Fe–O prepared by the coprecipitation method at basic pH (8–10) and that of pseudo sol–gel.^25^ Taking into account previously mentioned findings and given that microwave synthesis is expected to favor homogenous mixing between the heteroatom and ceria, thereby allowing the formation of solid solution at a much higher heteroatom content, the question of solid solution formation is still open.

The lattice parameter (Table 1) of all samples increases with the ionic size of the cation introduced in the ceria lattice, according to Bragg’s law. All of the heteroatoms possess a smaller ionic radius than Ce^4+^, which causes shrinkage of the mixed metal oxide lattice (solid solution). This leads to a smaller lattice parameter than in CeO_2_ and, as a consequence, introducing lattice strain in the structure. The lattice strain was estimated using the Williamson–Hall analysis (Table 1 and Figure S4). All solids presented tensile lattice strain with the Mn–Ce–O and Fe–Ce–O having the highest values (Table 1).

**Figure 2:**
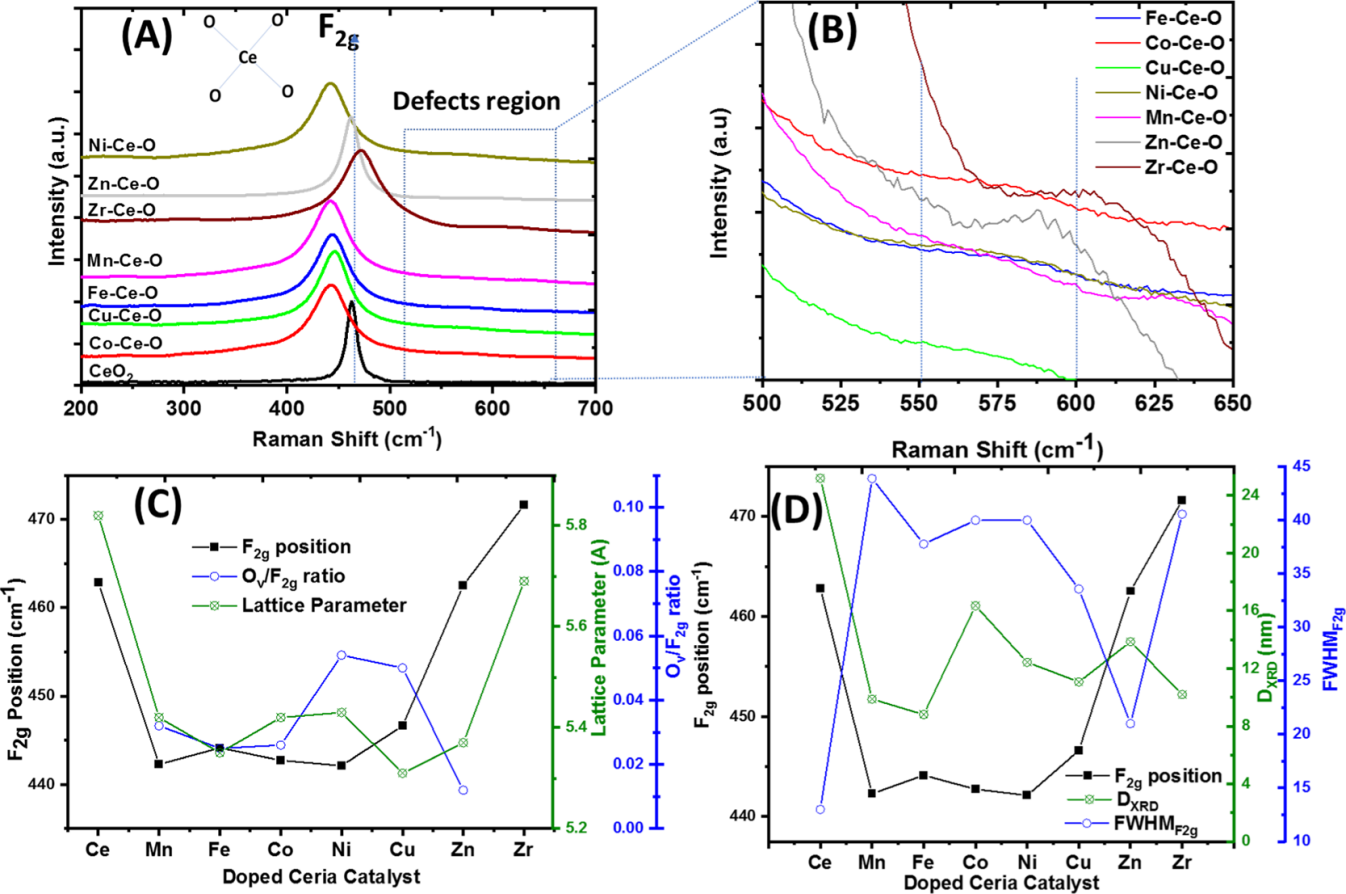
(A) Raman spectra of the representative doped-ceria catalysts, (B) zoom-in of the Raman spectra from (A) in the 500−650 cm^−1^ region, (C) F_2g_ band position along with the O_v_/F_2g_ ratio and lattice parameter (Å) for the doped-ceria catalysts, and (D) *D*_XRD_ and FWHM_F_2g__ for the doped ceria catalysts.

Raman is very sensitive to the oxygen sublattice and point defects in a given metal oxide system, and it can provide information on the oxygen chemical environment, as well as the presence of oxygen vacancies in mixed metal oxide systems. According to the literature,^26^ CeO_2_ Raman spectra are characterized by the presence of a F_2g_ vibrational mode at ∼464 cm^–1^, which corresponds to the symmetric breathing mode of the oxygen atoms surrounding Ce^4+^ cations (Figure 2A). Similar Raman spectra in terms of their characteristic features were observed for all the studied solid doped-ceria catalysts. Upon doping of ceria, translation symmetry of the ceria lattice is disturbed due to defects and anionic vacancy formation, which lead to violation of the *k* = 0 momentum conservation rule for Raman scattering to be observed. Thus, phonons across the whole Brillouin zone start contributing to the Raman spectrum, leading to many broad and low intensity bands. For the predominant band, F_2g_ at 465 cm^–1^, broadening, decrease in intensity, and a blue shift/red shift are noticed. What dictates the F_2g_ shift is the lattice contraction along with Ce–O bond softening, two major and competing effects.

The charge compensation mechanism upon doping with a cation of lower charge is responsible for the creation of oxygen vacancies that appeared in the 540–600 cm^–1^ range. As illustrated in Figure 2A, the F_2g_ band appears for representative compositions studied herein, suggesting that the fluorite cubic structure of ceria is maintained. This agrees with the powder XRD results. The decrease in the crystallite size is also in agreement with the powder XRD studies (Figure 1A). The smaller crystallite size obtained through doping and the lattice parameter and symmetry change compared to pure ceria are both associated with the broadening and shift (red of blue shift) of the F_2g_ band.^27^ A different extent in the F_2g_ shift is highly dependent on the chemical nature of the heteroatom, as it reflects the TM–CeO_2_ interactions and the different relaxation energies of the cubic lattice. For almost all of the compositions, the F_2g_ peak experienced a shift toward lower wavenumbers (red shift) compared to that of pure ceria, except for Zr–Ce–O, which experienced a shift toward a higher wavenumber (blue shift) (see Table 1). This behavior has been previously reported with similar compositions and is attributed to the distortion caused by zirconium ions present in the ceria lattice.^27^

The broad band located in the 540–600 cm^–1^ (Figure 2B) range has contributions from the oxygen vacancies formed,^28^ as well as the phase heterogeneity originating from the heteroatom (MO_*x*_, where M: heterocation) as a result of the lattice deformation/strain.^27^ It has been reported^3^ that by introducing LVD in the ceria lattice leads to oxygen vacancy formation to compensate for charge difference (i.e., Ce^3+^). The Raman band at 600 cm^–1^ is linked to the heteroatom/dopant and has nothing to do with the oxygen vacancies, and the bands at 540 cm^–1^ and below 400 cm^–1^ (e.g., 260 cm^–1^) are both linked to O_v_ formation. The latter has a coordination sphere of four nearest-neighbor (NN) metals (M_4_O_v_ entity) with 12 degrees of freedom following T_d_ symmetry. At the same time, O_v_ has 6 O^2–^ anions as next-nearest-neighbors (NNN), forming an O_6_O_v_ entity with 18 degrees of freedom satisfying an O_h_ symmetry. In the case of a biphasic system (co-presence of impurity dopant oxide and solid solution), the Raman band at 600 cm^–1^ corresponds to MO_8_ or MO_4_ dopant complexes that are formed depending on the type of the dopant. The types of defects can be intrinsic and extrinsic. For instance, in pure ceria, intrinsic defects are found due to the presence of Ce^3+^. Upon introduction of heteroatoms, extrinsic defects are formed, the band position of which depends on the dopant type, as can be seen in Figures 2B and S5. The strong interaction between ceria and the heteroatom induces microstructural changes, whereas the exposed planes of the nanoparticles contribute to the band position as discussed later.

The O_v_/F_2g_ ratio can therefore be used as a descriptor for the population of oxygen vacant sites in ceria-based solid solutions.^24^ Variations in the O_v_/F_2g_ ratio are observed due to the different chemical natures of heteroatoms used (Table 1 and Figure 2C). The highest O_v_/F_2g_ ratio is obtained for Ni–Ce–O, that is, 0.055, followed by Cu–Ce–O (i.e., 0.05). It is noticed that Zn–Ce–O presents the lowest O_v_/F_2g_ ratio value (i.e., 0.012) among the studied compositions. This finding is in agreement with a previous study on ZnO–CeO_2_, where Zn addition in ceria does not generate additional oxygen vacancies.^29^ It was reported by Laguna et al.^29^ that Zn-doped ceria has a similar value of O_v_/F_2g_ with pristine ceria, reflecting the low interaction of Zn^2+^ with CeO_2_ (segregated oxides mechanism of interaction). The formation of an increased concentration of oxygen vacancy defects is crucial in catalytic performance improvement, as will be discussed in a following section.

In the case of Fe–Ce–O, the F_2g_ band shows broadening and a shift toward lower wavenumbers (Figure 2A,C), in agreement with Laguna et al.,^30^ who reported that broadening and shifting in Raman spectra occur for Fe loading above 10 at. % in ceria. The modification of the shape and intensity induced by iron doping of ceria confirms the interaction between the host and the guest. The shift toward lower wavenumbers is related to the decrease in the crystallite size (confirmed by XRD), while the isomorphic substitution of Ce^4+^ with a cation of lower charge is associated with the shift toward higher wavenumbers.^30^ However, in the case presented in this work, crystallite size has the predominant role in causing the shift observed in the F_2g_ Raman band. The O_v_/F_2g_ ratio is found to be relatively low for Fe–Ce–O, that is, 0.025 (Table 1 and Figure 2C). This can be attributed to the fact that Fe^3+^ could be placed at interstitial and substitutional positions in the ceria lattice. Previous work on Ce_1–*x*_Fe_*x*_O_2−δ_ reported low concentrations of oxygen vacancies, suggesting an interstitial diffusion charge compensation mechanism of Fe^3+^ cations in the fluorite structure of ceria.^30^ The dopant interstitial compensation mechanism is well known for eliminating oxygen vacancies.^30^ It was reported^30^ that small amounts of Fe^3+^ doping (i.e., 10 at. %) contribute to the formation of oxygen vacancies, whereas larger amounts (i.e., 40 at. %) annihilate them for the case of Ce_1–*x*_Fe_*x*_O_2−δ_ prepared by the coprecipitation method. The F_2g_ band shift to lower frequencies along with the simultaneous increase in FWHM_F_2g__ (Figure 2D) is associated with oxygen vacancies generated upon doping and the size factor, the latter being reflected in the change in the lattice parameter upon doping (Figure 2C). The variation of the TM dopant induces a different primary crystallite size as this was calculated based on powder XRD (*D*_XRD_) and TEM (*D*_TEM_) studies. Usually, a smaller crystallite size (e.g., Fe and Mn) is accompanied by larger F_2g_ displacement (compared to the 465 cm^–1^ position). The FWHM trend among the dopants is inverse to the trend of the crystallite size, in agreement with the literature^31^ (Figure 2D).

#### 3.1.1 Critical Overview on the Homogeneity of the Structure Formed

Many studies in the literature report on the use of transition and rare earth elements as ceria dopants (see Tables S1 and S2). The content of the dopant element reported in literature studies is in the range of 5–30 at. %, yet no dopant metal oxide is reported as the copresent phase along with the ceria solid solution. A plethora of reported studies in the open literature support the formation of cubic homogeneous solid solution. However, cautiousness should be practiced as there is a small window where the dopant metal oxide can be highly amorphous or well dispersed, escaping the powder XRD detection limit. According to Reddy,^32^ criteria such as the XRD peak shift, lattice parameter change, and absence of diffraction peaks of the dopant metal oxide can be used to evaluate the formation of ceria homogeneous solid solutions. Among the literature reviewed, only Mn as the dopant element has the tendency to form the Mn_3_O_4_ phase, but this was found to take place only when calcination temperature is significantly larger than 500 °C (e.g., 800 °C).^32^ In the herein study, regarding the formation of a homogeneous solid solution (complete integration of the dopant element into the ceria lattice), the following remarks can be made: (a) the solubility limit of the dopant metal oxides into the ceria lattice is different (dependent on the nature of the dopant), although this limit can be further enhanced when the elastic energy required to introduce a guest into a host structure is low. The difference in these values (as shown in the Supporting Information, Section S2.1.3) reflects the variety in the extent of solubility limit expansion that can be achieved, which follows the order Ni > Co ∼ Mn > Zn > Fe > Cu. (b) A thorough examination of the powder XRD patterns and Raman spectra rather than the STEM, due to the larger sampling region size in the former techniques, was performed. The closer look up of the XRD patterns of all the catalysts (see Figures 1 and S2 and S3) along with the extracted parameters (e.g., peak position and lattice parameter) allows the following remarks: (b1) change in the lattice parameter values upon doping is due to the different ionic radii of the heteroatoms compared to Ce^4+^ (e.g., Fe^3+^: 0.64 Å, Mn^3+^; 0.65 Å vs Ce^4+^: 0.97 Å). (b2) No heteroatom metal oxide phase was identified in the case of Cu and Zr. In contrast, a closer examination of the XRD patterns showed that in the case of Zn, Ni, Fe, Mn, and Co, an impurity phase is formed (see Figures S2 and S3). The diffraction facets corresponding to the heteroatom metal oxides are shown in Figures S2 and S3. A thorough analysis of the Raman spectra also confirmed the presence of the heteroatom metal oxides, as the impurity phase, for the case of Zn, Ni, Fe, Mn, and Co (see Table S2, and Figure S5).

#### 3.1.2 Textural Studies

Variations in the BET surface area and specific pore volume are observed due to heteroatom-type effects (Table 1 and Figure S1C). BET area values for the mixed metal oxides varied in the 12–63 m^2^/g range. The BET area of ceria (14.8 m^2^/g) is enhanced after doping with particular TMs (i.e., Mn, Fe, and Cu), whereas no improvements are observed with the other TM heteroatoms. It has been already reported^33^ that the addition of different dopants, such as Nb, Zr, or Pr, to CeO_2_ leads to a higher surface area. In some cases, small changes are induced in the surface area (SSA, m^2^/g) due to the presence of the dopant. The dopant’s role is to increase the thermal stability of CeO_2_, thus hindering particle agglomeration.^33^ In another study, the BET surface areas of the metal-substituted ceria microspheres were very similar to that of pure ceria in the 10–20 m^2^/g range, driving us to the safe conclusion that the type of the dopant is not the only reason but also the synthesis method that contributes to the evolution of high specific surface areas.^34^ Also, depending on the chemical nature of the dopant, different nucleation and growth kinetics are expected, thus leading to different crystallite sizes, as shown in Table 1. The different crystallite sizes lead to different BET surface areas (m^2^/g). Furthermore, it has been reported by Jampaiah et al.^35^ that energetic factors on the surface due to microstrain phenomena (expansion/contraction) can lead to a smaller crystallite size in the case of doped-ceria catalysts than pristine ceria.

### 3.2 HRTEM–EDS Versus STEM-EDS Studies: Role of the M/Ce Ratio on Evaluating the Doping Efficiency

**Figure 3:**
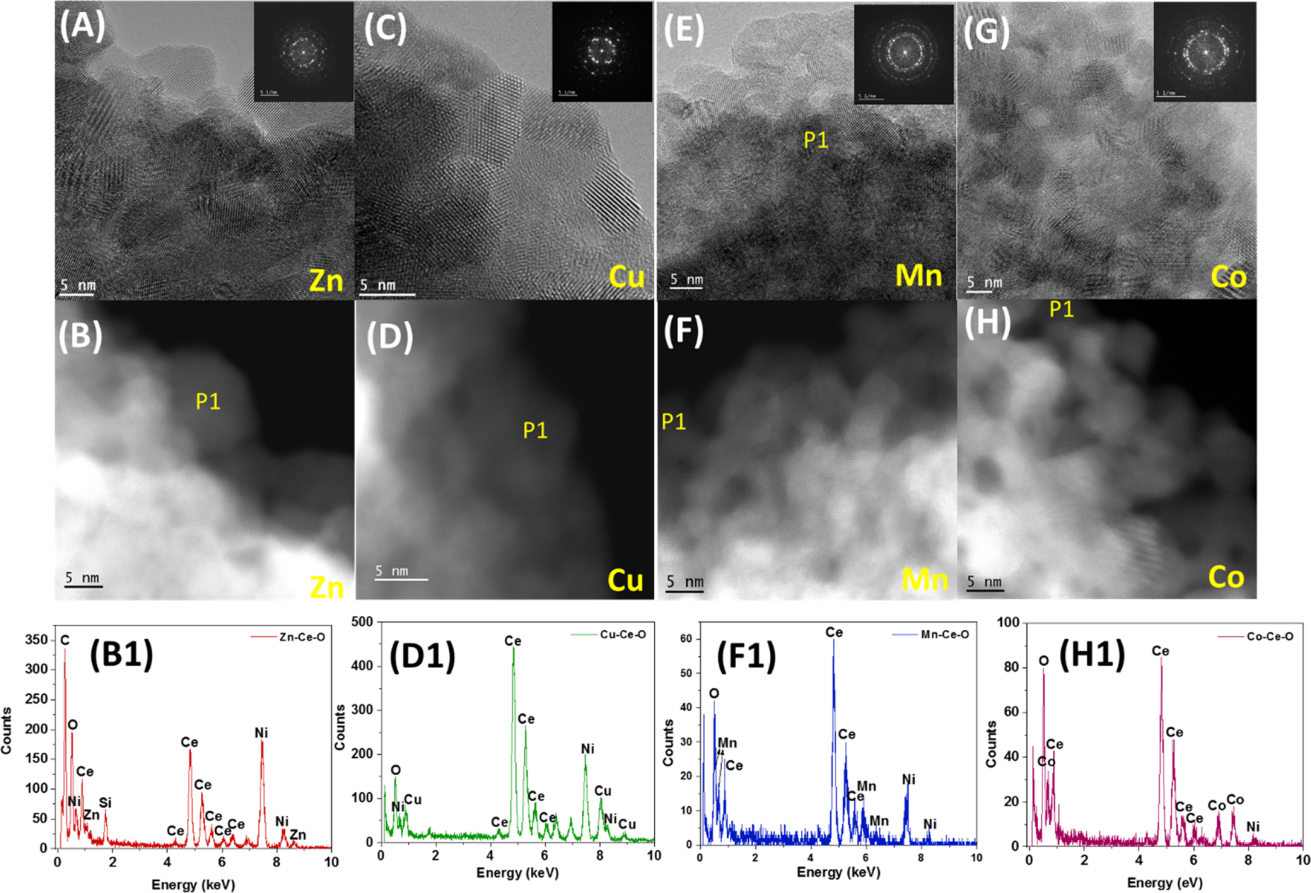
Zn−Ce−O catalyst. (A) HRTEM and (B) STEM-HAADF image with Z-contrast; (B1) EDS spectra of area marked in (B) as P1. Cu−Ce−O catalyst. (C) HRTEM and (D) STEM-HAADF image; (D1) EDS spectra of area marked in (D) as P1. Mn−Ce−O catalyst. (E) HRTEM and (F) STEM-HAADF image; (F1) EDS spectra of area marked in (F) as P1. Co−Ce−O catalyst. (G) HRTEM and (H) STEM-HAADF image; (H1) EDS spectra of area marked in (H) as P1.

Additionally, HRTEM studies (Figure 3) were performed to further study the shape, structure, and crystallite size; HRTEM revealed the rich crystallinity of the studied catalysts due to the abundance of lattice fringes observed. In addition, STEM-HAADF/EDX analysis was performed and the localized composition of Ce and TM (Mn, Co, Zn, and Cu) was evaluated in the same particle. The particle size based on the HRTEM images (*D*_TEM_) was calculated for Cu–Ce–O, Zn–Ce–O, Mn–Ce–O, and Co–Ce–O catalysts, and it was found to be in the 7–12, 8–9, 5–9, and 6–8 nm range, respectively, in agreement with the W–H method of estimation reported in Table 1. HRTEM and STEM-HAADF studies confirm the absence of any dopant oxide. It is also important to notice that the shape of all the catalysts was the same with no effect of the TM added into the morphology, as shown in the HRTEM images. The interplanar distances of the lattice fringes (or *d*-spacing) corresponding to {111} crystalline planes of ceria for the cases of M–Ce–O catalysts were found to be 0.310 ± 0.07 nm. These determined *d*-spacings are equal to the same planes of undoped ceria. It thus can be inferred with a high degree of confidence that the above-provided evidence supports the incorporation of the metals into the ceria lattice.

The EDX data acquired in the TEM mode enabled determining the elemental composition (at. %) of M–Ce–O samples on a larger scale, and the M/Ce composition ratios were found to be about 0.15, 0.16, 0.20, and 0.25 for Cu, Zn, Mn, and Co, respectively. These ratios are close to the nominal ratio of 0.25 for the cases of Co and Mn, whereas it was low for the cases of both Cu and Zn. The acquired results agree with solubility trends of these metals into the ceria solvent. The elemental composition of the samples at the level of individual ceria nanoparticles was also determined by acquiring the EDX data from one nanoparticle at a time in the HAADF-STEM mode of the microscope. The determined M/Ce composition ratios were present in 0.12, 0.08, 0.08, and 0.22 ranges for Cu, Zn, Mn, and Co, respectively. These numbers indicate that the elemental composition of dopants in individual nanoparticle of ceria is less than their composition at a larger scale. Therefore, for a given dopant, the difference between the M/Ce ratio in the TEM mode and the M/Ce ratio in the STEM mode indicates that possibly some of the heteroatoms exist in the samples as separate phases of their own oxides. However, this difference was negligible for the cases of Co and Cu, which implies that these metals got completely doped into ceria nanoparticles, whereas this was not the case for both Mn and Zn. Therefore, it can be concluded that the synthesized samples may have a fraction of their separate oxides along with doped ceria. In fact, the phase analysis of the samples using XRD (Figures 1A,B, S2 and S3) and Raman (Figure S5) seems to corroborate the findings of their compositional analysis using EDX.

### 3.3 X-ray Photoelectron Spectroscopy Studies

XPS has been employed to obtain information about the valence and oxidation state(s) of the elements and surface composition of Ce_0.8_M_0.2_O_2−δ_ mixed metal oxides (M = Cu, Co, Fe, Mn, Ni, and Zn). All mixed metal oxides showed similar Ce 3d spectra to that of pure ceria, as illustrated in Figure S6A. It has to be mentioned that X-ray source power values used are similar to those used in the other studies mentioned; hence, there is no reason to expect partial reduction of Ce^4+^ to Ce^3+^ in our analysis.^36^

In accordance with previous XPS work on cerium oxide,^36^ the labels u and v collectively correspond to the Ce 3d_3/2_ and Ce 3d_5/2_ ionization levels, respectively. The peaks labeled v, v″, v‴, u, u″, and u‴ correspond to Ce^4+^, whereas the v′ and u′ peaks correspond to Ce^3+^. The +4 valence state XPS peaks are predominant in the spectra, with little evidence for the +3 cerium oxidation state, indicative of almost a fully oxidized CeO_2_. This result is consistent with the powder XRD and Raman results presented and discussed above. No distinctive effects of the heteroatom type on the shape or intensity of XPS Ce 3d spectra are observed for any of the binary mixed metal oxide systems.

**Figure 4:**
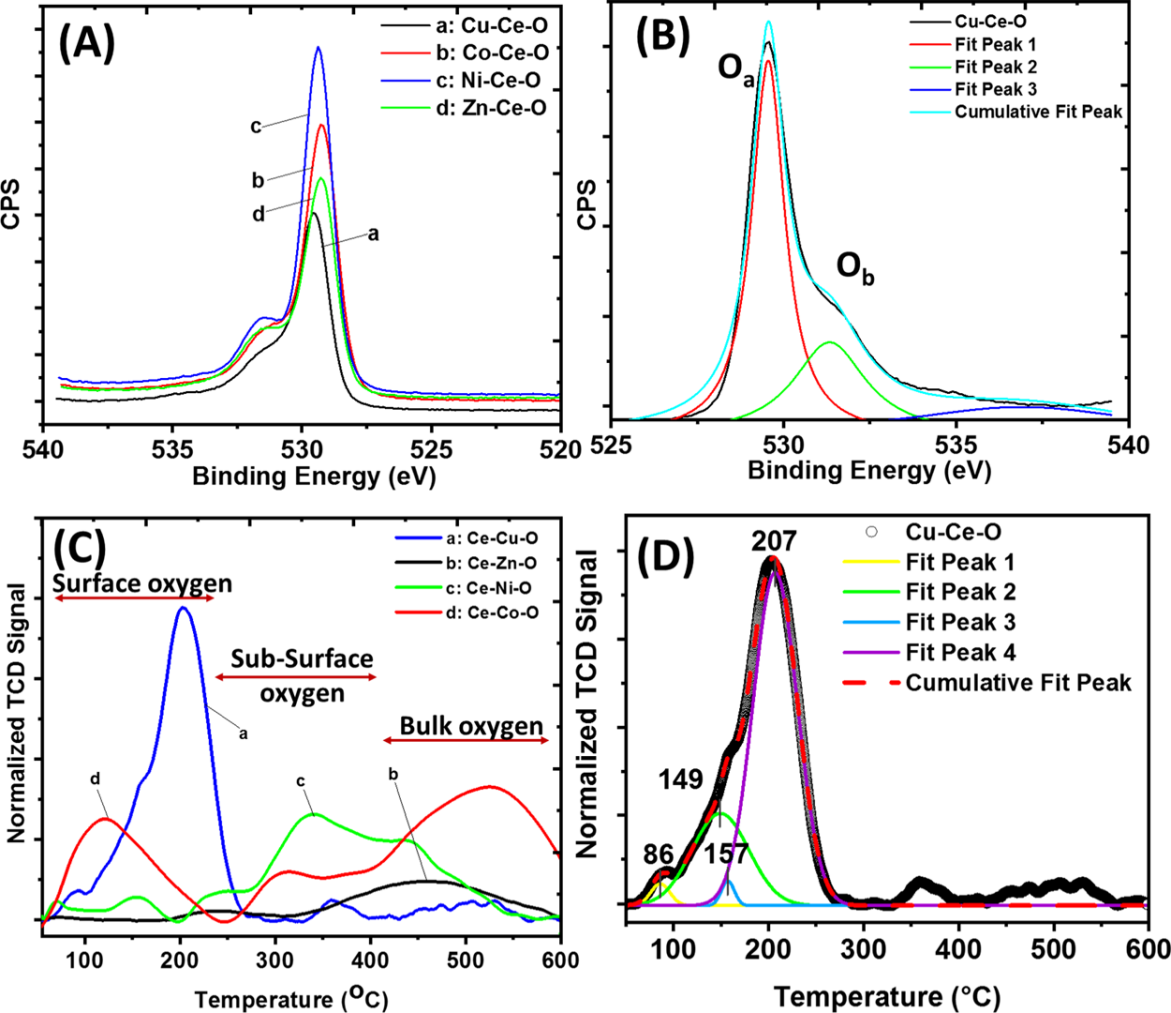
(A) O 1s XPS high-resolution spectra for pure CeO_2_ and M–Ce–O (M = Co, Cu, Ni, and Zn) solids. (B) Deconvoluted O 1s spectrum for the Cu–Ce–O solid. (C) H_2_-TPR traces of the different M–Ce–O solids and (D) deconvoluted H_2_-TPR profile of the Cu–Ce–O solid.

The O 1s XPS spectra for CeO_2_ and M–Ce–O are depicted in Figure 4A, where two distinctive peaks are observed at binding energies of ∼529 and ∼531.5 eV after deconvolution (Figure 4B, Cu–Ce–O). The O 1s peak width increases after introduction of the TM heteroatom to the ceria lattice, due to the variation in the chemical environment around the O atoms. The lower-binding-energy XPS peak corresponds to the lattice oxygen. On the other hand, there are a number of possible contributions to the higher energy XPS peak, namely, surface hydroxyl or carbonate species, resulting from atmospheric exposure of the sample and oxygen anions located close to oxygen vacant sites.^36^ Comparing the binding energy (*E*_b_) value of the most intense O 1s peak, which corresponds to the lattice oxygen, it was found that Zn-doped ceria (529.30 eV) presents almost the same value as that of pure CeO_2_ (529.20 eV), supporting the low interaction between the Zn–O and Ce–O entities.

In the case of Cu-, Fe-, and Mn-doped ceria, the *E*_b_ related to the O 1s is 529.55 eV (ΔBE = 0.35 eV), 529.53 eV (ΔBE = 0.33 eV), and 529.44 eV (ΔBE = 0.24 eV), respectively, followed by the Ni- and Co-doped systems, where the *E*_b_ is 529.33 eV (ΔBE = 0.13 eV) and 529.25 eV (ΔBE = 0.05 eV). It seems that the degree of TM–ceria interaction leads to the formation of different adsorbed oxygen species, such as superoxide (O^2–^) and peroxide (O_2_^2–^) upon oxygen activation, as shown by the O_b_ peak of the O 1s spectra (Figure 4B). C 1s peaks were observed for all samples, with a strong peak at a binding energy of ∼285 eV, corresponding to adventitious carbon, and a small peak at ∼289 eV associated with carbonate/carboxyl adsorbed species.

Core-level spectra for each TM heteroatom are shown in Figure S6B–F. Information related to their oxidation state is given in Table S3. The oxidation states of the various TM heteroatoms were found as follows: Cu^2+^, Co^2+^, Ni^2+^, Fe^3+^, Mn^2+^, and Zn^2+^ (details can be found in the Supporting Information).

#### 3.3.1 Heteroatom Type Effect on the Ce Valence

With regard to how the heteroatom element changes the valence of Ce, Paparazzo^37^ has described in detail that reliable peak fitting of the Ce 3d peaks to obtain the Ce^3+^/Ce^4+^ ratio is not straightforward and can often result in incorrect Ce^3+^/Ce^4+^ values. We have considered two different methods of determining the Ce^3+^/Ce^4+^ ratio. The first one involves the use of the strong Ce^4+^ u‴ peak at ≈916 eV and comparison of this to the total Ce 3d intensity, as described by Henderson et al.^36^ This method gives the following order for the highest to lowest Ce^3+^ content: Zn > Ni > Co > Fe > Cu > Mn.

In Figure S7, the Ce region between 875 and 895 eV has been plotted, in a similar manner to that given in the paper by Henderson et al.^36^ The peaks in this region are the v^0^, v, v′, and v″ peaks, where the v and v″ peaks (see Figure S6A) correspond to Ce^4+^ and v^0^ and v′ to Ce^3+^ species. A peak fit for the doped-ceria samples yields the following order, for the highest to lowest Ce^3+^ content: Fe > Cu > Co > Zn > Mn > Ni. Thus, there is a discrepancy between the Ce^3+^/Ce^4+^ ratio order for the two different methods employed, and none shows a good correlation with the corresponding trend of Cu > Co > Ni > Mn > Fe > Zn observed for the catalytic performance and DFT predictions. However, this could be due to similar peak shapes exhibited by all the spectra (indicative of very similar Ce^3+^/Ce^4+^ ratios), the complexity of the peak fit, and the influence of atmospheric exposure.

#### 3.3.2 Oxygen Environment

Returning to the Ce 3d peak plot in Figure S7, all samples, undoped and doped ceria, exhibit very weak v′ peak intensities, indicative of low Ce^3+^/Ce^4+^ ratios. Comparing these spectra with those from Henderson et al.^36^ gives a stoichiometry of all samples >CeO_1.95_, corresponding to a Ce^3+^ content of <10% for all samples. The presence of Ce^3+^ species in the undoped sample may seem surprising, but exposure to the atmosphere seems to have promoted the formation of a small amount of hydroxide, as usually observed on most metal oxide surfaces, due to the interaction with surface-adsorbed water. In a thermodynamic study reported by Bravo-Suárez et al.,^38^ there is a relatively strong thermodynamic driving force (−59.4 kJ/mol) in terms of free energy of hydration for Ce_2_O_3_ to form Ce(OH)_3_. No data were given for CeO_2_, presumably because Ce^4+^ oxide does not lead to a hydroxide phase. Thus, the presence of Ce^3+^ (oxygen vacancies) on the surface of this undoped CeO_2_ sample promotes Ce(OH)_3_ formation. This can be seen from the O 1s spectra, where the undoped sample gives a clear peak at 531.1–531.4 eV, which has been attributed to atmospheric contamination (OH^–^/CO_3_^2–^) and/or oxygen vacancies, and a small adsorbed water component at ≈533 eV. It is not possible to determine the relative contributions associated with the 531.1–531.4 eV peak, but OH^–^ is most probably the dominant contributor to this peak. The doped samples all show a larger intensity of the high-binding-energy O species compared to CeO_2_.

Interestingly, if the high-binding-energy O 1s peak corresponding to OH^–^/CO_3_^2–^/oxygen vacancies at 531.1–531.4 eV is considered, and compared to the lattice oxygen peak, then the (“contamination/defect O”)/(lattice O) peak intensity ratio results in the following order Cu > Fe > Co > Zn > Ni > Mn > CeO_2_. Considering that the thermodynamic driving force for the formation of heteroatom species hydroxides [Cu(OH)_2_, Ni(OH)_2_, Mn(OH)_2_, Co(OH)_2_, Zn(OH)_2_, and Fe(OH)_2_] is lower than that of Ce(OH)_3_, then the increase in oxygen vacancies resulting from doping will cause an increase in Ce(OH)_3_ formed on the surface. Then, if the hydrocarbon contamination is also considered, the (“total overlayer + O vacancies”)/(lattice O) peak intensity ratio results in the following order Cu ≈ Fe > Co > Ni > Mn > Zn > CeO_2_. The rationale for examining the total overlayer intensities is that these potentially give a crude measure of relative surface energy for the different doped ceria surfaces, with a greater concentration of adsorbed species and hydroxide representing a greater surface energy. Zhuang et al.^39^ have noted that surface energy generally results in higher chemical reactivities and that surface energy can also be directly correlated with catalytic activity. In this respect, Zhuang et al.^39^ found that there is an optimum surface energy, where a value above and below this optimum corresponds to lower catalytic activity. This was correlated with Sabatier’s principle, that is, those species which show an interaction, which is too strong or too weak with reactive intermediates, will show a lower catalytic performance. The catalytic performance of the doped ceria surfaces shows the following trend: Cu > Co > Ni > Mn > Fe > Zn, which corresponds reasonably well with the rankings given for the relative overlayer + oxygen vacancy intensities (except for the Fe-doped CeO_2_), and substantially better than the two methods for the determination of the Ce^3+^/Ce^4+^ ratio, which indicates that Zn-doped CeO_2_ would be expected to give a high rather than a low catalytic performance.

To summarize, determination of the bulk Ce^3+^/Ce^4+^ ratio from XPS spectra where the sample has been exposed to the atmosphere is unreliable, due to interaction with water vapor to form an outer Ce(OH)_3_ layer on both doped and undoped CeO_2_ samples. However, quantification of the concentration of overlayer species (predominantly surface hydroxide and adsorbed hydrocarbon) on the doped and undoped CeO_2_ catalysts provides a crude measure of surface energy and yields a generally good correlation with catalytic activity.

#### 3.3.3 Critique on the Peak Fitting of the Heteroatoms

The peak fitting of the heteroatom 2p spectra was cautiously avoided since there is significant variation between the binding energies of the same compound provided in the databases, all of which are considered to be reliable. Furthermore, there is no information in the NIST database on the fine structure of TM core-level 2p peaks, which is required for a peak fit to be attempted/performed. The main issue with peak fitting of the TM 2p peaks is that for these elements, the photoelectron peak is complex due to the presence of the fine structure for one or more of the following reasons: (a) plasmon features; (b) shake-up satellites; and (c) multiplet splitting, all of which vary with the oxidation state.^40^

### 3.4 Catalytic CO Oxidation Performance of Doped CeO_2_

**Figure 5:**
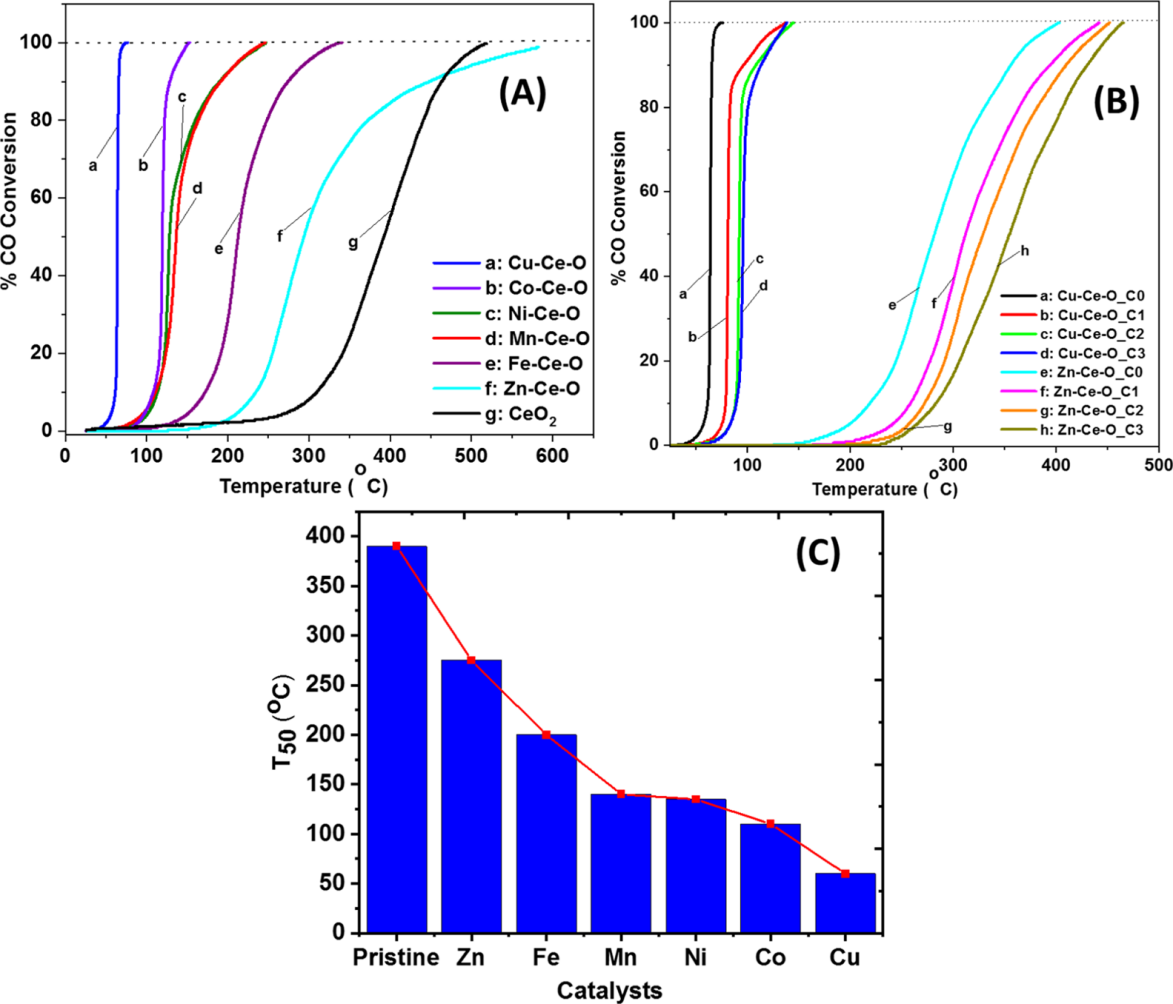
Light-off curves of CO oxidation reaction obtained over the different TM-doped CeO_2_ catalysts using 4 vol % CO/20 vol % O_2_/He (A) and 4 vol %CO/*y* vol % CO_2_/20 vol % O_2_/He (B) feed gas compositions; Ci: C1 (1%), C2 (2%), C3 (4%), WHSV = 60,000 cm^3^ g^–1^ h^–1^. (C) Comparative results of *T*_50_ (°C) conversion temperature for all the herein TM-doped CeO_2_ catalysts.

The effect of the chemical nature of the TM heteroatom on the CO oxidation activity in terms of CO conversion (%) of doped CeO_2_ is presented in Figure 5A. The CO oxidation activity (CO/O_2_/He) is significantly improved after heteroatom introduction in the ceria lattice for all the doped-CeO_2_ mixed metal oxides. However, specific heteroatoms outperformed others. In the absence of CO_2_ in the feed (Figure 5A), the activity follows the order Cu > Co > Mn ∼ Ni > Fe > Zn > CeO_2_ (Figure 5C). To justify this activity order, the following considerations are noted:

(a) Exceptional performance was exhibited by Cu–Ce–O with *T*_50_ ∼ 60 °C and ∼100% CO conversion at ∼65 °C (Figure 5A). The high activity of Cu–Ce–O is attributed to the synergistic interaction between ceria and Cu^2+^ through partial solid solution formation (XRD and Raman studies) that enhanced oxygen mobility and concentration of oxygen vacancies, as indicated in Figure 2C (among the highest O_v_/F_2g_ ratio). The temperature for 50% CO conversion *T*_50_ (°C) coincides with the low reduction temperatures as indicated in the H_2_-TPR studies (*T*_max_ = 100 °C, Figures 4C,D and S8 and S9) and the CO_2_ desorption at relatively low temperatures obtained in the CO_2_-TPD studies (*T*_max_ = 100 °C, Figure S8C). It is important to recall here that according to the MvK mechanism, CO_2_ formation by the participation of lattice oxygen is accompanied by the creation of an oxygen vacant site. The present isotopic studies (see the following Section 3.5) showed that O_lattice_ largely participates in the CO oxidation reaction over Cu–Ce–O. (a1) Comparing the nominal M/Ce ratio (0.25) with the ones estimated based on TEM–EDX and STEM-HAADF-EDX, it is obvious that for the Cu, Zn, Mn, and Co, different amounts of the heteroatom preferred to form the impurity oxide phase. For the cases of Zn, Mn, and Co heteroatoms, confirmation of the impurity oxide is provided (see XRD and Raman in Figures S2, S3 and S5), while for the Cu, failure to detect the impurity phase through XRD and Raman suggests the high dispersion of the CuO phase; the latter can also be a reason for the best catalytic performance of the Cu–Ce–O solid.
(b) The second best-performing doped-CeO_2_ catalyst is Co–Ce–O with *T*_50_ ∼ 110 °C and ∼100% CO-conversion at ∼150 °C, a temperature range that coincides with its reduction profile (see TPR studies, Figure 4C). This is attributed to the close interaction between active Co^2+^ and ceria species that enhances the redox cycles of Ce^3+^/Ce^4+^ and Co^2+^/Co^3+^. Also, the TEM–EDX along with STEM–EDX studies showed that the composition of Co–Ce–O in large and short ranges is very close to the nominal (0.25), which implies that Co was the best incorporated heteroatom among the investigated ones. Only minor amounts of impurity oxide can be proposed based on our findings.
(c) Mn–Ce–O and Ni–Ce–O demonstrated similar CO oxidation activity behavior with *T*_50_ at ∼140 °C and achieving ∼100% CO conversion at ∼230 °C. This behavior is due to the Mn^4+^ and Ni^2+^ species having similar reducibility behavior under CO oxidation reaction conditions.
(d) The worst-performing catalysts appear to be the Fe–Ce–O and Zn–Ce–O with *T*_50_ values of 200 and 270 °C, respectively. The low activity exhibited by these two specific mixed metal oxides is due to the low reducibility of the respective surface metal cations at low temperatures (H_2_-TPR, Figure 4C), which is strongly related to the low mobility of lattice oxygen at relatively low temperatures. This can be attributed to the following reasons:
  (d1) In the case of the Zn heteroatom, according to the XRD studies, ZnO phase impurity was identified, introducing ZnO/Zn–Ce–O phase (grain) boundaries for oxygen diffusion.
  (d2) A low tendency of the valence state of Zn^2+^ species to be reduced in the presence of CO, thus leading to low oxygen mobility in the ceria lattice.
  (d3) In the case of the Fe heteroatom, not all Fe added participated in the formation of substitutional solid solution, resulting in Fe at interstitial sites, according to the present Raman results.^30,41^ High temperatures are thereby necessary to engage the diffusion of bulk oxygen onto the surface of zinc- and iron-doped ceria for CO oxidation. Thus, at a temperature of 250 °C at which the oxygen mobility was studied (see Section 3.4), O_lattice_ participation was found to a small extent only in the case of Zn–Ce–O.
  (d4) CO_2_-TPD traces for Zn–Ce–O and Fe–Ce–O (Figure S8C–E) showed that CO_2_ is released at temperatures as high as their *T*_50_. This shows their preference to hold CO_2_ at low temperatures and then release it at higher temperatures, thus blocking the regeneration of O_v_ and reducing the population of surface labile lattice oxygen species that take place in the CO oxidation reaction.
(e) Strain and catalytic activity: It is well established in the literature that lattice strain affects the catalytic activity for a wide spectrum of reactions spanning from catalysis to electrocatalysis. Based on these studies, it is obvious that lattice strain can be used as the catalyst design criterion. In these studies,^42^ it is demonstrated that in ceria-related materials, either in thin films or heterostructures, strain can be used to tailor the material properties. In particular, it was shown that tensile strain, which is the case of all the herein solids, leads to isolated surface vacancies with next-nearest-neighbor (NNN) polarons, whereas in the case of compressive strain, isolated vacancies are preferably located on the subsurface having nearest-neighbor (NN) polarons. Additionally, dimers on both the surface and subsurface are formed when isolated species exist under compressive strain. Therefore, strain can act as a manipulator for the vacancy cluster formation. In the same study, an almost linear dependence of the surface strain and oxygen vacancy formation was found. In this study, the tensile strain order was found to be Mn > Fe > Cu > Ni > Zn > Co, whereas the O_v_ population follows the Ni > Cu > Mn > Co > Fe order. The discrepancies between the two series can be explained based on the doping efficiency (TEM–EDX studies) and phase heterogeneity (XRD, Raman). It was demonstrated that in the case of the Pt NP,^43^ site-specific strain was found to boost the TOF of the reaction and compressive strain lowers the CO adsorption energy (lower light-off temperature) since under these conditions, CO poisoning phenomena are reduced, whereas expansion leads to a CO binding energy increase. Higher lattice strain causes higher mobility of lattice oxygen, which enhances the oxidation reaction via the Mars and van Krevelen mechanism.^44^
(f) Oxygen vacancy (O_v_) and catalytic activity: the oxygen vacant sites, O_v_, play a crucial role in the CO/O_2_ reaction by activating the O_2_ molecular species approaching the surface from the gas phase. During the CO oxidation reaction, CO_2_ is formed; the latter being chemisorbed, dissociated onto the vacant sites, or both (a process boosted by the ceria basicity as well). CO_2_ chemisorption onto the O_v_ leads to the drop of the available O_v_ population for O_2_ activation. With regard to the O_v_/F_2g_ ratio dependence with the dopant, it has to be noted that CO_2_ chemisorption onto ceria is structure-specific and charge transfer occurs from the reduced ceria to the CO_2_ molecule, leading to the formation of unidentate carbonate species.^45^ An important role in the CO_2_ chemisorption is also played by the structure of the O_v_ (e.g., in plane vs split vacancies; with the latter being more energetically favorable for the CO_2_ chemisorption). Based on the abovementioned discussion, the net concentration of oxygen-vacant sites is a trade-off between the rates of the two previously mentioned processes, O_2_ activation and CO_2_ chemisorption, both taking place at a competitive manner. It has also been mentioned that the difference in the redox potential for the dopants compared to that of Ce^4+^/Ce^3+^ is a drive for the redox properties of the doped ceria. In the case of Ni and Cu dopants, Cu^2+^ → Cu^0^ (0.340 V) and Ni^2+^ → Ni^0^ (0.257 V) compared to Ce^4+^/Ce^3+^ (1.61 eV).^46^ Therefore, a higher drive for the formation of O_v_ over the Ni–Ce–O catalyst can be anticipated.
(g) Porosity and catalytic activity: From the catalysis point of view, geometric factors (specific surface area and particle size) have crucial role for the reaction of interest over unsupported metal oxides. In this work, no control of the BET was intended through design of synthesis experiments. In such a case, someone would expect that it might be extremely hard to conclude if the CO oxidation activity boost is due to intrinsic/extrinsic effects of the dopant. Luckily, for the present TM-doped ceria systems, it can be safely said that a linear correlation between the catalytic activity and the energy of O_v_ formation (*E*_vf_) has been established.^42^ In general, an increase in specific surface area leads to an increase in defect site density (#sites/volume), and thus, more surface chemisorption sites are generated. The CO oxidation activity is proportional to the defect site concentration. In a more fundamental aspect, the increase in surface area can be correlated with the decrease in surface energy, and this has an apparent effect on the size and shape of the nanoparticles.^6,47^

#### 3.4.1 Effect of CO_2_ on the Feed Stream

The activity of the best- and worst-performing mixed metal oxides (i.e., Cu–Ce–O and Zn–Ce–O) was evaluated for the CO oxidation in the presence of different concentrations (*C*_*i*_) of CO_2_ [*C*_*i*_ = 1, 2, and 4 vol %, where (*i* = 1, 2, and 3)] in the feed stream, as shown in Figure 5B. The main observation is that the presence of CO_2_ in the feed gas stream reduces the *T*_50_ of both catalysts by 30–80 °C for all the CO_2_ concentrations used. This can be attributed to the fact that CO_2_ competes with O_2_ for adsorption sites, thus blocking surface active sites, such as oxygen vacancies, by restraining dissociation of O_2_.^48^ Also, CO_2_ can compete with CO for active surface lattice oxygen through the formation of carbonate-like species on the catalyst surface.^49^ The latter explains the downward shift of *T*_50_ by the CO_2_ blockage of less active surface lattice oxygen species. Regardless of the CO_2_ presence, the CO conversion profile remains similar for all the catalysts, suggesting only small influence of adsorbed CO_2_ on the kinetics of CO oxidation.

Regarding the role of CO_2_ in the feed stream, it is important to mention the following points: (a) the addition of CO_2_ (reaction product) would have an effect only if the CO oxidation reaction is considered reversible. This, however, is not true in the temperature range studied herein, where CO_2_ dissociation to form CO(g) and O_2_(g) is kinetically inhibited. (b) Additionally, ceria surface sites are basic sites, and thus, the CO_2_ in the feed is expected to get adsorbed before the CO_2_ product is formed after CO oxidation reaction (carbonate-mediated mechanism).

The reaction rate suppression due to the presence of CO_2_ could be linked with the chemisorption energetics of CO_2_ on ceria toward the formation of carboxylate and carbonate species. It has been suggested that the carboxylate/carbonate species lower the CO oxidation rate in a manifold way, namely, decreasing the available ceria surface for oxygen adsorption/activation, lowering the adsorbed oxygen amount on ceria, shrinking the interface reaction, and lowering the rate of surface oxygen diffusion.^50^

The abovementioned activity trends are in agreement with published studies. For example, Kim et al.^51^ tested, using DFT tools, 13 dopant elements from periods 4–6 in the periodic table, resulting into 91 binary combinations. Their study showed that Cu alone and Cu + Ag are the best dopant and double dopant, respectively, due to the easiness in oxygen vacancy, O_v_ formation. The same group has reported, in another study, that for the CO oxidation performed over Fe, Mn, and Mn–Fe codoped CeO_2_(111), there is a linear relationship between the energy for vacancy formation (*E*_vf_) and catalytic activity. Park et al.^52^ investigated different combinations of double-doped ceria systems, such as (Co, Cu)-, (Cu, Ni)-, and (Co, Ni)-doped CeO_2_ for CO oxidation. They found that Cu-doped CeO_2_ initially exhibits high activity, but during long-term activity testing, (Co, Cu)-doped CeO_2_ becomes more active than the Cu dopant alone, showing that the selection of (co)dopants is a crucial step.

In the study reported by Luo et al.^53^ for CO oxidation over Pd-doped MO_*x*_–CeO_2_ (M = Mn, Fe, Co, Ni, and Cu), the catalysts were prepared by the coprecipitation method. They found that the Ce–Cu–O catalyst was the most active, while Ce–Fe–O was the least active, with *T*_50_ of 62 and 179 °C, respectively. The other three catalysts, Ce–Mn–O, Ce–Co–O, and Ce–Ni–O, exhibited similar activities with *T*_50_ between 110 and 120 °C.

### 3.5 Transient ^18^O-Isotopic Studies—Probing the Participation of Lattice Oxygen in the CO Oxidation Reaction Path

A fundamental question of the CO oxidation reaction mechanism over metal oxide catalysts is whether and to what extent their lattice oxygen participates in the reaction, in addition to the gas-phase oxygen (O_2_) present in the feed gas stream. To provide solid conclusions about this important aspect of the catalytic CO oxidation mechanism, the mobility of doped-ceria lattice oxygen and its participation in the CO oxidation were studied under dynamic conditions. After ^16^O of the doped-CeO_2_ was partially replaced (subsurface) by ^18^O, a step-gas switch to CO/Ar or CO/O_2_/Ar was made and the ^18^O-labeled CO_2_’s were monitored with reaction time, and the corresponding transient rates were estimated via material balance.

#### 3.5.1 ^16^O/^18^O Isotopic Exchange (Mobility of O_lattice_)

**Figure 6:**
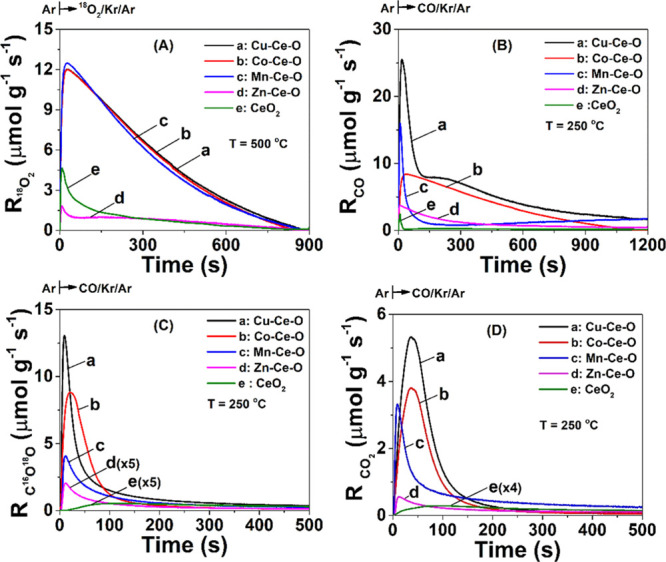
(A) Transient rates (μmol g^–1^ s^–1^) of ^18^O_2_ consumed during the step-gas switch Ar → 2 vol % ^18^O_2_/2 vol % Kr/Ar (15 min) performed at 500 °C on (a) Cu–Ce–O, (b) Co–Ce–O, (c) Mn–Ce–O, and (d) Zn–Ce–O doped ceria along with pristine CeO_2_ (e) solids. Transient rates (μmol g^–1^ s^–1^) of CO(g) consumed (B), C^16^O^18^O (C), and C^16^O_2_ (D) formed during the step-gas switch Ar → 4 vol % CO/2 vol % Kr/Ar (20 min) performed at 250 °C following oxygen exchange (A) over (a) Cu–Ce–O, (b) Co–Ce–O, (c) Mn–Ce–O, and (d) Zn–Ce–O doped-ceria solids, along with pristine CeO_2_ (e).

Figure 6A presents the transient rates (μmol g^–1^ s^–1^) of ^18^O_2_ consumed during the step-gas switch Ar → 2 vol % ^18^O_2_/2 vol % Kr/Ar (15 min) performed at 500 °C on the TM doped-CeO_2_ solids with the best and worst catalytic CO oxidation behavior. The 15 min gas treatment of the catalytic surface with the ^18^O_2_ isotope gas led to the partial exchange of ^16^O in the solid (surface and subsurface) for ^18^O, before the switch to the CO oxidation reaction feed gas stream. Madier et al.^54^ investigated the mechanism of oxygen exchange through ^16^O/^18^O transient isotopic experiments (TIE). Two possible mechanisms were proposed for the ^16^O/^18^O hetero-exchange, simple versus multiple, the latter being more dominant over Ce–Zr–O solids.^54^
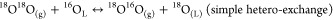
9
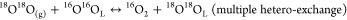
10

Similar ^16^O/^18^O studies have been performed over ceria with different morphologies, where the lattice oxygen reactivity was found to follow the order rods > cubes > octahedra.^55^

The transient rate response curves of ^18^O_2_ consumption appear to be very similar in terms of shape and position in time for the Cu–Ce–O, Mn–Ce–O, and Co–Ce–O mixed metal oxides except for the Zn–Ce–O, as shown in Figure 6A. The amount of ^18^O exchanged was estimated after integration of the R(^18^O_2_) transient rate versus time response curve, and this was found to be in the 8.43–8.85 mmol g^–1^ range for the Cu–Ce–O, Mn–Ce–O, and Co–Ce–O. In the case of Zn–Ce–O, this was found to be much smaller (1.57 mmol/g, Table 2). The latter result shows the inherent difficulty of the Zn–Ce–O lattice structure to exchange oxygen or its limited population of oxygen vacant sites, in agreement with the H_2_-TPR traces (Figure 4C) and the powder XRD (Figure 1A) results. The latter revealed that the structural characteristics of the Zn–Ce–O mixed metal oxide is that ZnO appears as a segregated oxidic phase, and as a result of this, not all Zn^2+^ cations were incorporated in the CeO_2_ lattice. Thus, less lattice distortion and weakening of the M–O chemical bonds occur.

**Table 2:** Amount of ^18^O Exchanged (mmol ^18^O g^–1^) at 500 °C over the TM-Doped Ceria along with the Pristine Ceria Solids Following the Step-Gas Switch Ar → 2 vol % ^18^O_2_/2 vol % Kr/Ar (15 min)^a^

catalyst	^18^O-exchanged (mmol g^–1^)	CO consumed (mmol g^–1^)	C^16^O_2_ (mmol g^–1^)	C^16^O^18^O (mmol g^–1^)	C^18^O_2_ (mmol g^–1^)
Cu–Ce–O	8.85 (88.5)^b^	6.3 (63)^d^	0.65 (6.5)^b^	1.75 (17.5)^b^	2.46 (24.6)^b^
Co–Ce–O	8.58 (116.0)^b^	4.1 (55.4)^d^	0.43 (5.8)^b^	1.15 (15.5)^b^	1.55 (20.9)^b^
Mn–Ce–O	8.43 (21.6)^b^	3.4 (8.7)^d^	0.35 (0.90)^b^	0.98 (2.5)^b^	0.55 (1.4)^b^
Zn–Ce–O	1.57^c^	0.6	0.21	0.11	0.02
CeO_2_	1.48 (16.1)^b^	0.28 (3.04)^d^	0.05 (0.5)^b^	0.07 (0.8)^b^	0.02 (0.3)^b^

a Amounts (mmol g^–1^) of CO consumed and C^16^O_2_, C^16^O^18^O, and C^18^O_2_ formed at 250 °C during the step-gas switch Ar → 4 vol % CO/1 vol % Kr/Ar (20 min) following ^16^O/^18^O exchange at 500 °C.

b Number in parentheses denotes the equivalent number of surface monolayers of lattice oxygen based on the theoretical surface oxygen density for ceria (6.2 μmol m^–2^). This estimation considers the BET area based on which the equivalent surface monolayer of ^16^O per gram basis is as follows: Cu–Ce–O = 0.1 mmol g^–1^, Co–Ce–O = 0.074 mmol g^–1^, Mn–Ce–O = 0.39 mmol g^–1^, and CeO_2_ = 0.09.

c Equivalent surface monolayers were not estimated, given the nonfluorite structure of the Zn–Ce–O solid material.

d Equivalent CO in surface coverages of lattice oxygen reacted off.

#### 3.5.2 Participation of O_lattice_ in the CO Oxidation Reaction Path

Figure 6B shows the transient rates (μmol g^–1^ s^–1^) of CO(g) consumption during the step-gas switch Ar → 4 vol % CO/2 vol % Kr/Ar (20 min) performed at 250 °C on the (a) Cu–Ce–O, (b) Co–Ce–O, (c) Mn–Ce–O, and (d) Zn–Ce–O binary mixed metal oxides, following the ^16^O/^18^O isotopic exchange described above (see Section 3.5.1, Figure 6A). The rapid increase in the rate of CO consumption, *R*_CO_, for all solids, the relatively fast decline of it for the Mn–Ce–O solid, but the slower decline for the Zn–Ce–O, Cu–Ce–O, and Co–Ce–O catalysts with different prolonged times are clearly seen. Of interest is the Cu–Ce–O catalyst, which presents clearly two rate maxima, strongly suggesting for a largely different kinetics of CO oxidation by lattice oxygen compared to the other three catalyst samples.

The sharp increase in the *R*_CO_ observed in all the mixed metal oxide catalysts at the switch to CO/Ar (Figure 6B) is due to the reaction of CO with a pool of active surface lattice oxygen species, while the slow decay with tailing following the sharp initial rate is also due to the reaction of CO with subsurface lattice oxygen. The latter reached the surface by diffusion from the bulk of the solid. This oxygen diffusion step controls the overall rate of CO oxidation with lattice oxygen. The amount of CO consumed for the Cu–Ce–O and Zn–Ce–O catalytic materials after 20 min in CO/Ar gas treatment was found to be the highest (6.3 mmol/g) and the lowest (0.6 mmol/g), respectively. This result agrees with the steady-state catalytic CO conversion rate data presented in Figure 5, as well as with the DFT studies to be presented in the following Section 3.5.

Further direct evidence for the participation of subsurface lattice oxygen in the oxidation of CO is provided by the transient rate of C^16^O^18^O formation (Figure 6C), integration of which results in an amount that exceeds one equivalent monolayer of surface lattice oxygen, as reported in Table 2. In the case of Cu–Ce–O (most active catalyst), an amount of 8.7 monolayers of lattice oxygen (^18^O) was estimated to be able to diffuse to the surface of the catalyst and react with CO(g) to give C^16^O^18^O(g). The latter amount is the largest one obtained for the series of doped-ceria catalysts, whereas Zn–Ce–O provides the least amount (0.79 monolayers, Table 2). Furthermore, the Cu–Ce–O catalyst shows ∼25 times larger initial rates of C^16^O^18^O formation than Zn–Ce–O (Figure 6C, first few seconds of the transient). Considering the fact that both catalysts had exchanged at least one surface monolayer of lattice oxygen before the gas-switch to CO/Ar (see Table 2) and this surface oxygen monolayer differs only by ∼1.5 times (Table 2), then the significantly larger initial rate of CO oxidation with ^18^O surface lattice oxygen is due to the larger intrinsic activity (*k*) of such surface lattice oxygen species present on the Cu–Ce–O surface compared to that on the Zn–Ce–O surface. As discussed above, this is due to the weaker M–O–M bonding established in the Cu–Ce–O than the Zn–Ce–O solid.

Figure 6D depicts the transient rates of C^16^O_2_ formation for the four doped-ceria catalysts obtained after the step-gas switch to the CO/Ar gas stream. It is clearly shown that these transient rates are lower than those of C^16^O^18^O(g) (Figure 6C) at any time during the transient, especially the initial rates (during the first few seconds after CO is introduced over the catalyst surface). Furthermore, the peak maximum of the C^16^O_2_ transient rate is shifted to larger reaction times compared to that of C^16^O^18^O(g), but the shape of the whole transient rate curve appears to remain practically the same. These important features of the transient rates of C^16^O^18^O (Figure 6C) and C^16^O_2_ (Figure 6D) are related to the kinetics of exchange of C^16^O^18^O with ^18^O and ^16^O lattice oxygen present in the solid according to the following elementary steps described in eqs 11–14
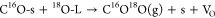
11
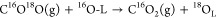
12
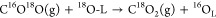
13
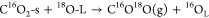
14

Equation 11 describes the basic elementary reaction step of CO oxidation via the participation of lattice oxygen. Since for all four TM ceria-doped catalysts, the first surface monolayer has been exchanged with ^18^O (see Table 2, Section 3.5.1), no C^16^O_2_ is expected at the step-gas switch to CO/Ar (first few seconds) as confirmed experimentally (Figure 6D). The C^16^O^18^O, however, can exchange one of its oxygen with a surface lattice oxygen, ^18^O-s or ^16^O-s, at a single elementary reaction step described in eqs. 12–14.

According to the results provided in Table 2 and the carbon material balance, the amount of CO consumed appears larger than the sum of all three possible isotopic CO_2_’s measured. This suggests that at 250 °C, a pool of strongly adsorbed CO_2_ in the form of carbonate-type species must be formed. The latter finds strong support by the CO_2_-TPD traces shown in Figure S3C,E, where a significant amount of CO_2_ desorbs at *T* > 250 °C.

#### 3.5.3 Site Activity of TM-Doped CeO_2_ Lattice Oxygen in CO Oxidation

The site activity, in terms of TOF (s^–1^) of lattice oxygen of the Cu-, Co-, Mn-, and Zn-doped ceria toward reaction with CO(g) was estimated and compared based on the measured initial rate (μmol g^–1^ s^–1^) of C^16^O^18^O formation at 250 °C (Figure 6C) and the surface concentration (μmol O_s_ g^–1^) of lattice oxygen for each solid, as reported in Table 2, via the following eq 15, and results are shown in Figure 6A.
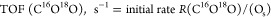
15

It is illustrated that Cu–Ce–O has the largest TOF value, whereas Zn–Ce–O has the lowest one. The Co–Ce–O exhibits slightly lower TOF than Cu–Ce–O, while Mn–Ce–O exhibits slightly higher TOF compared to Zn–Ce–O. This site activity order in terms of TOF (250 °C) is in harmony with the *T*_50_ activity order of the same series of doped-ceria solids, as shown in Figure 5C, suggesting that lattice oxygen on the surface is an important active species in the CO/O_2_ reaction.

**Figure 7:**
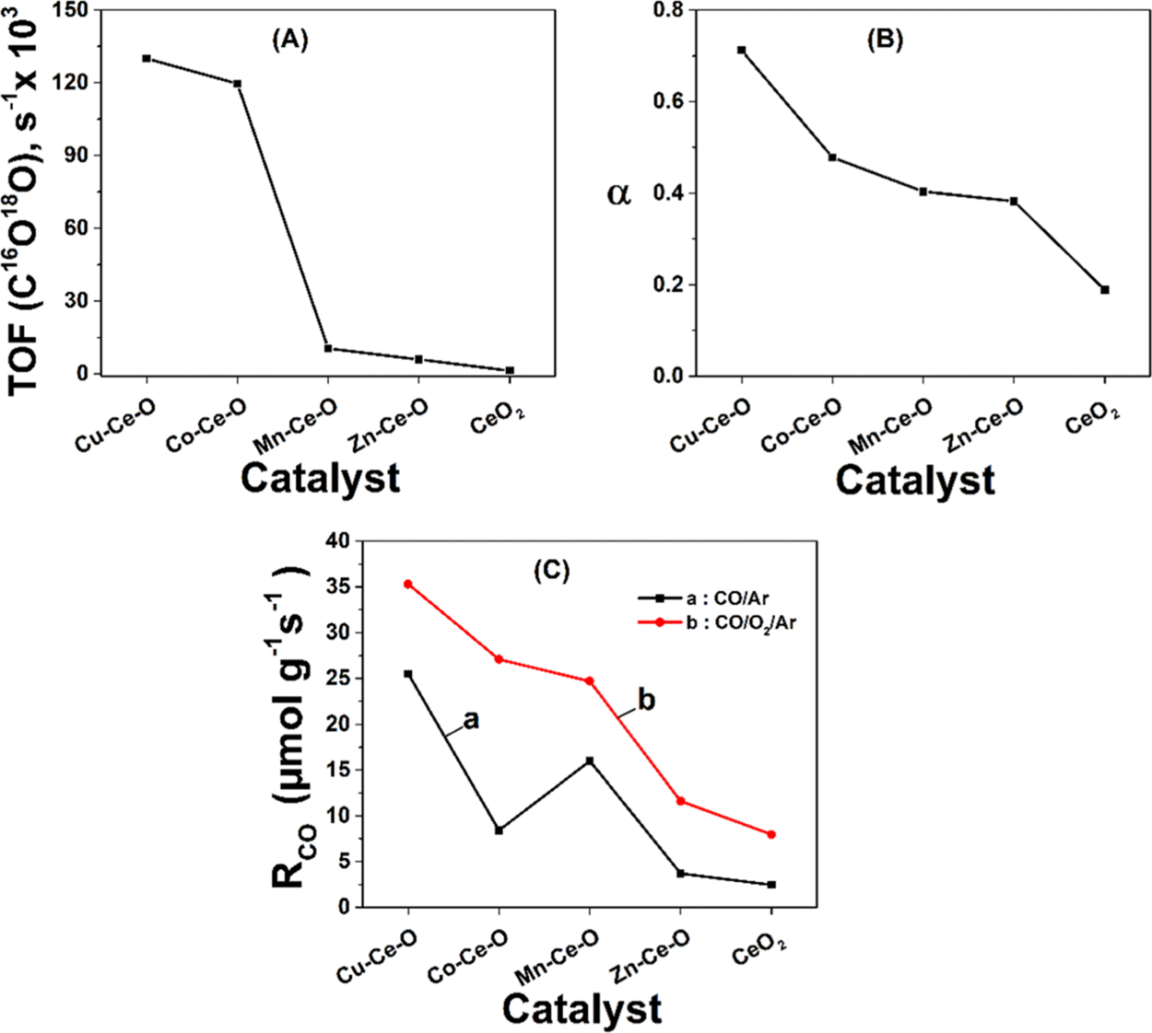
(A) TOF of *R*^max^ of C^16^O^18^O(g) formed during the step-gas switch Ar → 4 vol % CO/2 vol % Kr/Ar (20 min) performed at 250 °C on the TM-doped ceria (TM: Cu, Co, Mn, and Zn) and the pristine CeO_2_ catalysts. (B) Ratio (α) of the amount of C^16^O (mmol g^–1^) consumed to the ^18^O (μmol g^–1^) exchanged after 20 min in CO oxidation reaction on the same doped-ceria catalysts. (C) Maximum rate (μmol g^–1^ s^–1^) of CO(g) consumed during the step-gas switch (a) Ar → 4 vol % CO/2 vol % Kr/Ar (20 min) and (b) Ar → 4 vol % CO/15 vol % O_2_/2 vol % Kr/Ar (20 min) performed at 250 °C on the TM-doped ceria catalysts.

It was of interest to define the parameter α as the ratio of the amount of CO consumed to the amount of ^18^O-exchange during the 20 min CO/Ar reaction with doped ceria, following ^18^O-isotopic exchange (Figure 6A,B, Table 2). This parameter α was estimated and plotted against the composition of the TM-doped ceria solids, as shown in Figure 7B. It is illustrated that in spite of the fact that Cu–Ce–O, Co–Ce–O, and Mn–Ce–O solids have practically the same amount of ^18^O stored initially, before the switch to the reactive CO/Ar gas mixture, due to the fact that the site activity of Cu–Ce–O is the largest among the other solids, this leads to the largest value of the descriptor parameter α (Figure 7B). Of interest also is the practically same value of α obtained for Mn–Ce–O and Zn–Ce–O catalysts, where the solids appear to have very dissimilar amounts of initially available ^18^O lattice oxygen and initial reaction rates (amount of CO consumed), but the extent to which lattice oxygen reacts appears very similar.

The initial rates (μmol CO g^–1^ s^–1^) of CO oxidation reaction, *R*_CO_, in the presence of gaseous oxygen (4% CO/20% O_2_/Ar) at 250 °C for the same TM-doped ceria catalysts were measured and compared with those obtained in the absence of gas-phase oxygen (Figure 7C). It is illustrated that the participation of surface lattice oxygen is very important. For Cu–Ce–O, the *R*_CO_ due to surface lattice oxygen (CO/Ar) is ∼70% of the total *R*_CO_ (CO/O_2_/Ar; lattice and gas-phase oxygen are used), while in the case of Zn–Ce–O, it is ∼30%.

The increased CO oxidation rate in the presence of oxygen should also be connected to the strength of CO_2_ product chemisorption on the surface of TM-doped CeO_2_ solids (see Section 3.7), where decomposition of carbonate-type adsorbed CO_2_ is facilitated by the presence of adsorbed O_2_-s as shown in the following eq 16(56)
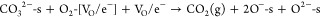
16

The molecularly adsorbed oxygen species shown in eq 16 is associated with a surface oxygen vacant site, V_O_. The formation of adsorbed O^–^-s species could also be considered to have higher reactivity toward CO(g) compared to other negatively charged surface oxygen species (ca. O^2–^).^57^

### 3.6 Identification of Active and Inactive Species in CO Oxidation

For investigating the reaction mechanism over the herein ceria-based mixed metal oxides, as well as the formation of carbonates being a potential active intermediate species, DRIFTS and SSITKA-DRIFTS experiments were designed and performed over selected metal oxides. In particular, Cu–Ce–O and Zn–Ce–O solids were investigated based on the highest and lowest catalytic performance provided, respectively, whereas CeO_2_ was used as a reference sample. The most important findings of this investigation are discussed below.

DRIFTS spectra were recorded in the 1700–900 cm^–1^ IR region after 30 min exposure of the Cu–Ce–O in ^12^CO/O_2_/Ar gas at 125 and 250 °C (Figure S11A). The IR bands at 1570 and 1296 cm^–1^ correspond to the stretching mode O–C–O of bidentate- and tri-dentate adsorbed carbonates, whereas the IR bands at 1473 and 1361 cm^–1^ correspond to monodentate and polydentate carbonates.^58^ For a consistent quantitative interpretation of the spectra, the Kubelka–Munk (K–M) analysis was used, as presented in Figure S11B. The carbonates are stable at both temperatures, even though their intensities decreased with increasing reaction temperature. The broadness of the IR bands in this region dictates the presence of more than one type of carbonate species for the examined solids and under the particular experimental conditions. DRIFTS spectra (Figure S11B) recorded in the 1700–900 cm^–1^ IR region after 30 min in ^12^CO/O_2_/Ar over CeO_2_, Zn–Ce–O, and Cu–Ce–O solids show that the broadness of the carbonate bands is largely varied as one moves from CeO_2_ to Zn–Ce–O and Cu–Ce–O. This has to do with the different surface heterogeneities of the solid compositions studied. Given that the carbonate bands appear at 1350 and 1480 cm^–1^, it can be concluded that CeO_2_ presents IR bands of carbonates at slightly higher frequencies compared to the doped-ceria solids (Figure S11B).

**Figure 8:**
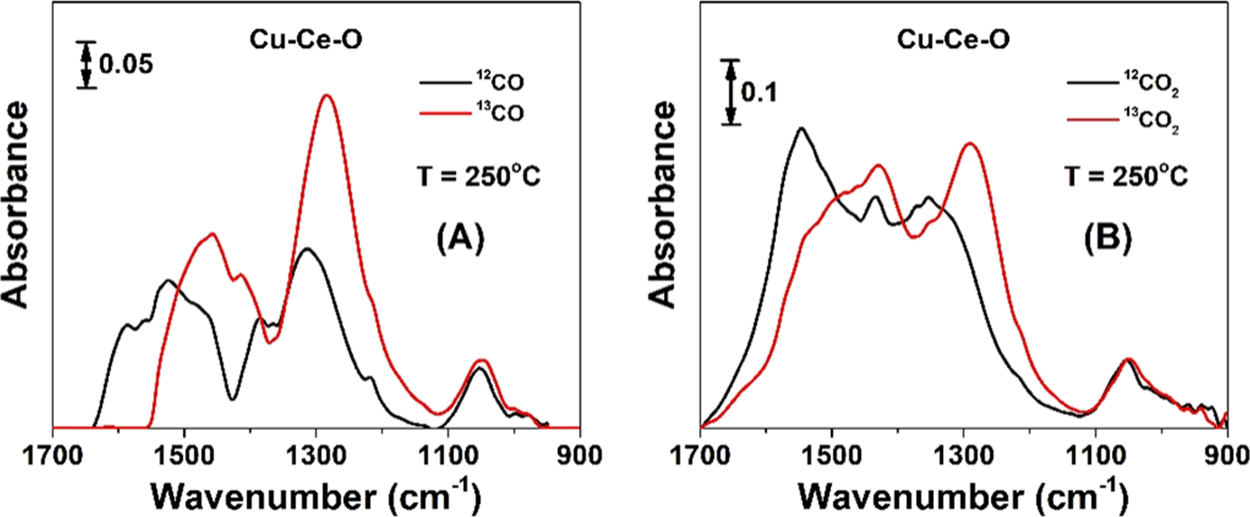
(A) SSITKA-DRIFTS spectra recorded in the 1700–900 cm^–1^ IR region after 30 min in ^12^CO/O_2_ at 250 °C following the step-gas switch ^12^CO/O_2_ (30 min) → ^13^CO/O_2_ (5 min) for the Cu–Ce–O solid. Feed gas composition of nonisotopic and isotopic gas: ^12^CO or ^13^CO = 4 vol %, O_2_ = 20 vol %, balance Ar; *F*_T_ = 50 N mL min^–1^. (B) DRIFTS spectra recorded in the 1700–900 cm^–1^ IR region after 30 min in ^12^CO_2_/O_2_ followed by the step-gas switch ^12^CO_2_/O_2_ → ^13^CO_2_/O_2_ (5 min) for the CeO_2_, Zn–Ce–O, and Cu–Ce–O solids. Feed gas composition of nonisotopic and isotopic gas: ^12^CO_2_ or ^13^CO_2_ = 2 vol %, O_2_ = 20 vol %, balance Ar; *F*_T_ = 50 N mL min^–1^.

Figures 8A and S12A,B present SSITKA-DRIFT spectra recorded during the step-gas switch ^12^CO/O_2_ → ^13^CO/O_2_ over Cu–Ce–O and CeO_2_/Zn–Ce–O solids, respectively. The presence of the red isotopic shift in the IR bands of adsorbed carbonate species indicates that these species are likely involved in the CO oxidation reaction mechanism under the experimental conditions employed.^20^ It should be noted at this point, however, that the observed isotopic shift might arise from the reversible adsorption of CO_2_ on the solid surface (forming carbonate-type species). This important point is presented and discussed next. By comparing the DRIFT spectra obtained over the Cu–Ce–O solid at 125 and 250 °C (Figure S11A), it can be seen that the IR bands at 1390 and 1480 cm^–1^, linked to carbonates, are more pronounced at 125 °C compared to those formed at 250 °C, which implies their thermal instability at the higher temperature and thus the lower surface concentration.

Figures 8B and S13A,B present DRIFTS spectra recorded over the Cu–Ce–O and CeO_2_/Zn–Ce–O solids following the ^12^CO_2_/O_2_/Ar → ^13^CO_2_/O_2_/Ar step-gas switch. The purpose of this experiment was to investigate the interaction of CO_2_ (CO oxidation reaction product) with the catalyst surface (formation or not of adsorbed carbonates). The carbonate IR band at 1580 cm^–1^ exhibits the ^13^C-isotopic shift, only in the case of the Cu–Ce–O catalyst but not in the case of Zn-doped ceria and pure CeO_2_. This implies at least that ^12^CO_2_ adsorption on the Cu–Ce–O catalyst is a reversible step, which is able to be exchanged with gaseous ^13^CO_2_. However, this important DRIFTS result on Cu–Ce–O (Figure 8B) and that of SSITKA-DRIFTS (Figure 8A) on the same catalyst do not necessarily imply that the formed carbonates are truly active intermediate species. On the other hand, the fact that no IR bands of adsorbed CO_2_ on pure CeO_2_ during SSITKA-DRIFTS in CO oxidation (Figure S12A) provided the red isotopic shift, this clearly illustrates that such carbonate-type species must be considered as spectator (inactive) species, likely contributing to the deactivation of the catalyst. In the case of the Zn–Ce–O catalyst, the isotopic switch ^12^CO_2_/^13^CO_2_ (Figure S13B) does not seem to provide the red isotopic shift. However, in the case of ^12^CO/^13^CO gas switch (Figure S12B), some red isotopic shift can be clearly seen in a region where overlapping with formate (HCOO) and carboxylate (COOH) species does occur. These adsorbed species are present as the result of reaction of adsorbed CO with −OH groups, and their formation could be a reversible step, thus giving rise to the red isotopic shift.

Based on the in situ DRIFTS study performed in the present work, CO can be adsorbed onto the CeO_2_-based metal oxides in the form of bidentate carbonate or carbonyl species (at low temperature). At the beginning of the reaction process, CO reduces Cu(II) to Cu(I) by removing a surface lattice oxygen species, thus forming CO_2_ and a surface oxygen vacancy, O_v_. The most energetically favorable surface copper species, facilitating the formation of O_v_, are those adjacent to the Cu heteroatom (Ce–O_v_–Cu), which dictates the importance of the CuO–CeO_2_ interface. In a following step, Cu(I) and O_v_ operate as centers for CO adsorption and O_2_ activation, respectively. The activated O_2_ species, likely O^2–^, react with Cu^+^-CO molecular species toward the formation of CO_2_ via a likely (cannot be excluded) carbonate-mediated mechanism.

### 3.7 Kinetic Studies of CO Oxidation

**Figure 9:**
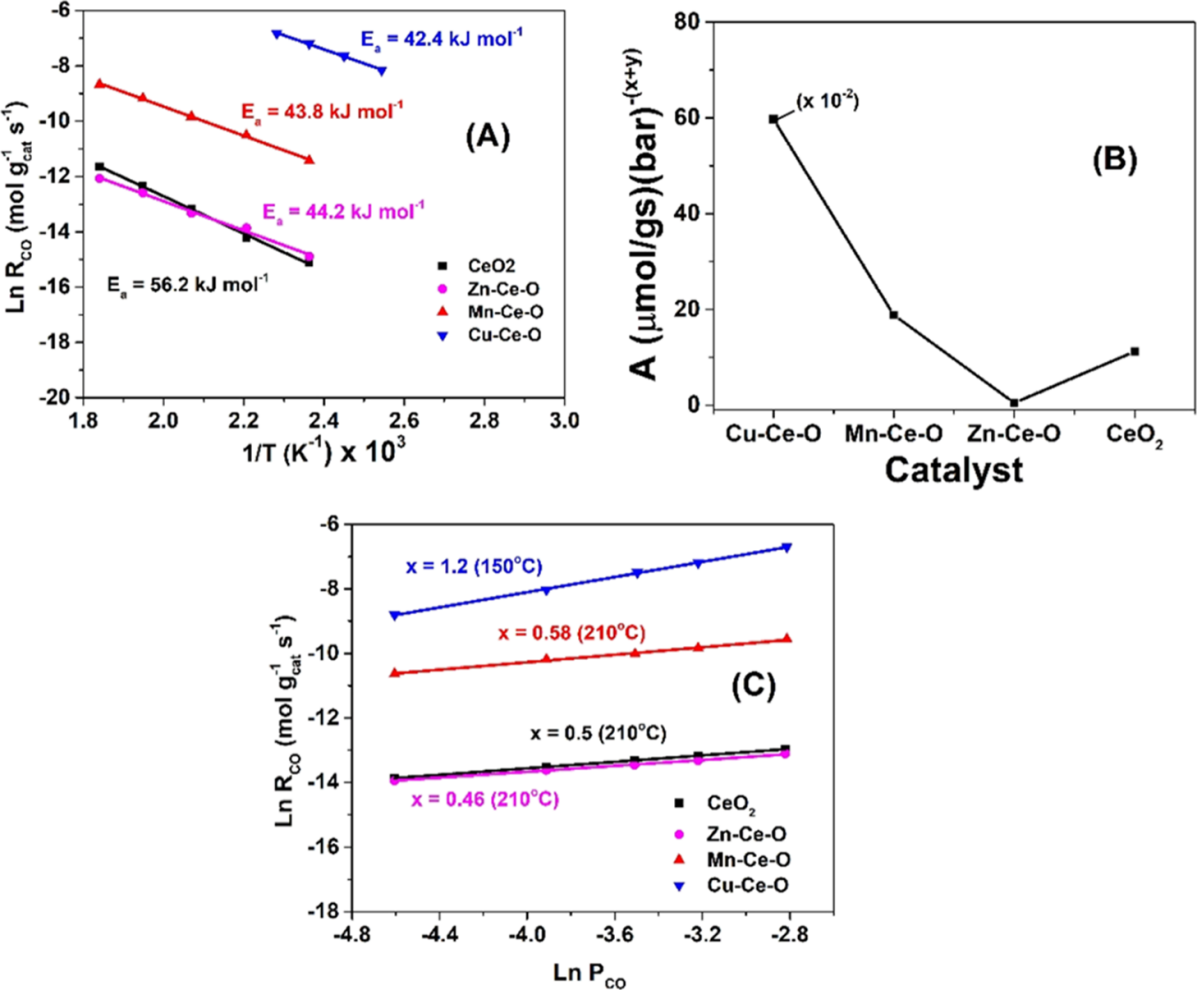
Determination of the kinetic apparent activation energy, *E*_a_ (kJ mol^–1^) (A), and apparent pre-exponential factor A (B) in the 120–270 °C range for Cu–Ce–O, Zn–Ce–O, Mn–Ce–O, and CeO_2_ solids. Reaction feed used 4% CO/20% O_2_/He. (C) Determination of the reaction order *x* with respect to the CO partial pressure in the CO oxidation reaction at *T* = 210 °C for Zn–Ce–O, Mn–CeO, and CeO_2_ solids and at *T* = 150 °C for Cu–Ce–O. The partial pressure of CO was varied in the 0.01–0.06 bar range; *P*_O_2__ = 0.2 bar; *P*_*T*_ = 1 bar.

The apparent activation energy (*E*_a_) of the CO oxidation reaction was estimated for the CeO_2_, Zn–Ce–O, Mn–Ce–O, and Cu–Ce–O catalysts in the 120–270 °C range (Figure 9A), as described in Section 2.4.

After plotting the Ln(rate_CO_) versus 1/*T*, *E*_a_ values in the 42.4–56.2 kJ mol^–1^ range were estimated. The pristine ceria and the Cu–Ce–O catalyst present the highest and lowest value, respectively. The apparent activation energy values follow the same order as the activity profile, as shown in Figure 9. For comparison purposes, similar composition catalysts such as CuO/CeO_2_ and Cu_0.01_Ce_0.99_O_2–*x*_^59^ showed activation energies of 41 and 70 kJ mol^–1^, respectively.

On the other hand, the pre-exponential factor (*A*) was found to be strongly dependent on catalyst composition (Figure 9B). Details of the kinetic analysis conducted are presented in the Supporting Information (see Section S2.7. Kinetic studies, Figure S14 and Tables S4–S14). As discussed next, the RDS of the present catalytic CO oxidation reaction is considered to be that of adsorbed CO with the surface lattice oxygen of the mixed metal oxide via a Mars–van Krevelen (MvK) type of mechanism. An increase in the number of active catalytic centers usually corresponds to a higher value of *A* as empirically estimated (eq 2). On the other hand, this *A* parameter is also linked to the entropy change (Δ*S*) between the adsorbed CO-s and O-L (surface lattice oxygen) and the associated transition state. Large differences in the *A* kinetic parameter over Cu-doped ceria and other supported metal oxides were reported.^60^

As part of the kinetic analysis, the reaction orders with respect to CO and O_2_ were also estimated as described in Section 2.4. The rate of CO oxidation was found to increase with increasing *P*_CO_ (Figure 9C), with the highest dependence (*P*_CO_^1.2^) in the case of the most active catalyst, Cu–Ce–O. The dependence of *R*_CO_ on *P*_CO_ follows the order Cu–Ce–O (*P*_CO_^1.2^) > Mn–Ce–O (*P*_CO_^0.58^) > CeO_2_ (*P*_CO_^0.5^) > Zn–Ce–O (*P*_CO_^0.46^). On the other hand, zeroth-order dependence (or independence) was found with respect to the oxygen composition (*P*_O_2__), as illustrated in Figure S14. These findings agree very well with recent kinetic work on various doped-ceria solids (Pr-, Gd-, or Nb-doped ceria), where the dependence of the CO oxidation rate on *P*_CO_ varied in the 0.6–0.9 range (350 °C; 1% CO/1% O_2_/He), while a zeroth-order rate dependence was found on *P*_O_2__.^61^ These findings strongly suggest that adsorbed CO-s on the surface participates in the rate-determining step (RDS) in the reaction path, which is not the case for the adsorption of oxygen, and the required reoxidation of the catalyst is faster than the RDS.^61^ These results are corroborating for an MvK type of mechanism for the CO oxidation reaction, where formation of an oxygen vacancy is included in the RDS.

**Figure 10:**
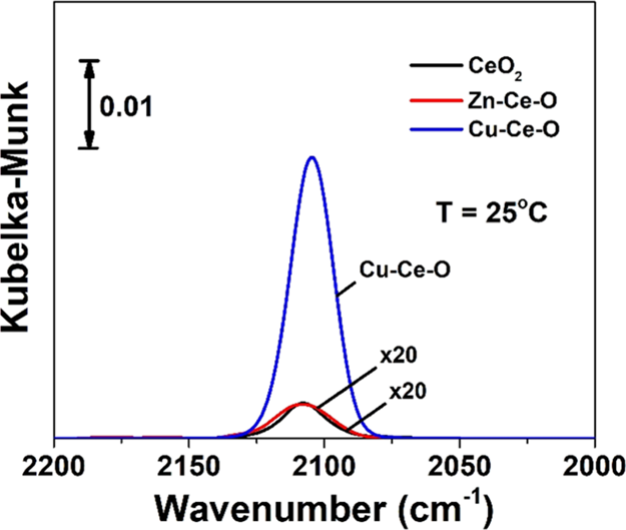
In situ DRIFTS spectra recorded in the IR region 2200–2000 cm^–1^ over CeO_2_, Zn–Ce–O, and Cu–Ce–O solids after the following gas treatment: CO/O_2_/Ar at 250 °C/30 min, cool down to 25 °C in CO/O_2_ followed by Ar purge (1 min), and spectrum acquisition. Feed gas composition: CO = 4 vol %, O_2_ = 20 vol %, balance Ar; *F*_T_ = 50 N mL min^–1^.

Figure 10 presents in situ DRIFT spectra recorded in the IR region of 2200–2000 cm^–1^ over CeO_2_, Zn–Ce–O, and Cu–Ce–O solids after their exposure for 30 min at 250 °C in the 4% CO/20% O_2_/Ar gas mixture and cooling at 25 °C in the same gas mixture, followed by Ar purge (1 min). The IR band in the 2200–2000 cm^–1^ range corresponds to linear adsorbed CO on Cu^+^ active sites (or metal cations, in general); particularly, in the case of the Cu–Ce–O catalyst, Cu^+^-CO carbonyl species was assigned to this IR band (centered at ∼2105 cm^–1^).^63^ Based on the integral band area provided in K–M units, it can be stated that apart from the Cu–Ce–O catalyst, where the concentration of adsorbed CO is profound (higher coverage), the other two catalysts, namely, CeO_2_ and Zn–Ce–O, present only a very small amount of adsorbed CO (very low coverage). These results suggest that CO is adsorbed according to the MvK mechanism, and thus, an Eley–Rideal mechanism might be excluded. Furthermore, these results agree very well with the kinetic analysis presented previously (Figure 9) along with the catalytic activity performance results (Figure 5) and corroborate the fact that the high CO surface coverage over Cu–Ce–O led to higher kinetic rates for the CO oxidation reaction.

### 3.8 DFT Calculations

**Figure 11:**
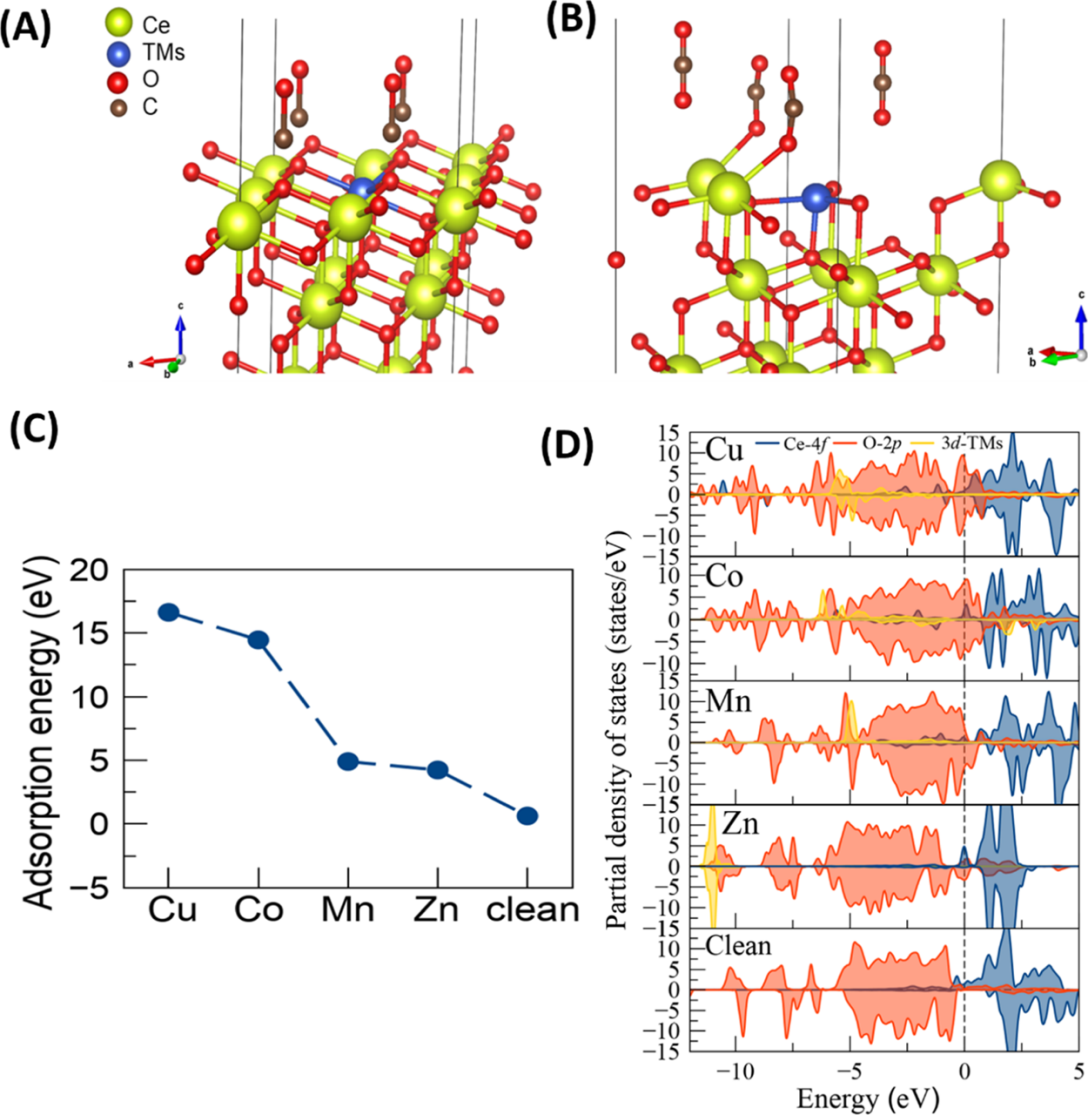
(A,B) Surface model for CO adsorption on the *p*(2 × 2) lateral unit cell of CeO_2_(111) with the substitution of TM (Mn, Co, Cu, and Zn) heteroatoms in the Ce surface lattice. (A) Initial surface with placement of the CO molecule on the most favorable site of adsorption, which is the oxygen surface atom, (B) similar to (A) but fully relaxed atomic structure. (C) Adsorption energy of CO as a function of TM (the clean surface of nondoped ceria is also given). (D) Partial density of states of TM (Cu, Co, Mn, and Zn)-doped CO/CeO_2_(111) compared with the clean CO/CeO_2_(111) surface (undoped ceria).

To better understand the impact of the TM heteroatoms investigated in the present work on the chemical reactivity of ceria-(111)/CO system, we first investigated the adsorption of CO on a *p*(2 × 2)-CeO_2_ lattice in the presence of the TM heteroatom by DFT calculations, as depicted in Figure 11A. According to the optimized atomic structures for all the TM heteroatoms shown in Figure 11B, the surface lattice oxygen is strongly bonded to the CO molecule, where the former relaxes upward from its site toward the carbon atom, accompanied by the formation of CO_2_ and the release of heat (exothermic reaction). This process in turn is leaving more oxygen vacancies when doped CeO_2_ is compared with pure CeO_2_. Because of the small size of the TM cation compared to Ce^4+^, the substitution of TMs in the topmost layer of the surface induces a strong local geometrical relaxation. All TM heteroatoms show an inward relaxation even stronger than that of Ce and become almost coplanar with the oxygen in the layer beneath. Consequently, the TM heteroatoms prefers the fourfold coordination environment with their neighboring O atoms instead of the sevenfold, before relaxation and creation of an O vacancy (Figure 11B). The average bond length of Cu–O for the fourfold coordination was calculated to be 1.84 Å, ∼3% smaller than that in the ideal CuO bulk structure, ca. 1.90 Å.^62^ A similar behavior was experimentally observed for Cu-doped ceria,^63^ where the coordination shell of Cu–O bonds can exist in either threefold or fourfold modes for the neighboring oxygen atoms, depending on the concentration of the Cu heteroatom and its degree of oxidation.

We have to emphasize, however, that the *p*(2 × 2) lateral unit cell of CeO_2_(111) allows for more degrees of freedom, and thereby, the O surface atoms, especially those near to the TM heteroatoms, are easily displaced outward from the surface along the *z*-direction by 1.80 Å, resulting in a C–O bond length of 1.17 Å with an O–C–O angle closer to 180°. The nature of bonding in the formed CO_2_ from one oxygen surface atom and an adsorbed CO is almost identical to the ideal CO_2_ molecule, in qualitative agreement with the recent first-principles study. More specifically, as can be observed in Figure 11B, the CO molecule closer to the TM atom is clearly more oxidized, resulting in the formation of CO_2_ that floats free on the ceria surface, according to the CO oxidation reaction process described in eq 17
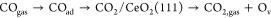
17

This reaction process, however, is strongly linked to the oxygen vacancies induced by the TM heteroatoms, which play a central role in the catalytic activity of the ceria surface. This behavior is carefully analyzed by comparing the adsorption energy and the chemical trend for the considered TM-doped CeO_2_(111) surface. In this case, we calculated the adsorption energy at full coverage of CO on a relaxed TM-doped CeO_2_ surface, where the energies of CO adsorption on the most favorable site, which is the atop oxygen surface site, are determined for the various TMs. Based on these results, the CO adsorption energy, which is a good indicator of the surface reactivity of ceria, shows a clear chemical trend: *E*_CeO_2__ < *E*_Zn–CeO_2__ < *E*_Mn–CeO_2__ < *E*_Co–CeO_2__ < *E*_Cu–CeO_2__ as depicted in Figure 11C. This indicates that the Cu–CeO_2_ surface is chemically more reactive than the other TM-doped ceria surfaces. More specifically, the adsorption of CO on the Cu-doped CeO_2_ surface is found to be more favorable, ca. −16.63 eV, followed by Co (−14.46 eV), Mn (−4.90 eV), and Zn (−4.24 eV), followed by pure CeO_2_ (−0.63 eV), which is significantly lower in comparison with the TM-doped ceria surfaces. This tendency with respect to the CO adsorption step is perfectly predicted for the oxidation activity of CO/CeO_2_(111), where the created oxygen vacancy in Cu-doped CeO_2_ requires only a lower penalty. Thus, higher mobility of oxygen surface atoms could be more easily achieved by considering Cu atom insertion in the ceria crystal lattice. These important findings prove again that the reaction path of CO oxidation (see eq 16) can be largely facilitated when ceria is doped with Cu atoms or by considering an admixture of TM aliovalent heteroatoms.

To get further insights into the localized surface states and their occupation, the electronic partial (projected) density of states (PDOS) and the corresponding Bader charges were estimated. At first glance, the most salient feature of CeO_2_(111) doped with TMs is the appearance of new surface states in the band gap, as shown in Figure 11D. Note that the clean ceria surface is known to be an insulator with an energy band gap of 2.2 eV between the O 2*p* states, to possess valence-band maxima, and having Ce 4f states as minimum of the conduction band. As clearly seen in the partial density of states versus energy graph (Figure 11D), the defect peaks that correspond to the partially occupied 2p O orbitals become more occupied to lower energies in comparison to their positions in the case of Cu-doped CeO_2_, when changing the TM from Cu to Zn. This is also accompanied with a noticeable downward shift of the valence density of states (dominated by O 2p states), with respect to the Fermi level. Furthermore, the Ce 4f states are lowered toward the Fermi level and strongly hybridized with the O 2p orbitals of the neighboring oxygen atoms of the surface layer, which indicates the reduction of Ce^4+^ to Ce^3+^.

The increasing delocalization of the Ce 4f wave function can be explained by the fact that oxygen vacancy formation increases the number of excess electrons in the surface layer (extra electrons left by the neutral O vacancy), thus inducing a significant charge compensation, mostly by Ce atoms in order to maximize the extent of bonding with the O neighbors. The charge compensation mechanism is supported by the analysis of the Bader charges, which is very useful to further understand the adsorption trend with respect to the 3d elements. As mentioned above, the incorporation of TM atoms reduces the coordination to only fourfold coordination with O neighbors compared to the sevenfold coordination shell of the Ce–O bonds. However, it should be noted that the Cu atom compensates more charge (0.37*e*^–^), which is almost three times compared to Co and Mn heteroatoms, ca. 0.13 and 0.10*e*^–^, respectively.

In the case of the Zn-doped CeO_2_ surface, despite the presence of oxygen vacancies as for the other TM heteroatoms, its low CO adsorption energy compared to the other TMs can be explained by the fact that the Zn heteroatom is found to be neutral. In order to maintain almost the same oxidation state, no charge compensation occurs during adsorption of CO compared to the clean surface without the adsorbate. The calculated Bader charges of Zn-doped CO/CeO_2_ amounts to 10.80*e*^–^ compared to 10.84*e*^–^ for the Zn-doped CeO_2_ system, indicating that the Zn atom maintains almost the same electronic charge, and therefore, no reduction of Zn takes place. We also observed that the density of O 2p surface states and their hybridization with Ce 4f orbitals around the Fermi level decrease in the order Cu > Co > Mn > Zn > clean ceria, as can be seen in Figure 11D. This in turn suggests the lowering in the catalytic activity of the ceria surface, which explains the adsorption trend observed over the 3d elements.

#### 3.8.1 Bridging DFT Calculations with Experimental Analysis

Structure-DFT: Based on the XRD studies, values of lattice parameters were obtained. The decrease in lattice constant/parameters values is due to the fact that the distance of M–O (e.g., Fe–O: 2.116 Å and Co–O: 2.137 Å) is shorter than the Ce–O bond (2.343 Å). This is because the electronegativity of Fe and Co atom is higher (1.83 and 1.88) when compared to the Ce atom (1.12),^64^ whereas Fe^3+^ (0.55 Å) and Co^3+^ (0.54 Å) ionic radii are smaller than the Ce^4+^ radius (0.90 Å).^64^ The lattice constant of doped ceria is smaller as a result of the oxygen vacancy formation. At the same time, the formed O_v_–TM pair interaction forces the M–O bond to be uneven (not uniquely defined), thus breaking the localized symmetry. This is reflected through the Raman F_2g_ band broadening and intensity lowering and the low intensity Raman peaks <400 cm^–1^. Upon Ce atom substitution in the lattice by the TM element, as proved by the XRD and Raman findings mentioned above, one or more holes (states) are formed below the Fermi level, acting as (electron) acceptors (Figure 11D). These states facilitate the acceptance of electrons and thus maintain the O_v_ formation.

#### 3.8.2 Structure/Activity/Kinetics-DFT

The linear adsorbed CO species on the Cu–Ce–O, Zn–Ce–O, and pristine ceria catalytic surfaces, as proved using in situ DRIFTS (Figure 10), are in agreement with the DFT calculations (Figure 11). The higher CO coverage, as revealed for the case of Cu–Ce–O, corroborates for the higher intrinsic activity; this is also generally reflected in the apparent activation energy (*E*_app_), where low *E*_app_ values correspond to high catalytic activity, although in the herein study, kinetic studies showed that the difference in the *E*_app_ values among the catalysts are small. Conversely, large differences in the pre-exponential factor (*A*, entropic factor) were found, which had strong dependence on the nature of the dopant metal cation (Figure 9B). The order of the pre-exponential factor found was Cu > Mn > Zn > CeO_2_ in good agreement with the CO adsorption energy as calculated using DFT (Figure 11C), Cu > Mn > Zn, and with the catalytic activity order. At the same time, another descriptor for the CO oxidation activity is the energy of oxygen vacancy formation (*E*_vf_). As explained above, heteroatom integration in the cubic lattice (based on the XRD) increases the O_v_ population (based on Raman studies, band at ∼540 cm^–1^) and the Ce^3+^ species on the surface (based on XPS) with a concomitant boost of the ceria redox properties and oxygen mobility (H_2_-TPR and ^18^O experiments). Krcha et al.^14^ established some useful periodic trends; an increase in atomic radii leads to an increase in *E*_vf_ (easier O_v_ formation). When TM heteroatoms are used as the dopant into the ceria lattice, it has been proposed that the enhancement in CO oxidation activity originates 50% by structural relaxation due to the TM presence and 50% due to the new mid-states formed (DOS studies), the latter able to host the electrons left behind upon the formation of the Ov.^65^ In our DFT study, DOS results showed that the O 2p–Ce 3f hybridization follows the Cu > Co > Mn > Zn order in good agreement with the catalytic activity, while Cu can host much more charge (Bader analysis).

#### 3.8.3 Interpretation of the CO Adsorption Energy

The CO adsorption energy, as calculated based on the DFT studies, is a very good descriptor of catalyst’s activity in building a volcano curve and in the Brønsted–Evans–Polanyi (BEP) relationship and allows to predict activity trends.^66^ The catalyst structure is what dictates the adsorption energy, and thus by tailoring experimentally the structure, a very active catalyst can be designed (in terms of adsorption and activity). There are many studies in the literature toward understanding the binding energy of a surface.

According to the DFT calculations presented here and the CO adsorption energy values reported (Figure 11C), it seems that the Cu–Ce–O catalyst is most likely at the peak of a volcano curve of activity. There are examples in the open literature, where the volcano type of activity for a series of metals toward the CO oxidation reaction has been studied through the development of accurate DFT models, for example, models that include adsorbate–adsorbate interactions. These models proved that adsorbate–adsorbate interactions increase the activity of the TM which presents high binding energy (*E*), but they do not interfere with the relative order of activity of the metals. In the case of CO/O_2_ reaction, it was found that the lowering of the O and CO binding energies (*E*_O_ and *E*_CO_) can occur more readily on surfaces that have high initial binding energies that reasonably correspond to higher coverages (e.g., the case of the Cu–Ce–O solid in this study). Thus, it can be stated that the high binding energy over the Cu–Ce–O system corresponds to high coverage, as this was also proved using DRIFTS studies (Figure 10), although this does not present any barrier for the reaction, as the lateral CO–CO interactions are anticipated to make milder the binding state of CO on the surface.^66^

The adsorption energies reported herein refer to CO adsorption only. In the case of the simultaneous presence of O and CO, due to the different sites being responsible for each one of the reactants, an additional term is needed for the DFT models to account for the integral binding energy (error of these calculations is max ∼0.1 eV). Combination of the above-described DFT model, where the adsorbate–adsorbate interactions are taken into account, with a microkinetic model for the CO oxidation reaction can lead to accurate calculation of the coverage dependent volcano activity, where the parameters *E*_O_ and *E*_CO_ are the independent parameters. By comparing the developed models with and without accounting for the adsorbate–adsorbate interactions, it can be stated that the volcano curve shape is affected by the interactions, especially when *E*_CO_ is high. In contrast, the activity trend and the maximum of the volcano curve are not affected. In addition, the structure sensitivity of the volcano activity curve has been probed in the literature, performing calculations for metals, on closed packed structures, and over nanoparticles (12 atom clusters); the latter is expected to exhibit a higher population of low-coordination sites. The two types of surfaces have distinct and different maxima in the volcano plot because of the structure sensitive nature of the adsorption energy (*E*_CO_) and activation energy (*E*_act_). This can be a very useful design tool as it shows that less reactive metals can turn to more reactive ones when the active sites are dominated by low-coordination atoms.

### 3.9 Mechanistic Insights—Descriptors of Oxidation Activity and Periodic Trends

#### 3.9.1 MvK Mechanism

It is widely accepted that CO oxidation on reducible metal oxide surfaces follows an MvK mechanism, where all the elementary steps are linked to the lattice oxygen or adsorbed molecular O_2_ on faces of the polycrystalline metal oxide materials. In particular, in pristine ceria, Ce^4+^ is reduced to Ce^3+^ by CO, while an oxygen vacancy (O_v_) is formed. Upon the formation of the vacancy, an excess of two electrons are left behind to fill the 4f Ce^4+^ orbitals converting it to Ce^3+^. This is followed by reaction of the O_2_ (feed gas) with the solid surface to fill the oxygen vacancy created (O_v_). This step is also known as O_2_ activation during which very active atomic oxygen species are formed. In the last step, CO reacts with the highly active atomic oxygen toward the formation of CO_2_.

Upon doping with a TM (e.g., Cu, Ni, Mn, Co, and Zn), additional oxygen vacancies (O_v_) are formed due to the TM heteroatom insertion into the CeO_2_ cubic lattice (e.g., Cu^2+^ and Ce^4+^ charge compensation), resulting in a highly reducible doped-ceria lattice. As already mentioned, two extra electrons are remaining on the oxygen vacancy upon its creation. Based on the Bader charge analysis performed in this study, Cu receives more negative charge compared to Co, Mn, and Zn, and this corresponds to reduction of Cu^2+^ to Cu^1+^ (active site for CO oxidation). In the order Cu > Co > Mn > Zn, the TM favors the formation of additional energy states close to the Fermi level, as proved through the DOS study. The order reflects the great tendency of Cu to maintain the formation of O_v_ with low energy penalty. More energy states also lead to a higher degree of hybridization between Ce 4f and O 2p orbitals (DOS results, Figure 11). Oxygen vacancies can have an additional role, that of enhancing the dissociation of reactants.^67^ The strong redox properties of the doped ceria can be seen in the H_2_-TPR studies (see Figure 4C,D). Especially, the reduction of Cu–Ce–O takes place at much lower temperatures than the pristine CuO (260 and 207 °C) corroborating for a structure with a lower energy for oxygen vacancy formation.

**Figure 12:**
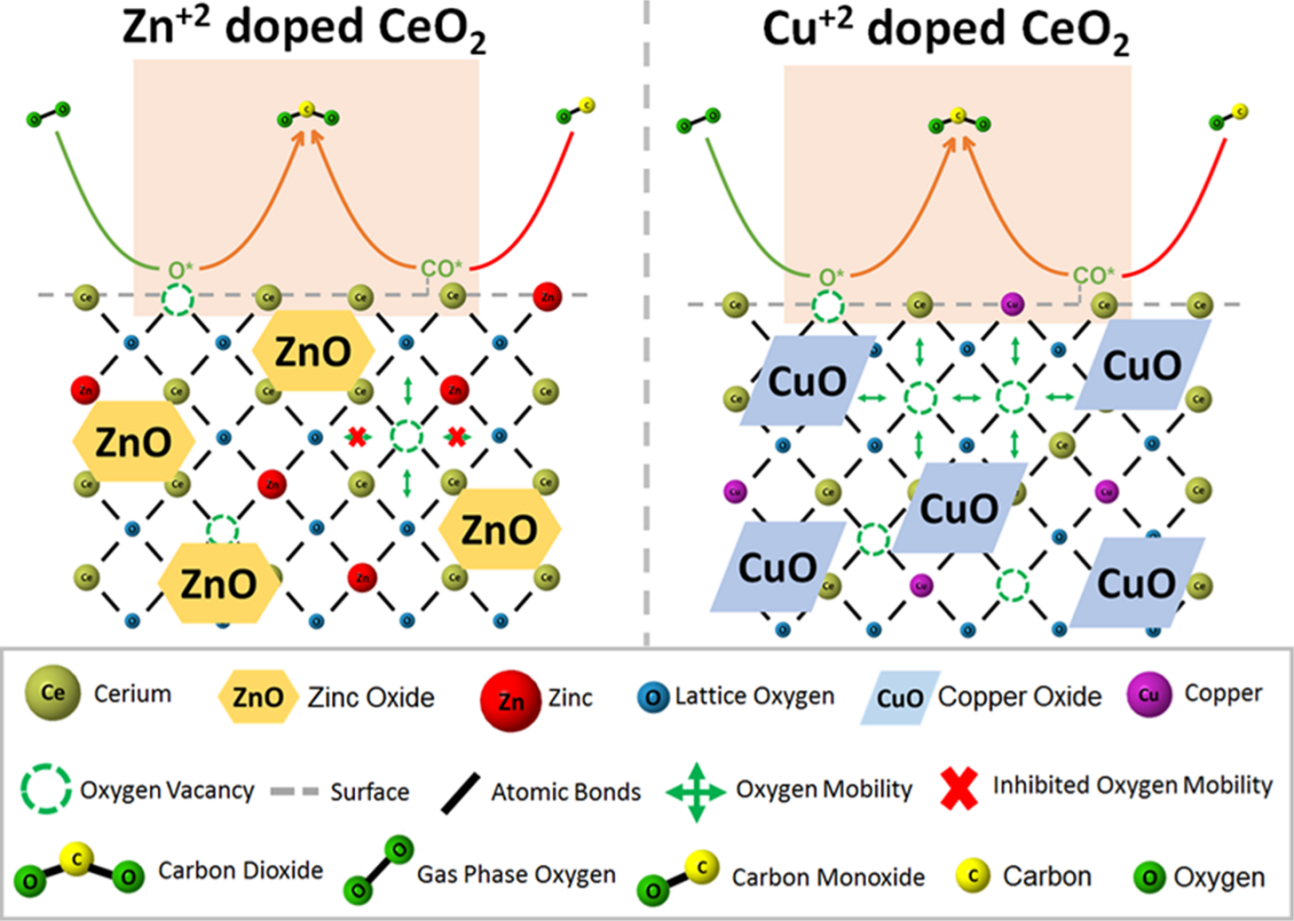
Schematic representation of the MvK mechanism governing the catalytic CO oxidation reaction over the TM-doped ceria (TM = Cu, Co, Mn, and Zn).

According to McFarland and Metiu,^3^ an LVD is responsible for the formation of strong Lewis acid sites, thus leading to strong chemical bonds with Lewis bases, such as the CO molecular species. This is reflected to the energy of CO adsorption calculated in the present DFT studies (see Section 3.8). Previous reports^68^ show that CO binds to a surface oxygen, preferably to the ones adjacent to the dopant, toward the formation of the O_s_–CO entity (O_s_: surface oxygen) for which the C–O bond lengths are similar to those in the gaseous CO_2_ molecule. Upon desorption of the CO_2_, an oxygen vacancy (V_O_) is left behind with localized electron charge previously carried by the O_s_. The oxygen vacancy is considered as the Lewis base, whereas gaseous O_2_ (O_2,gas_) is a Lewis acid and thus is readily adsorbed and activated onto this specific surface site. The transient isotopic response curves of CO_2_’s presented in Figure 6B,C, and the comparative initial maximum transient rates of CO consumption and C^16^O^18^O formation for the various TM-doped CeO_2_ presented in Figure 6A,C illustrate the occurrence of the MvK mechanism on the present TM-doped ceria surfaces, the latter shown schematically in Figure 12. Briefly, in Figure 12, the phase heterogeneity of the Zn-doped and Cu-doped ceria catalysts is presented along with its consequence on the population and the mobility of the oxygen vacant sites. The structural characteristics are put together in Figure 12 as contributors in the MvK mechanism of the CO oxidation reaction. In the present work, the MvK mechanism was demonstrated through the *a* descriptor parameter under anaerobic conditions (Figure 7C). The surface lattice oxygen is the only oxygen source in the system that determines the initial rate of reaction. It is also known that the presence of defects (e.g., oxygen vacancies) increases the mobility of lattice oxygen in ceria.

The increase in intrinsic anionic defect sites leads to a boost in lattice oxygen mobility due to the freedom that is provided for the oxygen movement. At the same time, the concentration of defect sites in ceria is tailored based on the surface facets, so it can be stated that the oxygen mobility is determined by the ceria surface structure. Depending on the ceria and the doping level, the sites with a low coordination number and the defect sites vary, affecting the energy of the surface oxygen vacancy formation. In the work reported by Krcha,^14^ it is mentioned that the valence of the dopant also affects the oxygen next to them (nearest neighbor, NN) in a different way; dopants of high valence affect the oxygen atoms next to them, whereas dopants of low valence affect oxygen atoms both in long and short ranges.

#### 3.9.2 Energy of Vacancy Formation, *E*_vf_

Previous reports^69^ have shown that the energy of vacancy formation (*E*_vf_) calculated by DFT tools can be a major descriptor of activity in the study of CO oxidation on doped-CeO_2_ surfaces. In the study reported by Kim and Han,^69^ the *E*_vf_ follows the order Cu (−0.68 eV) < Ni (−0.39 eV) < Mn (0.09 eV) < Co (0.21 eV) < Fe (0.36 eV) < Cr (0.71 eV) < Ce (2.79 eV).^44^ In another study reported by Aryanpour et al.,^70^ the *E*_vf_ follows the order Zn(0.5) < Cu(0.8) < Fe(0.9) < Mn(1.2) < Co(1.3) < Ni(1.4) < Zr(1.7 eV). These two series of *E*_vf_ trends based on the results of Krcha et al.^14^ and Aryanpoor et al.^70^ are in good agreement with that found in the present catalytic activity order Cu > Co > Mn > Zn. The following considerations are relevant to this CO catalytic oxidation activity order:

(a) The small discrepancy in *E*_vf_ (eV) between Co and Mn can be due to the magnetic properties of these two elements.
(b) The Zn heteroatom even appeared to have the lowest *E*_vf_, and it is reported not to be the best dopant for ceria, since it does not introduce high structural distortions in the ceria matrix. The Zn dopant is not a good dopant since it cannot adopt square planar coordination. Instead, it forms four long and four short Zn–O bonds in tetrahedral coordination. In contrast, Mn, Fe, Ni, Co, and Cu induce very high structural distortions in the lattice. The TM dopants usually adopt a coordination environment of four nearest neighbors, either in a tetrahedral or square planar arrangement, as discussed in the present DFT studies (Section 3.5).

#### 3.9.3 Heteroatom Size and Valence

Another descriptor of the activity of doped ceria is the heteroatom size. It has been reported that as the size of the heteroatom (atomic radii) increases, the vacancy formation energy increases. When the heteroatom is smaller in size than Ce^4+^, as all the heteroatoms in the present study (Cu, Ni, Co, Mn, Fe, and Zn), the oxygen vacancies are formed in the nearest-neighbor position (NN), leading to “vacancy-dopant” entities. Also, due to the ordering of vacancies, vacancy–vacancy interactions can also occur.^3^ It has been found that LVD affects the energy of formation of oxygen vacancy even far from the vacancy site. This is due to the electron deficiency introduced, thus affecting the bonding of the electrophilic oxygen atoms on the surface.^3^

#### 3.9.4 Reducibility of TM-Doped CeO_2_

As it was reported by Krcha et al.,^14^ the TMs located at the left of the periodic table (groups 4 and 5, e.g., Ti and V) tend to modify the reducibility of Ce host atoms, whereas the TMs on the right (groups 10, 11, and 12, e.g., Ni, Cu, and Zn) are subjected into reduction themselves. The TMs in the middle of the periodic table exhibit an amphoteric role in surface reducibility. In particular, according to the same study,^14^ for Mn–Ce–O, Fe–Ce–O, and Co–Ce–O, reduction of Ce and the TM dopant takes place, whereas for Ni–Ce–O, Cu–Ce–O, and Zn–Ce–O, only the dopant is reduced. Zr is the only TM dopant from the herein study that is expected to cause reduction of only the Ce cation. Bader charge analysis (see Section 3.5) showed that Cu is almost three times more prone to reduction compared to Co and Mn, whereas Zn is not significantly subjected to reduction. The different reduction models of dopants during CO oxidation can be linked to the different active reaction intermediates, such as formates (HCOO), carbonates (e.g., ionic CO_3_^2–^), or oxygen species (e.g., O^–^ and O_2_^–^) formed during the CO oxidation reaction.

#### 3.9.5 Oxygen Vacancy Formation

Upon the formation of an isolated oxygen vacancy on the subsurface, surface relaxations are taking place, such as those of Ce atoms adjacent to the vacancy, which are moving away from it, whereas O, which is a second neighbor, is moving closer to the vacancy. These phenomena are crucial for the accommodation of the two extra electrons that accompany the O_v_ formation.^68^ It is known that O_v_ formation is thermodynamically favorable upon doping.^65^ At higher temperatures, next to the dopant, an oxygen vacancy is formed, and the easiness of the formation of a second vacancy remains a crucial parameter for the catalytic activity of doped ceria. This is true when dopants are isolated, although clustering of dopants can also happen. Another very critical factor is the interface between the dopant and the ceria oxygen atoms, whereas the energies of O_v_ formation show a clear surface dependence on the easiness of the formation of the second vacancy, close to the dopant.

## 4 Conclusions

The aim of this work was to perform a systematic investigation to quantify the effect of various TMs of different charges and cationic sizes introduced in ceria (doped CeO_2_) on its performance for the catalytic oxidation of CO. The catalytic activity of TM-doped CeO_2_ obtained follows the order Cu > Co > Ni > Mn > Fe > Zn > pristine. An insightful investigation was performed by the combination of advanced transient kinetic experimental techniques and ab initio tools (DFT computations) corroborating these findings. The following remarks are to be noted from the results of this work:

(a) The TM heteroatoms boost ceria activity by forming substitutional solid solution (Cu and Zr), interstitial solid solution (Fe), or segregated oxides (Zn, Ni, Mn, Fe, and Co) (XRD and Raman studies). The formation of substitutional/interstitial solid solution or solid solution + segregated oxides is taking place to different extents among the investigated TM heteroatoms based on meticulous TEM–EDS analysis along with XRD and Raman findings. This dictates the extent of interaction between the heteroatom and the ceria matrix as also supported by the redox behavior of the doped-ceria solids (H_2_-TPR studies) and the ΔBE value associated with the lattice oxygen (XPS studies) obtained between the pristine and doped ceria. The TM/Ce ratios as calculated using TEM–EDX and STEM–EDX showed that elemental composition of dopants in individual nanoparticles of ceria is less than their composition at a larger scale. Different degrees of interaction can also affect the species that are formed during oxygen activation of the doped ceria, such as superoxide (O^2–^) and peroxide (O_2_^2–^) intermediates, as demonstrated by the XPS studies. The method of preparation of TM-doped CeO_2_ adopted in the present study (microwave-assisted sol–gel synthesis) seems to favor the increased interaction between ceria and the heteroatom, as proven by critical comparison with other reports in the literature, where doped ceria has been prepared following precipitation rather than the microwave-assisted (present work) synthetic routes.
(b) Upon TM introduction in the ceria lattice, all dopants induced serious lattice distortions (tensile lattice strain) due to the TM preference to adopt the coordination number of four instead of eight as in the pure ceria matrix. Thus, the charge compensation mechanism leads to oxygen vacancy formation. The charge compensation mechanism was supported by Bader charge analysis, where it was found that Cu can compensate more charge (0.37*e*^−^), which is almost three times compared to that of Co and Mn, ca. 0.13*e*^−^ and 0.10*e*^−^, respectively. In the case of the Zn-doped CeO_2_ surface, this was found to be neutral and Zn/Ce was found to maintain almost their individual oxidation states the same. Also, no charge compensation occurs during the adsorption of CO molecules compared to the clean surface without the adsorbate.
(c) It was found that the strength of CO adsorption (or energy of CO chemisorption) for selected doped-ceria surfaces obtained by DFT calculations follows the order Cu > Co > Mn > Zn > CeO_2_. The same trend was found for the descriptor α, which expresses the extend of participation of the lattice oxygen in the CO oxidation reaction. Despite the structural similarity of the catalysts, the Cu–Ce–O surface was the one with the most populated labile O_lattice_. The latter participates, to the greatest extent, in the CO oxidation reaction. In Cu-doped ceria, more than 70% of the ^18^O exchanged participated in the reaction, whereas this percentage remains in the 40–50% range for the other dopants.

Also, the initial rate of CO oxidation with lattice oxygen (CO/Ar feed) when expressed in terms of TOF (s^–1^), based on the amount of oxygen corresponding to one monolayer for each TM-doped ceria solid, proved to be the highest for Cu-doped ceria, followed by Co-, Mn-, and Zn-doped ceria. This result nicely explained the activity order in terms of rate per gram of solid basis measured experimentally.

(d) By performing transient CO oxidation reaction experiments in the absence (CO/Ar) and in the presence of gaseous oxygen (CO/O_2_/Ar) and based on the initial rate of CO consumption (oxidation rate) and the total amount of CO consumed, it was obtained that lattice oxygen participated to the largest (∼70%) and to the lowest degree (∼25%) in the Cu-doped and Zn-doped ceria, respectively. These results demonstrate the importance of lattice oxygen in the CO/O_2_ reaction path and the validity of the MvK mechanism.
(e) In situ DRIFTS studies probed the difference among the solids to form carbonate species under reaction conditions, while SSITKA-DRIFTS studies proved that carbonates are likely intermediate species in the reaction over the most active Cu–Ce–O catalyst, leading to its deactivation.
(f) Kinetic analysis showed that the apparent activation energy (*E*_app_) presents very small differences among the different heteroatoms’ systems. However, a strong dependence of the pre-exponential factor (entropic contribution) was found on the heteroatom used, following the order Cu > Mn > Zn > pristine ceria, which coincides with the activity order. Hence, it is concluded that the order of reaction has stronger positive dependence on CO but not on O_2_ partial pressure.

## Supporting Information Available

Materials and methods along with results and discussion on basic characterization analysis (e.g., BET, H_2_-TPR, XPS, CO_2_-TPD, and isotopic oxygen exchange studies) (PDF)
