# Supplementary material for: Design Aspects of Doped CeO2 for Low-Temperature Catalytic CO Oxidation: Transient Kinetics and DFT Approach
Source: ACS Appl Mater Interfaces. 2021 Apr 9;13(19):22391–415. doi: 10.1021/acsami.1c02934 (PMC8153538; doi:10.1021/acsami.1c02934)
Supplement: Supplementary file 1 — am1c02934_si_001.pdf [file am1c02934_si_001.pdf]

SUPPORTING INFORMATION

Design Aspects of Doped-CeO_2 for Low-Temperature Catalytic CO Oxidation: Transient Kinetics and DFT Approach

Kyriaki Polychronopoulou[1,2,], Ayesha A. AlKhoori[1,2], Angelos M. Efstathiou[3], Maguy Abi Jaoude[2,4], C. Damaskinos[3], Mark A. Baker[5], Alia Almutawa[1], Dalaver H. Anjum[2,6], Michalis A. Vasiliades[3], Abderrezak Belabbes[2], Lourdes F. Vega[2,7], Abdallah Fathy Zedan[8], Steven J. Hinder[5]*

[1] Department of Mechanical Engineering, Khalifa University of Science and Technology, Main Campus, Abu Dhabi, UAE

[2] Center for Catalysis and Separations, Khalifa University of Science and Technology, Main Campus, Abu Dhabi, UAE

[3] Department of Chemistry, Heterogeneous Catalysis Lab, University of Cyprus, 1 University Ave., University Campus, 2109 Nicosia, Cyprus

[4] Department of Chemistry, Khalifa University of Science and Technology, Main Campus, Abu Dhabi, UAE

[5]The Surface Analysis Laboratory, Faculty of Engineering and Physical Sciences, University of Surrey, Guildford, GU2 4DL, UK

[6]Department of Physics, Khalifa University of Science and Technology, Main Campus, Abu Dhabi, UAE

[7] *Research and Innovation Center on CO2 and H2 (RICH), and Chemical Engineering Department, Khalifa University, Abu Dhabi, UAE*

[8]*National Institute of Laser Enhanced Science, Cairo University, Giza 12613, Egypt*

*Corresponding author: kyriaki.polychrono@ku.ac.ae

S1. MATERIALS AND METHODS

S1.1. TM doped-CeO_2 preparation

Synthesis example

$ZrOCl_2.8H_2O$ precursor salt was used to finally obtain Zr-Ce-O (Zr-doped CeO_2, solid solution). A citric acid solution was used as a complexing agent, and an aqueous solution was prepared in molar excess, maintaining the total metal loading to citric acid at 0.75 (M_{tot}/citric acid=0.75 mol/mol). The metal and citric acid solutions were then mixed and then heated in a microwave reactor (duration < 15 min) at mild conditions, ca. 130 °C and 800 W. Mixed metal oxides of previously mentioned materials were obtained through calcination in static air, ca. 500 ^{o}C for 6 h under atmospheric pressure. An analogous procedure was followed for all the other binary mixed metal oxides (TM-doped ceria).

S1.2. TM-doped CeO_2 materials characterization

To study the crystal structure of the calcined TM-doped CeO_2 solids, powder X-ray diffraction (XRD) was employed using a D2-Phaser® apparatus (Bruker, MA, USA) operated at the excitation wavelength of Cu *K*α (1.5418 Å), and a generator power at 30 kV and 20 mA. A scan range within 10 - 100° 2theta was applied with 0.02^{o} step size. Scherrer equation was employed to estimate the average crystallite size using the (111) peak broadening line of ceria. Williamson-Hall plots ($\beta cos\theta/\lambda$ *vs.* $sin\theta/\lambda$) were constructed and used to estimate the lattice strain.

High-resolution 3Flex Micromeritics (Atlanta, USA) adsorption apparatus with high-vacuum system and three 0.1 Torr pressure transducers was employed for textural measurements through the N_2 physisorption method at 77 K. A degassing process was carried out prior to the measurements to remove any residual moisture (degassing at 150 ^oC for 12 h) and weakly bound carbon dioxide. The BET surface area was calculated from the adsorption isotherm data in the relative pressure range of P/P_0=0.04-0.25. Information regarding the pore size distribution and specific pore volume (cm^3/g) were obtained after applying the Barrett-Joyner-Halenda (BJH) method[1].

The surface morphology of solid TM-doped CeO_2 catalysts was examined using the JSM 7610F-Field Emission Scanning Electron Microscope (JEOL Ltd., Tokyo, JPN), in both secondary (SE) and low-angle-backscattered imaging (BSE). H_2 temperature-programmed reduction (H_2-TPR) experiments were carried out in a conventional quartz reactor connected to a Thermal Conductivity Detector (TCD). The material was mounted on a U-shaped quartz tubular reactor (Autochem 2920, Micromeritics, Atlanta, USA). A gas stream of 10 vol% H_2/Ar was passed over 0.2 g of the pre-calcined sample (20 vol.% O_2/He, 500 ^oC, 2 h) at the flow rate of 30 NmL/min. The temperature was raised at 30 ^oC/min from room T to 500 ^oC and kept at this temperature for 2 h, while the TCD signal was recorded continuously.

CO_2-TPD experiments were conducted using Autochem 2920 (Micromeritics, Atlanta, USA). The materials were mounted on a U-shaped quartz sample tube and placed in a flow-through reactor. A gas flow of 5 vol% CO_2/Ar (30 NmL/min) was passed over 0.2 g of the pre-calcined (20 vol%O_2/He, 500 ^oC, 2 h) sample for 30 min. The temperature of the sample was then increased at the heating rate of 30 ^oC/min to 500 ^oC and kept there for 2 h, while the TCD signal was recorded continuously.

Information regarding surface elemental composition and chemical states were obtained using X-ray Photoelectron Spectroscopy (XPS, Thermo-Fisher Scientific East Grinstead, UK, Theta Probe spectrometer). A monochromatic Al Kα X-ray source (hν = 1486.6 eV) and an X-ray spot of radius ~ 400 μm were employed to record XPS spectra for the calcined materials. Survey and high-resolution spectra were acquired using pass energies of 300 eV and 50 eV, respectively. C 1s peak (285.0 eV) was taken into consideration in all spectra to account for charging effects during

acquisition, and correct binding energies, accordingly. The non-linear (Shirley) background was eliminated from all spectra for the purpose of obtaining high resolution core level spectra for quantitative surface chemical analyses. The experiment was coupled with the manufacturer's Avantage software that acquires the appropriate sensitivity correction factors for the electron energy analyzer transmission function.

HR-TEM studies were performed over the solids with the use of an electron microscope (model Titan 80-300 ST, operated at 300 kV). The microscope was equipped with a spherical aberration (Cs) corrector for the image (CEOS CETCOR), and an energy filter (model GIF Quantum 963, Gatan, Inc.). Selected powdered specimens were dispersed in ethanol and drop casted onto carbon-coated copper grid (200 mesh) to perform imaging, structure, composition, and elemental mapping analyses. The high-resolution imaging was performed in aberration corrected TEM mode, while the structure analysis was carried out using the selected area electron diffraction (SAED) technique of TEM. The composition of samples was determined using X-ray energy dispersive spectroscopy (EDX). However, nanoscale elemental quantification was carried out using scanning TEM (STEM) mode. For the elemental quantification, STEM-EDX experiments were performed by setting the same electron beam conditions for the specimens of each sample. The entire TEM data acquisition and post processing was done by utilizing Gatan Microscopy Suite (GMS, version 3.2).

S1.3. Catalytic CO oxidation performance studies

The catalytic oxidation of carbon monoxide over the various TM-doped CeO_2 materials was carried out in a differential packed-bed quartz U-tube flow reactor (i.d. = 9 mm) mounted in the middle of a programmable split tube furnace (Lindberg/Blue M Mini-Mite Tube Furnace - Thermo). A catalyst sample of 50 mg was packed between two thin layers of glass wool. Thermocouples (K-type) were located at the inlet and outlet of the catalyst bed for gas-phase temperature monitoring, very close to that at the external surface of catalyst grains (powder form). The signal from the thermocouple was acquired using USB-6008 Multifunction I/O and NI-DAQmx (National Instruments) data acquisition system. The composition of the exit gas stream from the micro-reactor was analyzed using an infrared gas analyzer (IR200, Yokogawa). The feed gas mixture consisted of 4 vol% CO, 20 vol% O_2 and He as balance gas. Unless otherwise

mentioned, regulated gas mixture total flow – (use of digital thermal mass flow controllers (HI-TEC Model F-201CV-10K-AGD-22-V Multi-Bus DMFC; Bronkhorst) was set at 50 mL/min (STP) corresponding to a space velocity (SV) of ~ 60,000 cm^3 g_{cat}^{-1} h^{-1}. After stabilization, the temperature of the catalyst bed was raised at 5 ^oC min^{-1} in the flow of $CO/O_2/He$ gas mixture. Labview-configured data acquisition software was used to record and store the vol% composition in CO, CO_2 and O_2 alongside the temperature throughout the CO activity vs T procedure. Further catalytic stability experiments were conducted on selected catalysts under continuous feed gas stream, ca. 20 h at 530 ^oC.

S1.4. DFT Calculations

The all-electron wave functions and the pseudo-potentials for the electron-ion interactions are described within the projector-augmented wave (PAW)[2] method. The exchange and correlation (XC) potentials were generated within the generalized gradient approximation (GGA) scheme according to Perdew, Burke and Ernzerhof (PBE)[3]. The Kohn-Sham wave functions were expanded in plane waves up to an energy cut-off of 50 Ryd, while the cut-off for the electron density representation was set to 300 Ry. This ensures the energy convergence within 1 mRy per atom. For a proper description of the atomic and electronic structures of chemisorbed CO on the TMs doped-CeO_2 surface, it is necessary to account for the strong electron-electron correlation of the highly localized *f* states. We applied a DFT+*U* scheme[4] with an additional interatomic Coulomb repulsion term, U= 5 eV. This value usually provides a good description of the electronic structure of CeO_2 surface and its oxidation states[4]. A 6x6x1 Monkhorst-Pack mesh[5] was used to sample the corresponding Brillouin zone (BZ). In order to simulate the doped-CeO_2 surfaces with two-dimensional translational symmetry, the repeated slab method was used. For a low heteroatom coverage of TMs atoms, we used slabs of nine CeO_2 layers with a lateral $p(2\times2)$ surface unit cell. A vacuum thickness corresponding to nine layers (15 Å) was chosen to separate the two surface slabs and to avoid artificial interactions between them. The atoms in the top six layers were allowed to fully relax until the forces become smaller than 1 meV/Å, while the remaining layers at the bottom of the slab were kept fixed. The optimized lattice parameter of bulk CeO_2, based on the above computational settings, was a=5.431 Å. This is in good agreement with measurements and previous calculations reported[6].

S2. RESULTS AND DISCUSSION

S2.1. Structural, textural and morphological studies on TM-doped CeO_2 materials

S2.1.1. Textural properties

The N_2 adsorption/desorption isotherms recorded at 77 K for the Mn-Ce-O catalyst are presented in **Fig. S1C**, whereas specific surface area values are listed in Table 1. All catalysts demonstrated type-IV adsorption isotherm according to IUPAC classification, which is associated to capillary condensation occurring due to the mesoporous (pore width ~ 2-50 nm) nature of the solid catalysts. A hysteresis type-H3 is identified in all materials at ~ P/Po > 0.45 through the steep trend of the desorption curve towards the closure point, as well as the infinite vertical behavior of the adsorption curve towards high P/Po values (~ 0.99), which is indicative of the macropores nature (pore width > 50 nm) of the materials. This is in agreement with SEM images (**Fig. S1B**)[7]. Usually, adsorption hysteresis is related to specific shaped pores, and H3-type indicates that the synthesized catalysts acquire a slit-shaped pores[7]. The low N_2 uptake at a low relative pressure is observed in all the mixed metal-oxides due to the lack of micropores (pore width < 2 nm). The surface area and cumulative specific pore volume were obtained through the BET and Barret-Joyner-Halenda (BJH) methods[8], respectively.

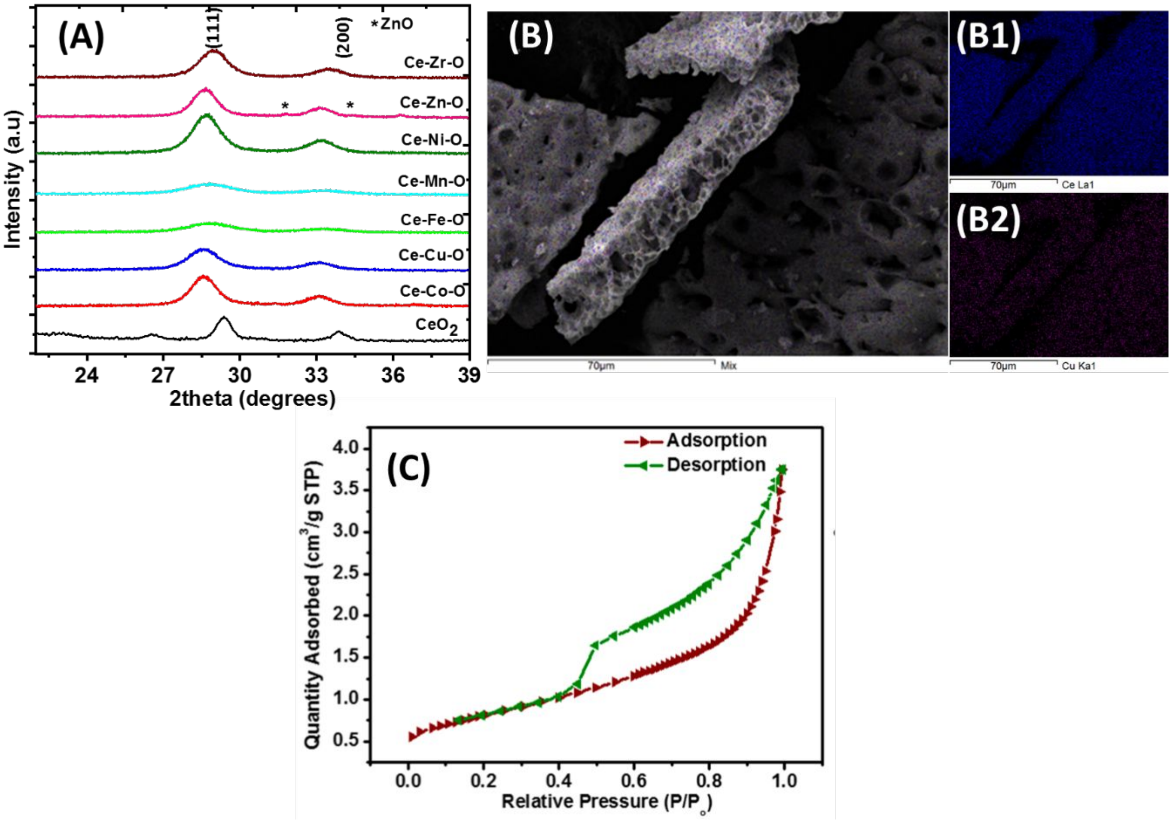

Figure S1: (A) Powder XRD patterns of the various TM-doped ceria catalysts in the 23-39 2theta range. **(B)** SEM photos of the Cu-Ce-O catalyst along with the elemental mapping (B1, B2). **(C)** N_2 adsorption-desorption isotherms obtained at 77 K over the Cu-Ce-O catalyst.

Variations in the BET area as well as pore volume are observed due to dopant-type effect (Table 1). BET area values for the mixed metal oxides varied in the 12 - 63 m^2/g range. The BET area of ceria (14.8 m^2/g) is enhanced after doping with particular transition metals (i.e. Mn, Fe and Cu), whereas for the other TM-dopants, no improvement is observed. Mn-Ce-O solid exhibited the highest BET area (63 m^2/g) as well as specific total pore volume (0.130 cm^3/g). A reason for this might be its small crystallite size as confirmed by the powder XRD results (**Fig. S1A**), where it is expected that smaller particles exhibit higher specific surface area. On the other hand, $Ce_{0.8}M_{0.2}O_{2-\delta}$ (i.e. Zn, Ni, Co) possessed lower BET area and cumulative pore volume. This explanation, however, does not apply for Cu and Fe mixed metal oxide systems. Cu-Ce-O had a BET area ~ 16.2 m^2/g – among the lowest- but acquired the second highest total pore volume, ca. ~ 0.085 cm^3/g. This could be attributed to the highly dense macro/mesoporous particles.

Zr^{4+}-doping of ceria improved significantly the surface area of pristine ceria from 14.8 to 61.2 m^2/g (Zr-Ce-O). This result agrees with previous findings and indicates that Zr^{4+} incorporation in ceria enhances the resistance of ceria against surface area drop under thermal treatment in high temperatures (627-827 °C). All mixed metal oxides demonstrated wide distribution of macro/mesopores induced by microwave synthesis.

S2.1.2. Structural properties

The X-ray diffraction pattern of Zr-Ce-O *at a first glance* confirmed the formation of a fluorite structured solid solution, with a lattice parameter of 5.69 Å compared to 5.82 Å of pure ceria. The ionic radius of Zr^{4+} (0.80 Å) is smaller than that of Ce^{4+} (0.97 Å), which causes shrinkage of the unit cell after Zr^{4+} incorporation (decrease in the lattice parameter). This finding is in agreement with a previous study conducted on CeO_2-ZrO_2 with similar composition[9]. The crystallite size obtained for Zr-Ce-O prepared by the microwave synthesis is smaller than that of similar composition obtained by the coprecipitation method (10 and 13 nm, respectively)[10].

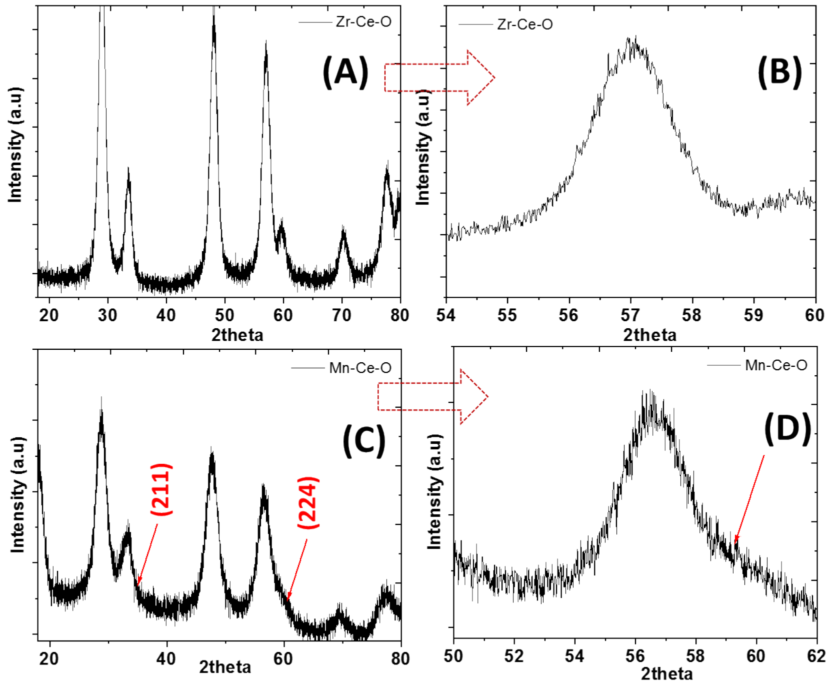

Figure S2: Powder XRD patterns of **(A)** Zr-Ce-O, **(B)** Zoom-in of (A) in the 55-60 2theta range, **(C)** Mn-Ce-O, and **(D)** the (C) in the 50-60 2theta range.

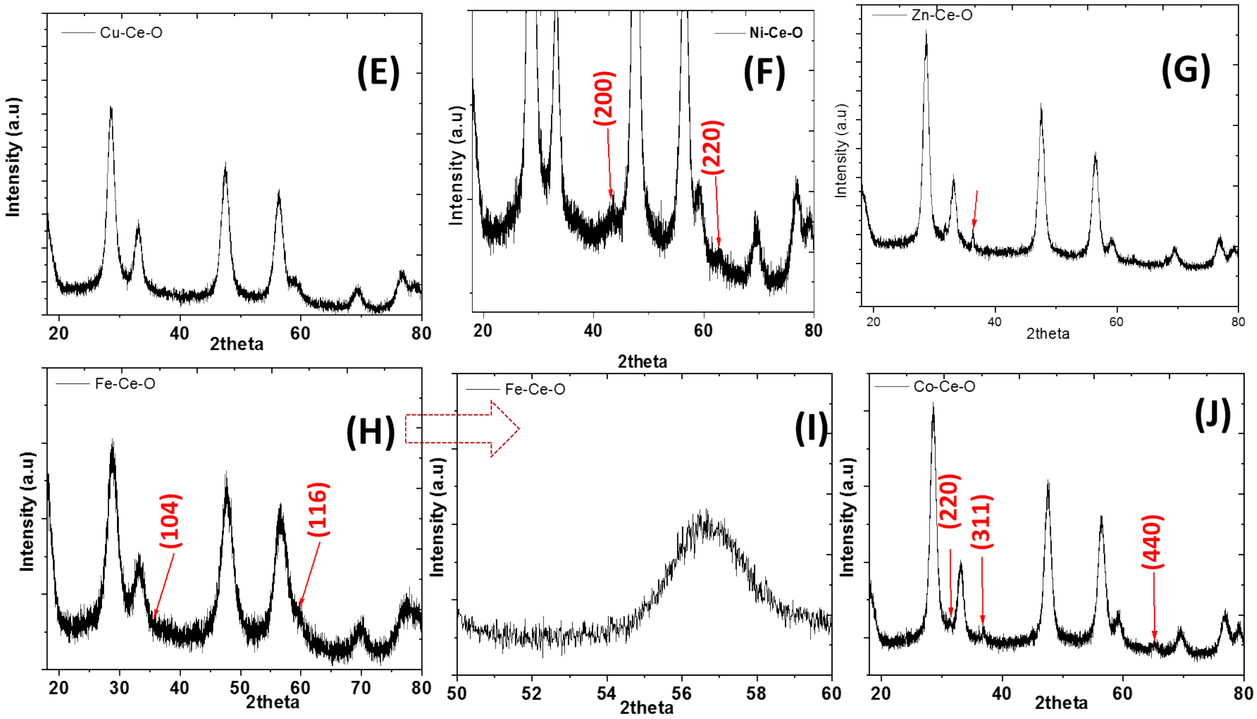

Figure S3: Powder XRD pattern of **(E)** Cu-Ce-O, **(F)** Ni-Ce-O, **(G)** Zn-Ce-O, **(H)** Fe-Ce-o, **(I)** Fe-Ce-O, and **(J)** Co-Ce-O doped-ceria materials.

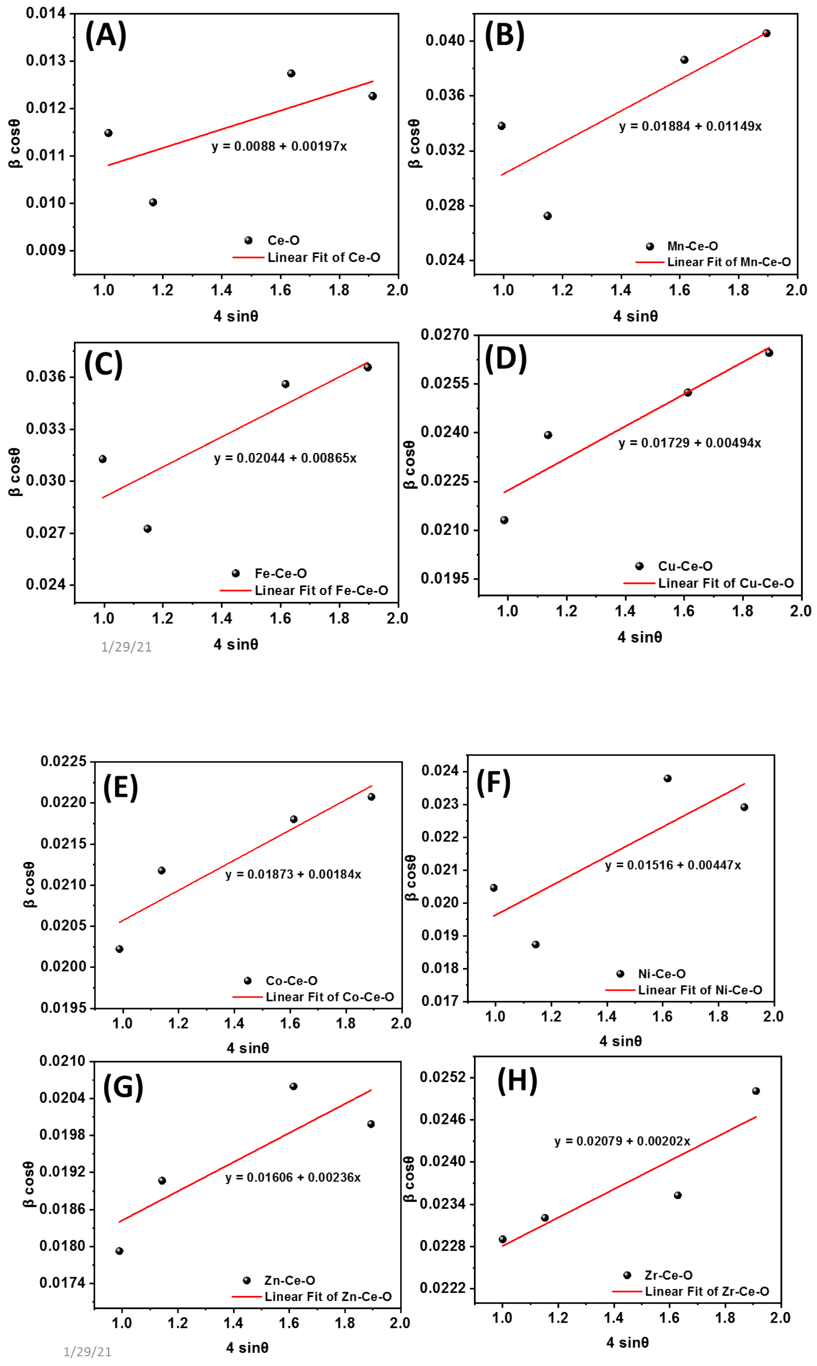

Figure S4: Williamson-Hall plots related to **(A)** Co-Ce-O, **(B)** Mn-Ce-O, **(C)** Fe-Ce-O, **(D)** Cu-Ce-O, **(E)** Co-Ce-O, **(F)** Ni-Ce-O, **(G)** Zn-Ce-O, and **(H)** Zr-Ce-O solids.

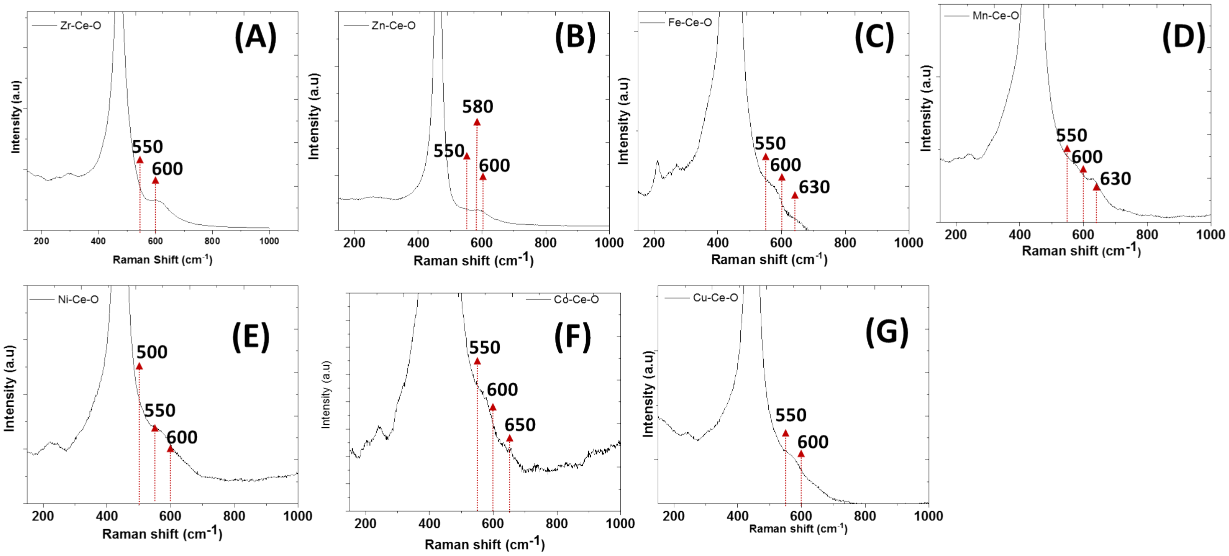

Figure S5: Raman spectra of **(A)** Zr-Ce-O, **(B)** Zn-Ce-O, **(C)** Fe-Ce-O, **(D)** Mn-Ce-O, **(E)** Ni-Ce-O, **(F)** Co-Ce-O, and **(G)** Cu-Ce-O solids.

Table S1. Literature overview about the formation of solid solution for the doped-ceria solids.

**Dopant**	**Content (at.%)**	**Formula**	**Synthesis Method**	**Calcination conditions (°C/h)**	**Dopant oxide**	**Reference**
Cu, Cr, Co, Zn, Ni, Mn, Fe, Zr	10	$Ce_{0.9}M_{0.1}O_{2-\delta}$		800°C/ 4 h	No	(11)
Mn, Fe, Zr and Pr, La, or Sm	20-30	$Ce_{1-x}TM_xO_{2-\delta}$ and $Ce_{1-x}RE_xO_{2-\delta}$	Co-precipitation	773 and 1073 K/ 5 h	Only in the case of Mn-dopant (Mn_3O_4)	(12)
Mn	(5-30)	$Ce_{1-x}Mn_xO_{2-\delta}$	Reverse homogeneous precipitation	1300°C	Mn_3O_4	(13)

Mn, Fe and La, Pr	20%	$Ce_{0.8}M_{0.2}O_{2-\delta}$	Sol-gel	773K/5h	No	(14)
Cu,	20	-	-	-	No	(15)
Cu	5-10	CuO_x-CeO_2	Sol-gel	500°C/4h	No	(16)
Ni, Cu, Co, Mn, Ti, Zr	10	$Ce_{1-x}M_xO_2$	Co-precipitation	400°C for 2 h and 700-1000 C for 6 h	No	(17)
Fe, Mn, Zr, La, Pr, Hf	20	-	Co-precipitation	773 K for 5 h	No	(18)
Mn	30	$Ce_{0.7}Mn_{0.3}O_{2-\delta}$	Co-precipitation	873, 973, and 1073K/5h	Mn_2O_3@1073K	(19)
Cu, Co, Ni, Mn	10	-	solvothermal	-	No	(20)

Table S2. Characteristic Raman bands of the pure dopant metal oxides investigated in this study.

Dopant Oxide	Raman Bands (cm^{-1})	References
ZnO	275, 437, 580	(22-26)
Fe_2O_3	200, 300, 613	(22-26)
Co_3O_4	282, 330, 616	(22-26)
Mn_3O_4	308, 353, 647	(22-26)
NiO	500	(22-26)

S2.1.3. Elastic energy upon doping of ceria:

The elastic energy is given by the following formula:

$$W = 6Gd_o\Delta d^2 \quad (1)$$

where G is the shear Modulus, and d_o is the lattice parameter for the host oxide (CeO_2). G value of 92.5 GPa was used for CeO_2 [21]. The elastic energy values for the Cu, Co, Ni, Zn, Fe, Mn were found to be 8.4, 5.2, 4.9, 6.5, 7.1 and 5.2 ($x10^{-9}$) N-Å.

S2.2. Oxidation states of TM-dopants in doped-CeO_2 catalytic materials

The oxidation states of transition metals on the surface of TM-doped CeO_2 were identified based on the core level binding energy signal and the presence or absence of shake-up satellites in the XPS spectra. The Cu $2p_{3/2}$ peak is observed at ~ 933.4 eV, and there is a broad shake-up satellite at a binding energy of 941-944 eV (**Fig. S6E**). The satellite peak is observed only for Cu^{2+} and a binding energy of 933.7 ± 0.1 eV is expected for CuO, and 932.3 ± 0.1 eV for Cu_2O. Hence, Cu atoms predominantly exist within the mixed metal oxide (solid solution formation) in the 2+ oxidation state[27].

The Co $2p_{3/2}$ peak (**Fig. S6F**), occurs at a binding energy of 780.2 eV and there is a satellite peak at ~785 eV. These peak positions and the spectral shape recorded are in very good agreement with that of CoO reference material than the mixed oxidation states of Co_3O_4[28], demonstrating the predominance of Co^{2+} oxidation state in the present mixed metal oxide Co-Ce-O solid composition. This finding also indicates that Co^{2+} species are more populated than the other possible oxidation states, Co^0 or Co^{3+} [5]. However, an overlap between CoO, Co_3O_4, Co_2O_3 and CoOOH exists at the same binding energy. Interestingly, it has been reported that Cu^+ and Co^{2+} species are preferred over Cu^{2+} and Co^{3+}, respectively, during CO oxidation reaction[29, 30]. This finding was correlated with the CO conversion performance of Cu-Ce-O and Co-Ce-O catalysts.

The Ce3d region (**Fig. S6A**) is a typical Ce 3d spectrum, as it is discussed in the main manuscript, where the 4+ valence state is predominant, with little evidence for 3+ cerium oxidation state, indicative of almost a fully oxidized CeO_2. The Fe 2p region (not shown) is rather complicated by the presence of two Ce Auger peaks ($M_4N_{4,5}N_{6,7}$ at ~716 eV and $M_5N_{4,5}N_{6,7}$ at ~732 eV). The Fe $2p_{3/2}$ and Fe $2p_{1/2}$ peaks occur at the binding energies of 710.9 and 724.2 eV. These binding energies agree very well with Fe in the Fe^{3+} oxidation state[31].

The Mn 2p spectra show the Mn $2p_{3/2}$ peak at a binding energy of 641.7 eV, and a broad shake-up satellite peak at ~ 645 eV (**Fig. S6D**). The peak/satellite binding energies and shape are in best

agreement with MnO (in comparison to other manganese oxides)[32]. Hence, Mn^{2+} is the predominant oxidation state for this dopant in the Mn-doped CeO_2 structure.

In **Fig. S6C**, the Ni $2p_{3/2}$ peak occurs at a binding energy of 855.0 eV. This binding energy is typical of Ni^{2+} but the observed peak shape does not agree very well with either NiO or $Ni(OH)_2$ and the binding energy lies mid-way between that of NiO (854.7 eV) and $Ni(OH)_2$ (855.3 eV)[33]. Thus, in the Ni-Ce-O mixed metal oxide, the Ni is predominantly present as Ni^{2+}, but the XPS Ni 2p peak shape observed does not clearly correspond with that of NiO due to the presence of $Ni(OH)_2$ at the surface which can be due to the atmosphere exposure of the Ni containing catalyst.

Finally, Zn exists only in its 2+ state when oxidized. The Zn $2p_{3/2}$ peak (**Fig. S6B**) shows a sharp peak at a binding energy of 1021.9 eV, which is in excellent agreement with ZnO recorded by Klein and Hercules[34].

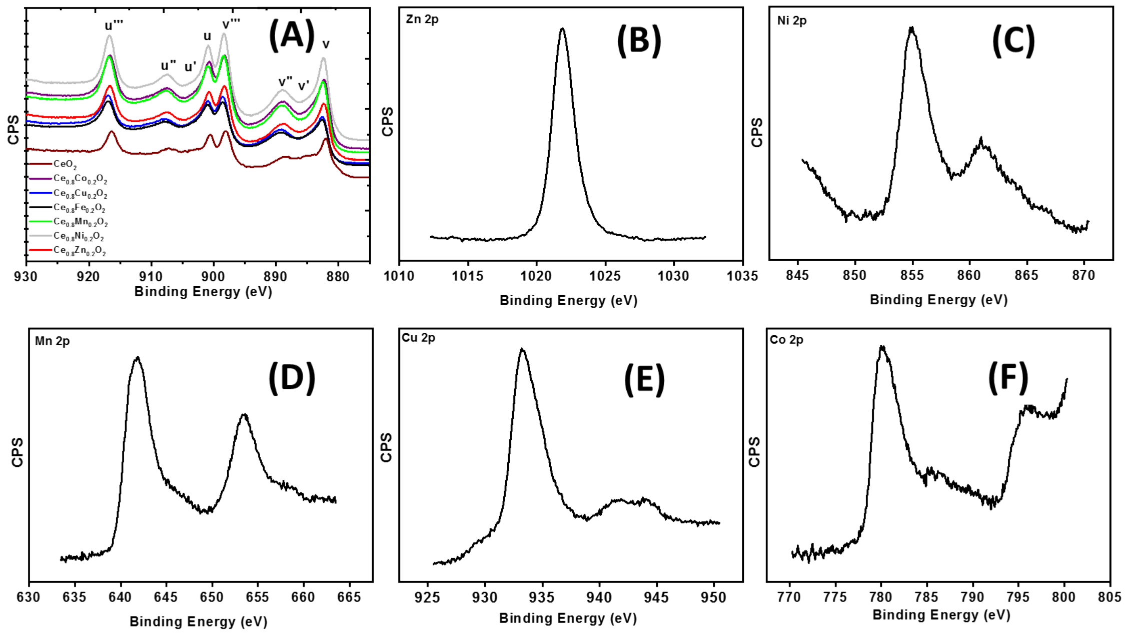

Figure S6: XPS high resolution spectra of Ce3d **(A)**, Zn 2p **(B)**, Ni 2p **(C)**, Mn 2p **(D)**, Cu 2p **(E)**, and Co 2p **(F)**.

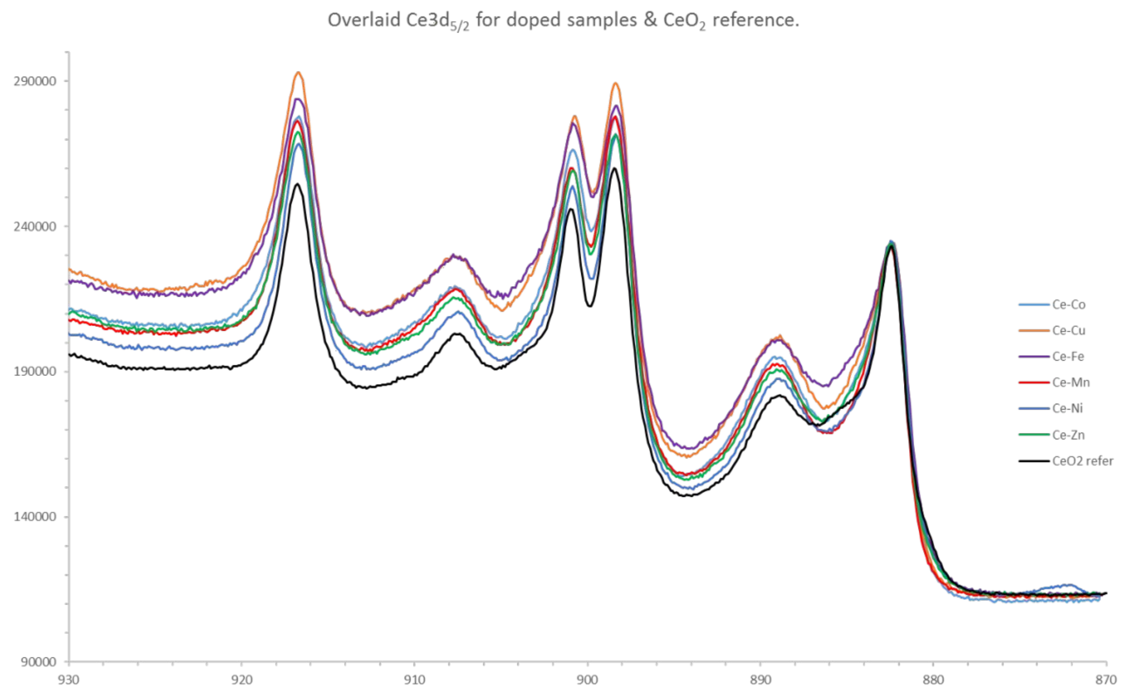

Figure S7: Overlaid Ce3d5/2 for doped samples and CeO2 reference

Table S3: Binding energies (eV) and corresponding oxidation states of TM - dopants in the doped-CeO_2 catalytic materials after calcination at 500 ^oC.

**Material ($Ce_{0.8}M_{0.2}O_2$)**	**Binding Energy (eV)**	**Oxidation States**
Ce-Cu-O	933	Cu^{2+}
Ce-Co-O	780	Co^{2+}
Ce-Fe-O	709, 711	Fe^{2+}, Fe^{3+}
Ce-Mn-O	641	Mn^{4+}
Ce-Ni-O	853.8, 858.8	Ni^{2+}/Ni^{3+}
Ce-Zn-O	495	Zn^{2+}

S2.3. Redox properties

H_2-TPR was employed to investigate the effect of the nature of dopant (size, oxidation state) on the reducibility of M-Ce-O catalysts. H_2-TPR profiles shown in **Fig. 4C** and **Fig. S8A, B** suggest that the variations in the reduction behavior of the binary metal oxide systems are due to dopant-type as well as to the different dopant cation oxidation state (i.e. divalent, trivalent, and quadrivalent). According to the literature, pure ceria experiences two reduction peaks both appearing at temperatures higher than 350 °C[35,36]. In pure ceria, an initial reduction peak at ~ 485 °C is attributed to surface shell reduction, and a high temperature reduction peak at ~ 765 °C is attributed to bulk material reduction[37,38]. In microwave-assisted prepared ceria, the reduction peaks are observed at ~ 247 °C (sub-surface), 467 °C and 1000 °C (bulk)[39]. The preparation route has been known to affect the reduction properties of ceria-based catalysts[40,41].

Divalent cations: **Fig. 4C** shows the effect of divalent cations, Co^{2+}, Cu^{2+}, Ni^{2+} and Zn^{2+} on the doped-ceria reducibility. The Cu-Ce-O reduction profile presents an intense peak at very low temperatures, at ~ 200 °C, with a shoulder peak at around 100 °C that extends up to 150 °C. These peaks correspond to the stepwise reduction of CuO, for example, Cu^{2+} to Cu^{+}, and Cu^{+} to Cu^{0}[42]. The broader narrow peak above 400 °C is attributed to the reduction of the remaining Ce^{4+} [42]. CuO has been reported to have one reduction peak at ~ 300°C[43], where Cu-Ce-O $Ce_{0.8}Cu_{0.2}O_{2-\delta}$ is reduced at lower temperatures than both single oxides of CeO_2 and CuO, demonstrating the *successful synergy* achieved by introducing Cu^{2+} into the CeO_2 lattice. From the synthesis perspective, it can be stated that microwave synthesis enhanced the homogeneous dispersion of Cu in the ceria lattice, since the low reduction temperature peak ~ 100°C is absent for the same composition prepared via the co-precipitation method due to agglomeration. One main reduction peak also appears at ~ 250 °C[44]. Another study on 10 wt% Cu-doped ceria[35] reported the first (lowest temperature) reduction peak to occur at 245, 230 and 190 °C, respectively, for the same solid composition prepared via three different routes, namely, deposition-precipitation, co-precipitation, and complexation-combustion. The reduction of ceria surface is promoted with copper addition[35], and with the combined effect of microwave-assisted synthesis, the reduction occurs at clearly lower temperatures. The high reducibility shown by Cu-Ce-O can be correlated to the powder XRD results, which confirmed the formation of solid solution, thus, improved interaction between Cu and Ce cations in the crystal lattice structure.

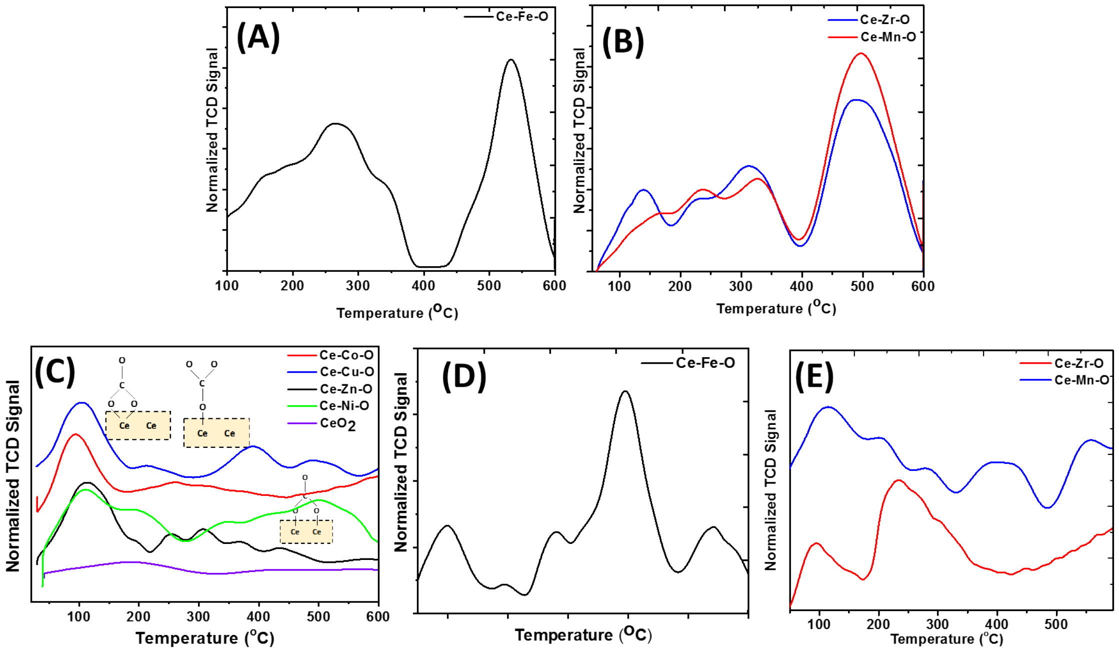

Figure S8: H_2-TPR profiles **(A-B)** of ceria doped with trivalent (A) and quadrivalent (B) TM dopants. CO_2-TPD profiles of ceria doped with divalent **(C)**, trivalent **(D)** and quadrivalent **(E)** TM dopants.

On the contrary, Zn-Ce-O reduction profile (**Fig. S9**) is appeared rather suppressed (in terms of intensity), with Zn^{2+} species being relative inactive, even though they are highly populated on the surface (as confirmed by XPS). Therefore, it can be concluded that the hydrogen reduction peaks are mainly due to the reduction of Ce^{4+} species, where ZnO presence confirmed by XRD, contributes to the enhancement of the synergy with the mixed metal oxide which shifts the reduction peaks towards lower temperatures compared to those of pure ceria, in agreement with the literature[45,46]. It was reported that ZnO is not easily reducible, though Zn^{2+} reduction can be facilitated when it is adjacent to a metal cation by a synergistic effect between the mixed metal cations[46].

Co-Ce-O TPR profile demonstrates the stepwise reduction processes involved due to the highly dispersed cobalt species in ceria **(Fig. 4C & Fig. S9).** The addition of Co greatly boosts the surface reduction of Ce^{4+} as indicated by the peak at 300 °C, whereas the formation of $Ce_{0.8}Co_{0.2}O_{2-\delta}$ solid

solution implies that $Co^{3+}/Co^{2+}/Co$ and Ce^{4+}/Ce^{3+} reduction processes can also take place. Namely, (a) reduction of surface oxygen and Ce^{4+}, (b) Co^{3+} to Co^{2+}, (c) Co^{2+} to Co and (d) reduction of surface ceria species (~ 545°C). This observation is in agreement with previous reported literature on cobalt-doped ceria[37].

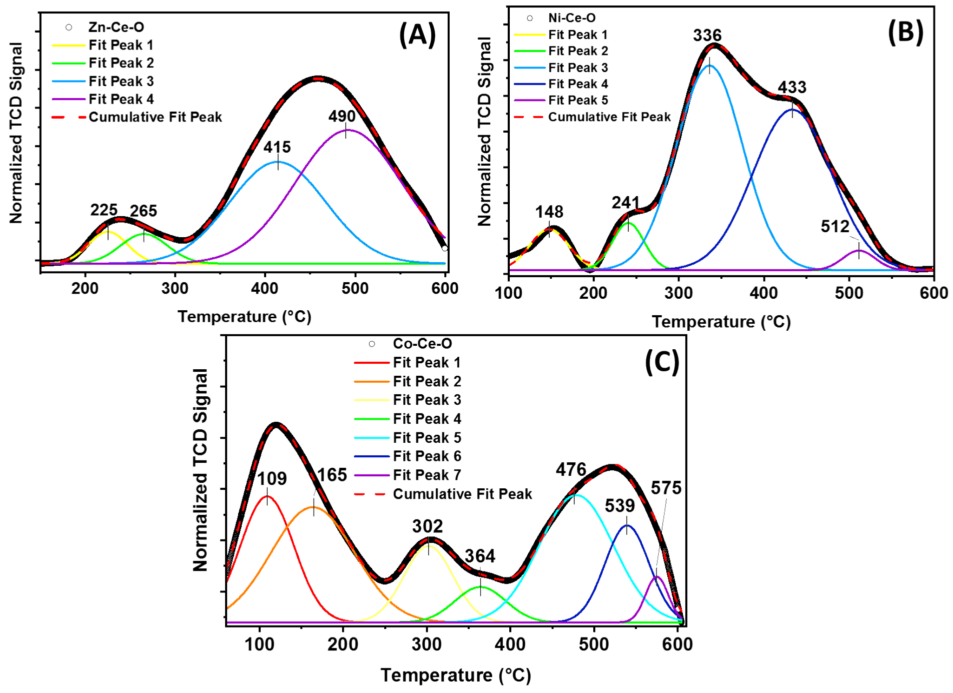

Figure S9: Deconvoluted H_2-TPR profiles of the Zn-Ce-O, Ni-Ce-O and Co-Ce-O solids.

H_2-TPR reduction profile of $Ce_{0.8}Ni_{0.2}O_{2-\delta}$ shown in **Fig. 4C** and **Fig. S9** exhibits peaks that can be assigned according to the following. Pure NiO only shows one reduction peak at ~ 400°C, which corresponds to reduction of NiO to metallic Ni^0 [47]. Upon introduction in the ceria lattice, lower temperature peaks arise due to strong interactions between ceria and NiO. The low reduction peaks at ~ 155 and 235°C are attributed to reduction of ceria surface oxygen species. The presence of surface-active oxygen vacancies in the Ni-Ce-O solid solution (as confirmed by XRD, Raman) can be explained by Ni^{2+} having lower cation charge than Ce^{4+} which causes lattice distortion[47,48]. The high temperature peak observed at 380-500 °C corresponds to the simultaneous reduction of NiO to Ni^0 and remaining Ce^{4+}. Microwave-assisted synthesized Ni-Ce-O presented more mobile oxygen species than other similar in composition NiO-CeO_2 catalysts prepared by co-precipitation or sol-gel methods. In fact, the conventional methods reported reduction peaks for NiO-CeO_2

similar to that of pure NiO[49,50]. Thus, it can be concluded that Microwave synthesis strengthens the interaction between NiO and CeO_2 that allows reduction to commence at lower temperatures.

Trivalent Cations: **Fig. S8A** presents the H_2-TPR profiles of the rest of the catalysts. According to previous studies, bare Fe_2O_3 experiences three reduction peaks at 373, 590 and 724 °C. The first hydrogen consumption peak is attributed to the reduction of Fe_2O_3 to Fe_3O_4[51]. The other two peaks correspond to the stepwise reduction of Fe_3O_4 to intermediate FeO and metallic iron (i.e. Fe^0) [51,52]. In the case of Fe-Ce-O, an overlap between the reduction peaks of pure ceria as well as those of Fe_2O_3 is expected. The H_2-TPR trace (**Fig. S8A**) shows the first low temperature peak at 265 °C. This peak is attributed to the reduction of surface Fe^{3+} and Ce^{4+} species. This behavior is reported also on previous studies on Fe-doped ceria[52]. The peak is shifted towards lower temperatures due to the synergy between the cation and pure ceria through solid solution formation (confirmed by XRD and Raman), which facilitates the reducibility of the mixed oxide as explained above. The high temperature peak is observed in the 450-600 °C range, which corresponds to reduction of Ce^{4+} as well as of Fe_3O_4 to FeO. The H_2-TPR trace obtained for the microwave-assisted synthesized Fe-Ce-O shows resemblance to other studies on Fe-doped ceria[46]. However, a shift in the low-temperature peak is favored in microwave synthesized Fe-doped ceria compared to other traditional methods. The first reduction peak is observed at 384 °C for a sol-gel prepared 10 mol% Fe (~ 20 wt%) in ceria[46], whereas microwave-assisted prepared $Ce_{0.8}Fe_{0.2}O_{2-\delta}$ provides the same peak at 265 °C. This behavior can be explained by taking into account that microwave synthesis enhances the dispersion of dopant cation on the surface, or/and promotes the formation of oxygen vacancies (confirmed by Raman) by allowing Fe^{3+} to substitute for Ce^{4+}, which introduces charge compensation.

Quadrivalent Cations: Zr-Ce-O TPR profile features two main asymmetric reduction peaks in the 100-400 °C range and at ~ 580 °C (**Fig. S8B**). The peak asymmetry indicates the overlap between surface and bulk reduction of Ce^{4+} species. The reduction peaks of microwave prepared $Ce_{0.8}Zr_{0.2}O_{2-\delta}$ are shifted towards lower temperatures compared to those prepared via the coprecipitation method, according to Lan Li et al. [53], who reported asymmetric TPR consumption peak at ~ 650°C. Zr-Ce-O TPR profile maintains the feature of CeO_2 with slight variation in peaks

intensity and position. The first peak corresponds to reduction of the accessible Ce^{4+} species, whereas the second peak corresponds to oxygen removal from the bulk[54].

The H_2-TPR profile of Mn-doped CeO_2 (**Fig. S8B**) shows reduction peaks at ~ 100, 180, 390 and 500 °C. The first peak could be referred to the reduction of highly dispersed MnO_2/Mn_2O_3 to Mn_3O_4, and the second peak can be assigned to the reduction of Mn_3O_4 to MnO and surface Ce^{4+} species. The third peak is associated with the reduction of remaining ceria and manganese oxides[55]. The observed peaks are shifted towards lower temperatures compared to CeO_2, which implies a synergistic interaction between ceria and manganese through the solid solution formation (confirmed by XRD). The latter promotes surface reduction of Mn-Ce-O. This result is in agreement with previous findings on Mn-doped ceria[55-57]. Moreover, the reduction peaks are shifted towards lower temperatures due to the catalyst preparation method effect (microwave-assisted synthesis). The first and second reduction peaks for 30 wt% Mn in ceria, prepared by the co-precipitation method, were found to be located at 315 and 410 °C[58]. This observation emphasizes the role of microwave-assisted synthesis in enhancing the reduction properties of ceria-based mixed metal oxides system.

S2.4. CO_2-Temperature Programmed Desorption (CO_2-TPD) on TM-doped CeO_2

CO_2-TPD traces were obtained over the mixed metal oxides in order to study the surface-CO_2 interaction characterizing their surface basicity (**Fig. S8C**). As CO_2 is an acid molecule, it is expected to interact with basic surface sites[59].

Divalent ions: **Fig. S8C** shows that all the mixed metal oxides demonstrate an initial CO_2 desorption peak at ~ 100°C with a higher intensity than that of pure ceria. This observation indicates that transition metal doping of ceria strengthens the Lewis-type surface basic cites of ceria at the low-temperature region (50-150°C). The initial desorption peak intensity of all divalent TM doped ceria was independent of dopant chemical nature. The desorption peaks at intermediate desorption temperatures varied in terms of intensity. This finding indicates that dopant chemical nature has an influence on the surface basic nature of the catalyst altering the intermediate and strong basic sites. Zn-Ce-O tends to have strong basic surface character as the CO_2 desorption peak is in the 200-450 °C range. On the other hand, Cu-Ce-O tends to have higher surface basicity at

temperatures higher than 400 °C. The tendency of the surface basicity of mixed metal oxides throughout the temperature range is *highly correlated with their CO conversion activity behavior*.

CO_2-TPD trace of Ni-Ce-O **(Fig. S8C)** shows the highest surface basicity compared to the other TM-doped ceria solids. CO_2 desorption starts at temperatures slightly below 100°C, which indicates basic centers enrichment of Ni-doped ceria. Similar CO_2-TPD trace at low temperatures was obtained for the NiO-CeO_2 system prepared by the wet impregnation method[60]. However, with the present microwave-assisted synthesis, the basic centers are extended up to higher temperatures (400-600 °C), indicating a continuous desorption of CO_2. Previous work conducted on NiO-CeO_2 prepared by molten-salt method showed the same CO_2-TPD behavior as the one presented here. Mn^{2+}-doping showed great modification of the basic centers of pure ceria at high temperatures. CO_2-TPD trace of Mn-Ce-O **(Fig. S8E)** shows four desorption peaks with overlapping at 140, 250, 400 and 500 °C, which is in agreement with data reported in the literature [61].

Trivalent ions: Pure iron (III) oxide is considered basic due to its metal ion electronegativity (El(Fe^{3+})= 12.6) [62] and according to the acid - base classification reported by Tanabe et al.. Doping of ceria has higher basic nature (e.g., El(Ce^{4+})=9.9)[62] and it is expected to increase the basicity of the mixed metal oxide system. According to the literature[62], Fe_2O_3 displays complex CO_2-TPD trace with multiple desorption peaks **(Fig. S8D).** The most intense desorption peak is observed at low temperatures, ~ 70°C, whereas additional weak desorption peaks are observed at 250, 490 and 650 °C. **Fig. S8D** shows that doping of ceria with Fe^{3+} causes the CO_2-TPD trace to broaden over a wider temperature range compared to pure CeO_2 **(Fig. S8C)**. It is noticed that the low desorption peak is maintained at similar intensity, however, the intensity of the high-temperature peaks is increased (350-450 °C). This finding reflects the wide heterogeneity of basic centers developed for Fe-Ce-O catalyst.

Quadrivalent ions: Modification of the basic sites of ceria was also observed with quadrivalent cation doping (e.g., Zr^{4+}) as shown in **Fig. S8E**. The CO_2-TPD trace recorded on Zr-Ce-O shows characteristic desorption peaks of ZrO_2, which indicates alteration in the basicity of ceria with Zr^{4+} incorporation. Based on the literature[63,64], bare ZrO_2 exhibits two CO_2 desorption peaks at ~ 120

and 300 °C, which also appear in the CO_2-TPD profile of the present microwave-assisted prepared Zr-Ce-O with slight shift towards lower temperatures. However, additional peaks are observed starting at 400 °C and extending to temperatures higher than 600 °C. Addition of ZrO_2 induces basicity alteration on the Zr-Ce-O surface within 100-400 °C. On the other hand, MW synthesis modifies the basic sites at temperatures higher than 500 °C[65]. The higher temperature peak was not observed in commercially prepared CeO_2-ZrO_2 [64]. This can be justified by the well-established fact that synthesis process strongly affects the acid-base properties of mixed metal oxides[66].

S2.5. $^{16}O/^{18}O$ Isotopic exchange followed by CO/Ar reaction

Fig. S10 presents the transient response curves of the rate of formation of the $C^{18}O_2$ obtained over selected TM-doped ceria catalysts according to the experiment described in Section 2.4 and presented in Section 3.4.

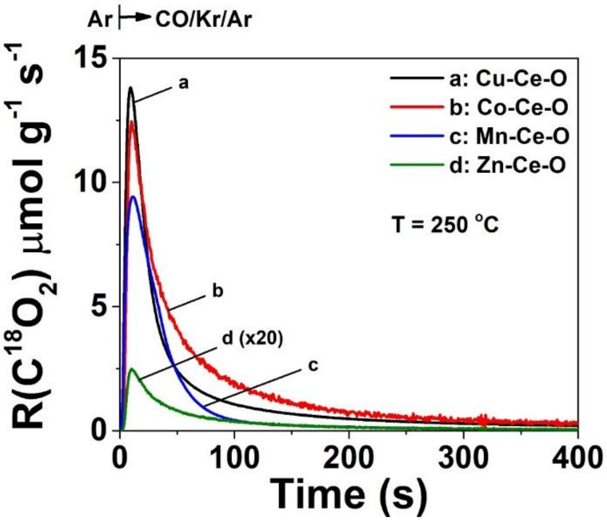

Figure S10: Transient rates (μmol g^{-1} s^{-1}) of $C^{18}O_2$ production during the step-gas switch Ar → 4 vol% CO/2 vol% Kr/Ar (20 min) performed at 250 °C on (a) Cu-Ce-O, (b) Co-Ce-O, (c) Mn-Ce-O and (d) Zn-Ce-O doped-ceria solids following $^{16}O/^{18}O$ oxygen exchange.

S2.6. SSITKA-DRIFTS studies

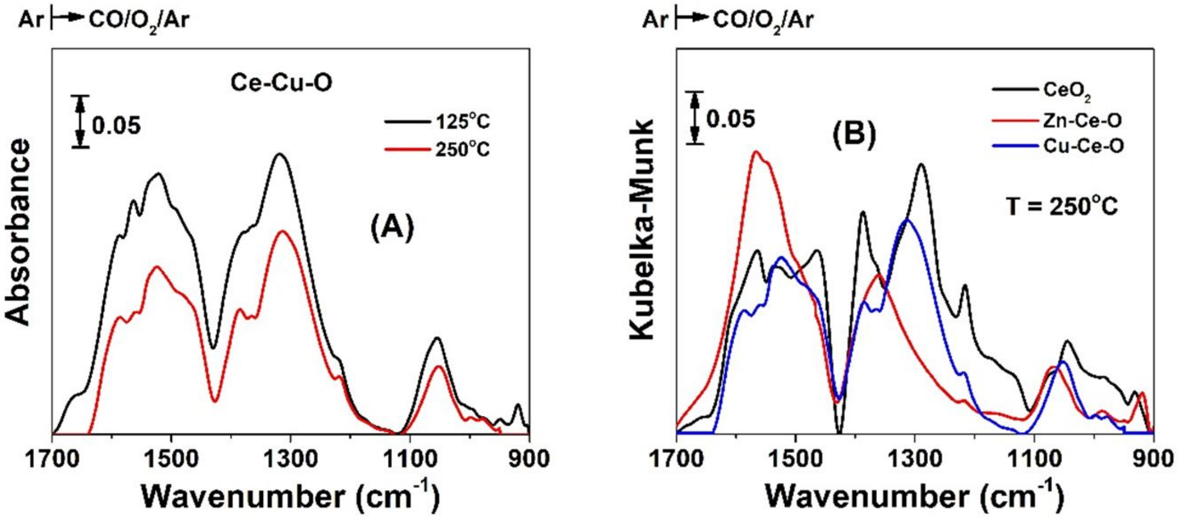

Figure S11: (A) DRIFTS spectra recorded in the 1700-900 cm^{-1} IR region after 30 min in $^{12}CO/O_2$ over the Cu-Ce-O solid at 125 or 250 ^{o}C. Feed gas composition: $^{12}CO = 4$ vol%, $O_2 = 20$ vol%, balance Ar; $F_T = 50$ NmL min^{-1}. **(B)** DRIFTS spectra recorded in the 1700–900 cm^{-1} IR region after 30 min in $^{12}CO/O_2$ over CeO_2, Zn-Ce-O and Cu-Ce-O solids. Feed gas composition: $^{12}CO = 4$ vol%, $O_2 = 20$ vol%, balance Ar; $F_T = 50$ NmL min^{-1}.

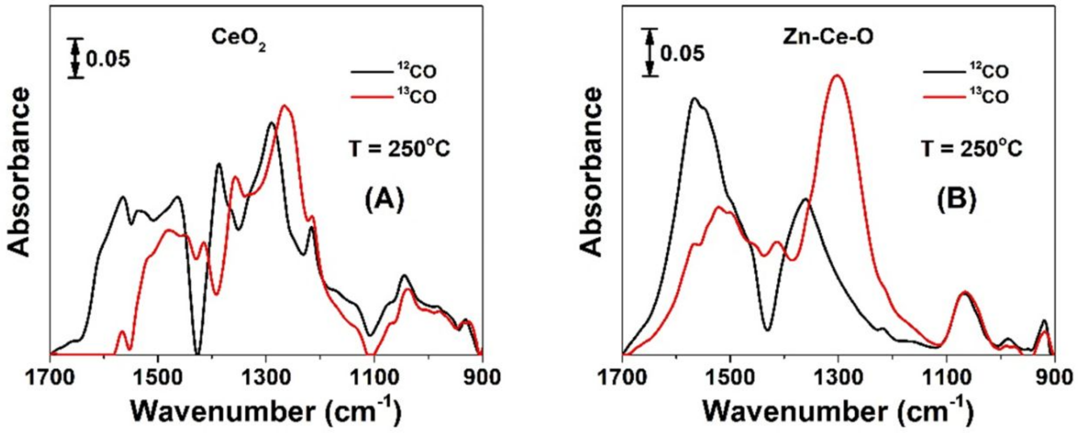

Figure S12: SSITKA-DRIFTS spectra recorded in the 1700-900 cm^{-1} region after 30 min in $^{12}CO/O_2$ at 250 ^{o}C followed by the step-gas switch $^{12}CO/O_2 \rightarrow {}^{13}CO/O_2$ (5 min) for the CeO_2 (A) and Zn-Ce-O (B) solids. Feed gas composition of non-isotopic and isotopic gas: ^{12}CO or $^{13}CO = 4$ vol%, $O_2 = 20$ vol%, balance Ar; $F_T = 50$ NmL min^{-1}.

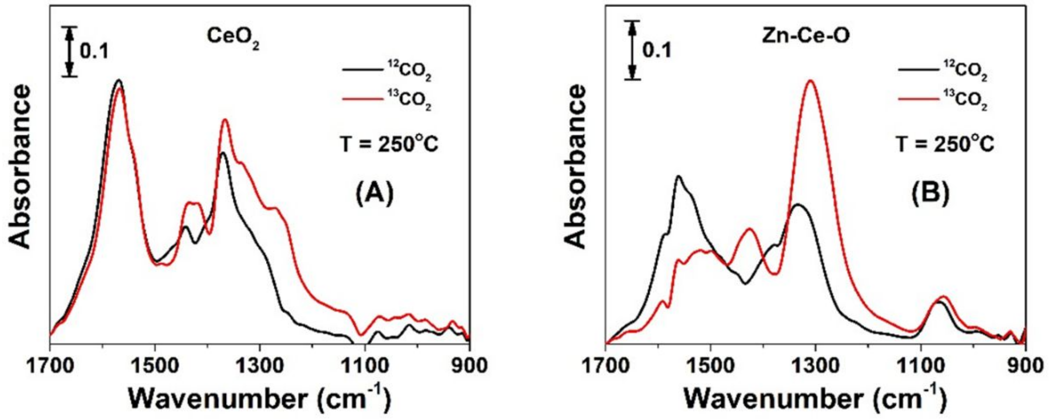

Figure S13: DRIFTS spectra recorded in the 1700-900 cm^{-1} IR region after 30 min in $^{12}CO_2/O_2/Ar$ gas mixture followed by the step-gas switch $^{12}CO_2/O_2$ /Ar → $^{13}CO_2/O_2/Ar$ (5 min) for the CeO_2 and Zn-Ce-O solids. Feed gas composition of non-isotopic and isotopic gas: $^{12}CO_2$ or $^{13}CO_2$ = 2 vol%, O_2 = 20 vol%, balance Ar; F_T = 50 NmL min^{-1}.

S2.7. Kinetic studies

S2.7.1. Estimation of Apparent Activation Energy, E_a (kJ/mol)

Table S4. Kinetic analysis of CO oxidation rate data over CeO_2 catalyst.

CeO_2					
**Temperature (°C)**	**1/T (K^{-1})**	**Catalyst mass (mg)**	**Total Flow (ml min^{-1})**	**R_{CO} (mol g^{-1} s^{-1})**	**Ln (R_{CO})**
**150**	**0.00236**	**50**	**50**	**2.72E-07**	**-15.115**
**180**	**0.00221**	**50**	**100**	**6.81E-07**	**-14.199**
**210**	**0.00207**	**50**	**100**	**1.91E-06**	**-13.169**
**240**	**0.00195**	**50**	**100**	**4.36E-06**	**-12.342**
**270**	**0.00184**	**50**	**100**	**8.83E-06**	**-11.637**

Zn-Ce-O					
**Temperature (°C)**	**1/T (K^{-1})**	**Catalyst mass (mg)**	**Total Flow (ml min^{-1})**	**R_{CO} (mol g^{-1} s^{-1})**	**Ln (R_{CO})**
**150**	**0.002363**	**50**	**50**	**3.408E-07**	**-14.892**

Table S5. Kinetic analysis of CO oxidation rate data over Zn-Ce-O catalyst.

**180**	**0.002207**	**50**	**100**	**9.542E-07**	**-13.862**
**210**	**0.002070**	**50**	**100**	**1.636E-06**	**-13.323**
**240**	**0.001949**	**50**	**100**	**3.408E-06**	**-12.589**
**270**	**0.001841**	**50**	**100**	**5.725E-06**	**-12.071**

Table S6. Kinetic analysis of CO oxidation rate data over Mn-Ce-O catalyst.

**Mn-Ce-O**					
**Temperature (^{o}C)**	**1/T (K^{-1})**	**Catalyst mass (mg)**	**Total Flow (ml min^{-1})**	**R_{CO} (mol g^{-1} s^{-1})**	**Ln (R_{CO})**
**150**	**0.002363**	**10**	**200**	**1.090E-05**	**-11.426**
**180**	**0.002207**	**10**	**200**	**2.726E-05**	**-10.51**
**210**	**0.002070**	**10**	**200**	**5.316E-05**	**-9.842**
**240**	**0.001949**	**5**	**200**	**10.36E-05**	**-9.175**
**270**	**0.001841**	**5**	**210**	**17.176E-05**	**-8.669**

Table S7. Kinetic analysis of CO oxidation rate data over Cu-Ce-O catalyst.

**Cu-Ce-O**					
**Temperature (^{o}C)**	**1/T (K^{-1})**	**Catalyst mass (mg)**	**Total Flow (ml min^{-1})**	**R_{CO} (mol g^{-1} s^{-1})**	**Ln (R_{CO})**
**120**	**0.00254**	**1**	**200**	**2.88E-04**	**-8.154**
**135**	**0.00245**	**1**	**200**	**4.77E-04**	**-7.647**
**150**	**0.00236**	**1**	**200**	**7.49E-04**	**-7.197**
**165**	**0.00228**	**1**	**200**	**1.09E-03**	**-6.826**

2.7.2. Estimation of Reaction Orders in the CO Oxidation

2.7.2.1. Reaction Order in CO (x)

Table S8. Reaction order analysis with regards to CO over the CeO_2 catalyst.

**CeO_2**					
**P_{CO}**	**1/T (K^{-1}) (T=**	**Catalyst mass (mg)**	**Total Flow (ml min^{-1})**	**R_{CO} (mol g^{-1} s^{-1})**	**Ln (R_{CO})**

	210°C)				
0.06	0.002207	50	100	2.356E-06	-12.958
0.04	0.002207	50	100	1.908E-06	-13.169
0.03	0.002207	50	100	1.630E-06	-13.326
0.02	0.002207	50	100	1.363E-06	-13.505
0.01	0.002207	50	100	9.542E-07	-13.862

Table S9. Reaction order analysis with regards to CO over the Zn-Ce-O catalyst.

Zn-Ce-O					
P_{CO}	1/T (K^{-1}) (T= 210°C)	Catalyst mass (mg)	Total Flow (ml min^{-1})	R_{CO} (mol g^{-1} s^{-1})	Ln (R_{CO})
0.06	0.002207	50	100	2.031E-06	-13.107
0.04	0.002207	50	100	1.636E-06	-13.323
0.03	0.002207	50	100	1.426E-06	-13.460
0.02	0.002207	50	100	1.227E-06	-13.611
0.01	0.002207	50	100	8.860E-07	-13.936

Table S10. Reaction order analysis with regards to CO over the Mn-Ce-O catalyst.

Mn-Ce-O					
P_{CO}	1/T (K^{-1}) (T= 210°C)	Catalyst mass (mg)	Total Flow (ml min^{-1})	R_{CO} (mol g^{-1} s^{-1})	Ln (R_{CO})
0.06	0.002207	10	200	7.034E-05	-9.562
0.04	0.002207	10	200	5.316E-05	-9.842
0.03	0.002207	10	200	4.457E-05	-10.018
0.02	0.002207	10	200	3.762E-05	-10.188
0.01	0.002207	10	200	2.426E-05	-10.626

Table S11. Reaction order analysis with regards to CO over the Cu-Ce-O catalyst.

Cu-Ce-O					
P_{CO}	1/T (K^{-1}) (T=150°C)	Catalyst mass (mg)	Total Flow (ml min^{-1})	R_{CO} (mol g^{-1} s^{-1})	Ln (R_{CO})
0.06	0.002363	1	200	1.226E-03	-6.703

**0.04**	**0.002363**	**1**	**200**	**0.752E-03**	**-7.192**
**0.03**	**0.002363**	**1**	**200**	**0.557E-03**	**-7.492**
**0.02**	**0.002363**	**1**	**200**	**0.327E-03**	**-8.025**
**0.01**	**0.002363**	**1**	**200**	**0.149E-03**	**-8.805**

2.7.2.2. Reaction Order in O_2 (y)

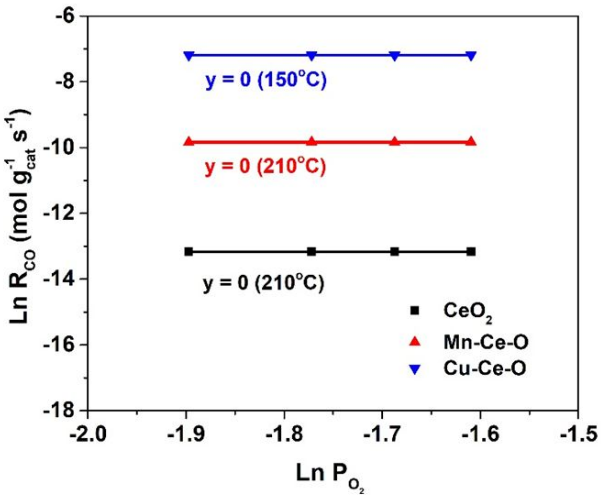

Figure S14: Determination of the reaction order n_{O2} (or y) with respect to the O_2 partial pressure in the CO oxidation reaction at T=210°C for Mn-Ce-O and CeO_2 solids, and at T=150°C for the Cu-Ce-O solid. The partial pressure of O_2 was varied in the 0.15 t- 0.2 bar range.

Table S12. Reaction order analysis with regards to O_2 over the CeO_2 catalyst.

**CeO_2**					
**P_{O2} (bar)**	**1/T (K^{-1}) T= 210°C**	**Catalyst mass (mg)**	**Total Flow (ml min^{-1})**	**R_{CO} (mol g^{-1} s^{-1})**	**Ln (R_{CO})**
**0.200**	**0.002207**	**50**	**100**	**1.908E-06**	**-13.169**
**0.185**	**0.002207**	**50**	**100**	**1.908E-06**	**-13.169**
**0.170**	**0.002207**	**50**	**100**	**1.908E-06**	**-13.169**
**0.150**	**0.002207**	**50**	**100**	**1.908E-06**	**-13.169**

Table S13. Reaction order analysis with regards to O_2 over the Mn-Ce-O catalyst.

**Mn-Ce-O**

P_{O2} (bar)	1/T (K^{-1}) T= 210°C	Catalyst mass (mg)	Total Flow (ml min^{-1})	R_{CO} (mol g^{-1} s^{-1})	Ln (R_{CO})
**0.200**	**0.002207**	**10**	**200**	**5.31E-05**	**-9.842**
**0.185**	**0.002207**	**10**	**200**	**5.31E-05**	**-9.842**
**0.170**	**0.002207**	**10**	**200**	**5.31E-05**	**-9.842**
**0.150**	**0.002207**	**10**	**200**	**5.31E-05**	**-9.842**

Table S14. Reaction order analysis with regards to O_2 over the Cu-Ce-O catalyst.

Cu-Ce-O					
P_{O2} (bar)	1/T (K^{-1}) T= 210°C	Catalyst mass (mg)	Total Flow (ml min^{-1})	R_{CO} (mol g^{-1} s^{-1})	Ln (R_{CO})
**0.200**	**0.002363**	**1**	**200**	**7.52E-4**	**-7.192**
**0.185**	**0.002363**	**1**	**200**	**7.52E-4**	**-7.192**
**0.170**	**0.002363**	**1**	**200**	**7.52E-4**	**-7.192**
**0.150**	**0.002363**	**1**	**200**	**7.52E-4**	**-7.192**

References

(1) F. Rouquerol, J. Rouquerol, K.S.W. Sing, P. Liewellyn, G. M. *Adsorption by Powders and Porous Solids*, 2nd ed.; Elsevier Inc., **2014**

(2) Dal Corso, A. Projector Augmented-Wave Method: Application to Relativistic Spin-Density Functional Theory. Phys. Rev. B - Condens. Matter Mater. Phys. **2010**, 82 (7), 1–18. https://doi.org/10.1103/PhysRevB.82.075116.

(3) Perdew, J. P.; Burke, K.; Ernzerhof, M. Generalized Gradient Approximation Made Simple. Phys. Rev. Lett. **1996**, 77 (18), 3865–3868. https://doi.org/10.1103/PhysRevLett.77.3865.

(4) Nolan, M.; Parker, S. C.; Watson, G. W. The Electronic Structure of Oxygen Vacancy Defects at the Low Index Surfaces of Ceria. Surf. Sci. **2005**, 595 (1–3), 223–232. https://doi.org/10.1016/j.susc.2005.08.015.

(5) Fabris, S.; Vicario, G.; Balducci, G.; De Gironcoli, S.; Baroni, S. Electronic and Atomistic Structures of Clean and Reduced Ceria Surfaces. J. Phys. Chem. B **2005**, 109 (48), 22860–22867. https://doi.org/10.1021/jp0511698.

(6) Nolan, M.; Grigoleit, S.; Sayle, D. C.; Parker, S. C.; Watson, G. W. Density Functional Theory Studies of the Structure and Electronic Structure of Pure and Defective Low Index Surfaces of Ceria. Surf. Sci. **2005**, 576 (1–3), 217–229. https://doi.org/10.1016/j.susc.2004.12.016
(7) Sing, K. S. W. REPORTING PHYSISORPTION DATA FOR GAS/SOLID SYSTEMS with Special Reference to the Determination of Surface Area and Porosity. *Pure Appl. Chem.* **1985**, *57* (4), 603–619.
(8) Gelb, L. D.; Gubbins, K. E. Pore Size Distributions in Porous Glasses: A Computer Simulation Study. *Langmuir* **1999**. https://doi.org/10.1021/la9808418.
(9) Laguna, O. H.; Pérez, A.; Centeno, M. A.; Odriozola, J. A. Synergy between Gold and Oxygen Vacancies in Gold Supported on Zr-Doped Ceria Catalysts for the CO Oxidation. Appl. Catal. B Environ. **2015**, 176–177, 385–395. https://doi.org/10.1016/j.apcatb.2015.04.019
(10) Atribak, I.; Bueno-López, A.; García-García, A. Combined Removal of Diesel Soot Particulates and NOx over CeO2-ZrO2 Mixed Oxides. J. Catal. **2008**, 259 (1), 123–132. https://doi.org/10.1016/j.jcat.2008.07.016
(11) Takalkar, G.D.; Bhosale, R.R.; Kumar, A.; AlMomani, F.; Khraisheha, M.; Shakoorb, R.A.;. Gupta R.B. Transition metal doped ceria for solar thermochemical fuel production, Solar Energy **2018**, 172, 204-211. https://doi.org/10.1016/j.solener.2018.03.022
(12) Jampaiah, D. Tur, K.M.; Ippolito, S.J.; Sabri, Y.M.; Tardio, J. Structural Chracterization and catalytic evaluation of transition and rare earth metal doped ceria-based solid solutions for elemental mercury oxidation RSC Advances, **2013**, 3, 12963. DOI: 10.1039/c3ra41441h
(13) Kang, C.H.Y.; KusabaH.;, Yahiro, H.; Sasaki, K.; Teraok, Y. Preparation, characterization and electrical property of Mn-doped ceria-based oxides. Solid State Ionics **2006**, 177, 1799–1802 doi:10.1016/j.ssi.2006.04.016
(14) Venkataswamy, P.; Jampaiah, D.; · Kandjani, A.E.; ·. Sabri, Y.M.; · Reddy, B.M. · Vithal, M.; Transition (Mn, Fe) and rare earth (La, Pr) metal doped ceria solid solutions for high performance photocatalysis: Effect of metal doping on catalytic activity. Res Chem Intermed **2018**, 44, 2523–2543. https://doi.org/10.1007/ s11164-017-3244-5
(15) Kim, H.J.; Lee, G.; Jang, M.G.; Noh, K.J., Han. J.W. ChemCatChem **2019**, 11, 2288 – 2296. Rational Design of Transition Metal Co-Doped Ceria Catalysts for Low-Temperature CO Oxidation. https://doi.org/10.1002/cctc.201900178
(16) Zhan, W.; Yang, S.; Zhang, P.; Guo, Y.; Lu, G.; Chisholm, M.F.; Dai, S. Incorporating Rich Mesoporosity into a Ceria-Based Catalyst via Mechanochemistry. Chem. Mater. **2017**, 29, 7323−7329. https://doi.org/10.1021/acs.chemmater.7b02206 Jae-ha Myung IJHE 40 (2015) 12003-12008
(17) Mukherjee, D.; . Rao, B.G.; Reddy, B.M. CO and soot oxidation activity of doped ceria: Influence of dopants. Applied Catalysis B: Environmental **2016**, 197,105–115. https://doi.org/10.1016/j.apcatb.2016.03.042
(18) Venkataswamy, P.; Rao, K.N; Jampaiah, D.; Reddy, B. M.; Nanostructured manganese doped ceria solid solutions for CO oxidation at lower temperatures, Applied Catalysis B: Environmental **2015**, 162, 122–132. http://dx.doi.org/10.1016/j.apcatb.2014.06.038
(19) Zhang, J.; Guo, J.; Liu, W.; Wang, S.; Xie, A.; Liu,X.; Wang, J.; Yang, Y.Facile

Preparation of Mn+-Doped (M = Cu, Co, Ni, Mn) Hierarchically Mesoporous CeO2 Nanoparticles with Enhanced Catalytic Activity for CO Oxidation Eur. J. Inorg. Chem. **2015**, 969–976. http://dx.doi.org/10.1002/ejic.201403078.

(20) Samuel Taub, PhD Thesis 2013, Department of Materials, Imperial College London

(21) Elias, J.S.; Stoerzinger, K.A.; Hong, W.T.; Risch, M.; Giordano, L.; Mansour, A.N.; Shao-Horn,Y. In Situ Spectroscopy and Mechanistic Insights into CO Oxidation on Transition-Metal-Substituted Ceria Nanoparticles ACS Catal. **2017**, 7, 6843−6857. DOI: 10.1021/acscatal.7b01600

(22) Li, L.; Chen, F.; -Qing Lu, Q.; Luo, M.F. Study of Defect Sites in Ce1 xMxO2 δ (x = 0.2) Solid Solutions Using Raman Spectroscopy, J. Phys. Chem. A **2011**, 115, 7972–7977. dx.doi.org/10.1021/jp203921m

(23) Filtschew, A.; Hofmann, K.; Christian Hess, Ceria and Its Defect Structure: New Insights from a Combined Spectroscopic Approach, J. Phys. Chem. C **2016**, 120, 6694−6703. DOI: 10.1021/acs.jpcc.6b00959

(24) Mandal, B. P. ;Roy, M.; Grover, V.; Tyagi, A. K. X-ray diffraction, -Raman spectroscopic studies on (RE=Ho, Er) systems: Observation of parasitic phases. J. Appl. Phys. **2008**, 103, 033506. https://doi.org/10.1063/1.2837042.

(25) Enrico Sartoretti,E.; Novara, C.; Giorgis, F.; Piumetti, M.; Bensaid, S.; Russo N.; & Fino, D. In situ Raman analyses of the soot oxidation reaction over nanostructured ceria-based catalysts. Scientific Reports **2019**, 9, 3875 | https://doi.org/10.1038/s41598-019-39105-5

(26) Zhu, C.; Osherov, A.; Panzer, M. J. Surface Chemistry of Electrodeposited Cu2O Films Studied by XPS. Electrochim. Acta **2013**, 111, 771–778. https://doi.org/10.1016/j.electacta.2013.08.038.

(27) Aleksandra Kocijan, Ingrid Milosev, B. P. Cobalt-Based Alloys for Orthopedic Applications Studied by Electrochemical and XPS Analysis. *J. Mater. Sci. Mater. Med.* **2004**, *15*, 643–650.

(28) White, B.; Yin, M.; Hall, A.; Le, D.; Stolbov, S.; Rahman, T.; Turro, N.; O'Brien, S. Complete CO Oxidation over Cu2O Nanoparticles Supported on Silica Gel. Nano Lett. **2006**, 6 (9), 2095–2098. https://doi.org/10.1021/nl061457v.

(29) Biesinger, M. C.; Payne, B. P.; Lau, L. W. M.; Gerson, A.; Smart, R. S. C. X-Ray Photoelectron Spectroscopic Chemical State Quantification of Mixed Nickel Metal, Oxide and Hydroxide Systems. Surf. Interface Anal. **2009**, 41 (4), 324–332. https://doi.org/10.1002/sia.3026.

(30) Anandha Babu, G.; Ravi, G.; Navaneethan, M.; Arivanandhan, M.; Hayakawa, Y. An Investigation of Flower Shaped NiO Nanostructures by Microwave and Hydrothermal Route. J. Mater. Sci. Mater. Electron. **2014**, 25 (12), 5231–5240. https://doi.org/10.1007/s10854-014-2293-4.

(31) Hallam, P. M.; Gómez-Mingot, M.; Kampouris, D. K.; Banks, C. E. Facile Synthetic Fabrication of Iron Oxide Particles and Novel Hydrogen Superoxide Supercapacitors. RSC Adv. **2012**, 2 (16), 6672–6679. https://doi.org/10.1039/c2ra01139e.

(32) Osowiecki, W. T.; Sheehan, S. W.; Young, K. J.; Durrell, A. C.; Mercado, B. Q.; Brudvig, G. W. Surfactant-Mediated Electrodeposition of a Water-Oxidizing Manganese Oxide. Dalt. Trans. **2015**, 44 (38), 16873–16881. https://doi.org/10.1039/c5dt02390d.

(33) Klein, J. C.; Hercules, D. M. Surface Characterization of Model Urushibara Catalysts. J. Catal. **1983**, 82 (2), 424–441. https://doi.org/10.1016/0021-9517(83)90209-9.

(34) Caputo, T.; Lisi, L.; Pirone, R.; Russo, G. On the Role of Redox Properties of CuO/CeO2 Catalysts in the Preferential Oxidation of CO in H2-Rich Gases. Appl. Catal. A Gen. **2008**, 348 (1), 42–53. https://doi.org/10.1016/j.apcata.2008.06.025.
(35) Zhou, G.; Lan, H.; Gao, T.; Xie, H. Influence of Ce/Cu Ratio on the Performance of Ordered Mesoporous CeCu Composite Oxide Catalysts. Chem. Eng. J. **2014**, 246, 53–63. https://doi.org/10.1016/j.cej.2014.02.059.
(36) Carabineiro, S. A. C.; Chen, X.; Konsolakis, M.; Psarras, A. C.; Tavares, P. B.; Órfão, J. J. M.; Pereira, M. F. R.; Figueiredo, J. L. Catalytic Oxidation of Toluene on Ce-Co and La-Co Mixed Oxides Synthesized by Exotemplating and Evaporation Methods. Catal. Today **2015**, 244, 161–171. https://doi.org/10.1016/j.cattod.2014.06.018.
(37) Ramírez-Cabrera, E.; Atkinson, A.; Chadwick, D. Reactivity of Ceria, Gd- and Nb-Doped Ceria to Methane. Appl. Catal. B Environ. **2002**, 36 (3), 193–206. https://doi.org/10.1016/S0926-3373(01)00299-5.
(38) Polychronopoulou, K.; Zedan, A. F.; Katsiotis, M. S.; Baker, M. A.; AlKhoori, A. A.; AlQaradawi, S. Y.; Hinder, S. J.; AlHassan, S. Rapid Microwave Assisted Sol-Gel Synthesis of CeO2 and CexSm1-XO2 Nanoparticle Catalysts for CO Oxidation. Mol. Catal. **2017**, 428, 41–55. https://doi.org/10.1016/j.molcata.2016.11.039.
(39) Trovarelli, A.; Llorca, J. Ceria Catalysts at Nanoscale: How Do Crystal Shapes Shape Catalysis? ACS Catal. **2017**, 7 (7), 4716–4735. https://doi.org/10.1021/acscatal.7b01246.
(40) Shan, W.; Feng, Z.; Li, Z.; Zhang, J.; Shen, W.; Li, C. Oxidative Steam Reforming of Methanol on Ce0.9Cu0.1OY Catalysts Prepared by Deposition-Precipitation, Coprecipitation, and Complexation-Combustion Methods. J. Catal. **2004**, 228 (1), 206–217. https://doi.org/10.1016/j.jcat.2004.07.010.
(41) Aranda, A.; Agouram, S.; López, J. M.; Mastral, A. M.; Sellick, D. R.; Solsona, B.; Taylor, S. H.; García, T. Oxygen Defects: The Key Parameter Controlling the Activity and Selectivity of Mesoporous Copper-Doped Ceria for the Total Oxidation of Naphthalene. *Appl. Catal. B Environ.* **2012**, *127*, 77–88. https://doi.org/10.1016/j.apcatb.2012.07.033.
(42) Polster, C. S.; Nair, H.; Baertsch, C. D. Study of Active Sites and Mechanism Responsible for Highly Selective CO Oxidation in H2 Rich Atmospheres on a Mixed Cu and Ce Oxide Catalyst. J. Catal. **2009**, 266 (2), 308–319. https://doi.org/10.1016/j.jcat.2009.06.021.
(43) Djinović, P.; Batista, J.; Pintar, A. Calcination Temperature and CuO Loading Dependence on CuO-CeO2 Catalyst Activity for Water-Gas Shift Reaction. Appl. Catal. A Gen. **2008**, 347 (1), 23–33. https://doi.org/10.1016/j.apcata.2008.05.027.
(44) Laguna, O. H.; Centeno, M. A.; Romero-Sarria, F.; Odriozola, J. A. Oxidation of CO over Gold Supported on Zn-Modified Ceria Catalysts. Catal. Today **2011**, 172 (1), 118–123. https://doi.org/10.1016/j.cattod.2011.02.015.
(45) Laguna, O. H.; Romero Sarria, F.; Centeno, M. A.; Odriozola, J. A. Gold Supported on Metal-Doped Ceria Catalysts (M = Zr, Zn and Fe) for the Preferential Oxidation of CO (PROX). J. Catal. **2010**, 276 (2), 360–370. https://doi.org/10.1016/j.jcat.2010.09.027.
(46) Luhui, W.; Shaoxing, Z.; Yuan, L. Reverse Water Gas Shift Reaction over Co-Precipitated Ni-Ce02 Catalysts. J. Rare Earths **2007**, 26 (20476079), 66–70.
(47) Shan, W.; Luo, M.; Ying, P.; Shen, W.; Li, C. Reduction Property and Catalytic Activity of Ce1-XNiXO2 Mixed Oxide Catalysts for CH4 Oxidation. Appl. Catal. A Gen. **2003**, 246 (1), 1–9. https://doi.org/10.1016/S0926-860X(02)00659-2.

(48) Gonzalez-DelaCruz, V. M.; Holgado, J. P.; Pereñíguez, R.; Caballero, A. Morphology Changes Induced by Strong Metal-Support Interaction on a Ni-Ceria Catalytic System. J. Catal. **2008**, 257 (2), 307–314. https://doi.org/10.1016/j.jcat.2008.05.009.
(49) Roh, H. S.; Jun, K. W.; Dong, W. S.; Chang, J. S.; Park, S. E.; Joe, Y. Il. Highly Active and Stable Ni/Ce-ZrO2 Catalyst for H2 Production from Methane. J. Mol. Catal. A Chem. **2002**, 181 (1–2), 137–142. https://doi.org/10.1016/S1381-1169(01)00358-2.
(50) R. Brown, M.E. Cooper, D. A. W. Temperature Programmed Reduction of Aloumina Supported Iron, Cobalt and Nickel Bimetallic Catalysts. Appl. Catal. **1982**, 3, 177–186.
(51) Li, S.; Krishnamoorthy, S.; Li, A.; Meitzner, G. D.; Iglesia, E. Promoted Iron-Based Catalysts for the Fischer-Tropsch Synthesis: Design, Synthesis, Site Densities, and Catalytic Properties. J. Catal. **2002**, 206 (2), 202–217. https://doi.org/10.1006/jcat.2001.3506.
(52) Lan, L.; Chen, S.; Cao, Y.; Zhao, M.; Gong, M.; Chen, Y. Preparation of Ceria-Zirconia by Modified Coprecipitation Method and Its Supported Pd-Only Three-Way Catalyst. J. Colloid Interface Sci. **2015**, 450, 404–416. https://doi.org/10.1016/j.jcis.2015.03.042.
(53) DE LEITENBURG, C.; TROVARELLI, A.; ZAMAR, F.; MASCHIO, S.; DOLCETTI, G.; LLORCA, J. ChemInform Abstract: A Novel and Simple Route to Catalysts with a High Oxygen Storage Capacity: The Direct Room-Temperature Synthesis of CeO2-ZrO2 Solid Solutions. ChemInform **2010**. https://doi.org/10.1002/chin.199606012.
(54) Venkataswamy, P.; Rao, K. N.; Jampaiah, D.; Reddy, B. M. Nanostructured Manganese Doped Ceria Solid Solutions for CO Oxidation at Lower Temperatures. Appl. Catal. B Environ. **2015**, 162, 122–132. https://doi.org/10.1016/j.apcatb.2014.06.038.
(55) Mandal, S.; Santra, C.; Bando, K. K.; James, O. O.; Maity, S.; Mehta, D.; Chowdhury, B. Aerobic Oxidation of Benzyl Alcohol over Mesoporous Mn-Doped Ceria Supported Au Nanoparticle Catalyst. J. Mol. Catal. A Chem. **2013**, 378, 47–56. https://doi.org/10.1016/j.molcata.2013.05.011.
(56) Zou, Z. Q.; Meng, M.; Zha, Y. Q. Surfactant-Assisted Synthesis, Characterizations, and Catalytic Oxidation Mechanisms of the Mesoporous MnOx-CeO2 and Pd/MnOx-CeO2 Catalysts Used for CO and C3H8 Oxidation. J. Phys. Chem. C **2010**, 114 (1), 468–477. https://doi.org/10.1021/jp908721a.
(57) Poggio Fraccari, E.; D'Alessandro, O.; Sambeth, J.; Baronetti, G.; Mariño, F. Ce-Mn Mixed Oxides as Supports of Copper- and Nickel-Based Catalysts for Water-Gas Shift Reaction. Fuel Process. Technol. **2014**, 119, 67–73. https://doi.org/10.1016/j.fuproc.2013.10.012.
(58) Fang, P.; Luo, M. F.; Lu, J. Q.; Cen, S. Q.; Yan, X. Y.; Wang, X. X. Studies on the Oxidation Properties of Nanopowder CeO2-Based Solid Solution Catalysts for Model Soot Combustion. Thermochim. Acta **2008**, 478 (1–2), 45–50. https://doi.org/10.1016/j.tca.2008.08.017.
(59) Tada, S.; Shimizu, T.; Kameyama, H.; Haneda, T.; Kikuchi, R. Ni/CeO2 Catalysts with High CO2 Methanation Activity and High CH4 Selectivity at Low Temperatures. Int. J. Hydrogen Energy **2012**, 37 (7), 5527–5531. https://doi.org/10.1016/j.ijhydene.2011.12.122.
(60) Hong, W. J.; Iwamoto, S.; Inoue, M. Direct NO Decomposition over a Ce-Mn

Mixed Oxide Modified with Alkali and Alkaline Earth Species and CO2-TPD Behavior of the Catalysts. Catal. Today **2011**, 164 (1), 489–494. https://doi.org/10.1016/j.cattod.2010.10.063.

(61) Neri, G.; Bonavita, A.; Rizzo, G.; Galvagno, S.; Capone, S.; Siciliano, P. Methanol Gas-Sensing Properties of CeO2-Fe2O 3 Thin Films. Sensors Actuators, B Chem. **2006**, 114 (2), 687–695. https://doi.org/10.1016/j.snb.2005.06.062.

(62) Ba, B.; Rodr, I.; Gu, A. In Te Ra c Tio n o f Ca Rbo n D Io x Id e w Ith Th e S u Rfa c e o f Zirc o n Ia P o Ly m o Rp h S. **1998**, 7463 (14), 3556–3564.

(63) Ozawa, M.; Yuzuriha, H.; Haneda, M. Total Oxidation of Toluene and Oxygen Storage Capacity of Zirconia-Sol Modified Ceria Zirconia. Catal. Commun. **2013**, 30, 32–35. https://doi.org/10.1016/j.catcom.2012.10.008.

(64) Reddy, B. M.; Reddy, G. K.; Katta, L. Structural Characterization and Dehydration Activity of CeO2-SiO2 and CeO2-ZrO2 Mixed Oxides Prepared by a Rapid Microwave-Assisted Combustion Synthesis Method. J. Mol. Catal. A Chem. **2010**, 319 (1–2), 52–57. https://doi.org/10.1016/j.molcata.2009.11.020.

(65) Su, C.; Li, J.; He, D.; Cheng, Z.; Zhu, Q. Synthesis of Isobutene from Synthesis Gas over Nanosize Zirconia Catalysts. Appl. Catal. A Gen. **2000**, 202 (1), 81–89. https://doi.org/10.1016/S0926-860X(00)00461-0.
